# Emerging and regionally novel voltage-gated sodium channel mutations associated with pyrethroid resistance and altered binding affinity in *Aedes albopictus* (Skuse) from Northern Peninsular Malaysia

**DOI:** 10.1186/s13071-026-07496-w

**Published:** 2026-06-21

**Authors:** Nurul Adilah-Amrannudin, Kenneth Tan JunKai, Ahmad Faisal Mohamad, Abu Hassan Ahmad, Hasber Salim, Ghows Azzam, Nurul Nadia Manap, Shamsudin Rahman, Intan Haslina Ishak

**Affiliations:** 1 Insecticide Resistance Research Group (IRRG), School of Biological Sciences, Universiti Sains Malaysia, 11800 Minden, Penang Malaysia; 2 Vector Control Research Unit, School of Biological Sciences, Universiti Sains Malaysia, 11800 Minden, Penang Malaysia; 3 School of Biological Sciences, Universiti Sains Malaysia, 11800 Minden, Penang Malaysia; 4 School of Medical Science, Universiti Sains Malaysia, Kubang Kerian, 15200 Kota Bharu, , Kelantan Malaysia; 5 Entomology and Pest Unit, Kangar District Health Office, Jalan Abi Tok Hashim, 01000 Kangar, Perlis Malaysia

**Keywords:** *Aedes albopictus*, Pyrethroid, Insecticide resistance, *Kdr* mutation, Genetic variation, In silico docking

## Abstract

**Background:**

The application of synthetic insecticides is the primary means of controlling *Aedes* mosquitoes in Malaysia, but the efficacy of this method is undermined by the evolution of resistance. *Aedes albopictus* as one of the dominant and competent arboviral vectors has an elusive insecticide resistance status in different geographical regions of Northern Peninsular Malaysia. This underscores the importance of assessing the diverse types of insecticides used and their association with target-site resistance mechanism in this species, which forms the basis of the present study.

**Methods:**

WHO bioassays were performed on *Ae. albopictus* larvae and adults from four localities (Penang and Perlis), towards 0.034 ppm temephos, 0.25% permethrin, 0.03% deltamethrin, 0.25% pirimiphos-methyl, and 0.1% propoxur. The partial *voltage-gated sodium channel* (*vgsc*) gene domain (DIIS6, DIIIS6, and DIVS6) of pyrethroid-exposed samples were subsequently genotyped through direct sequencing for single-nucleotide mutations, together with genetic variations and haplotype networks analysis. The predicted protein structures for the mutated regions and their binding affinities to pyrethroids were also evaluated using in silico docking.

**Results and discussions:**

Varying degrees of resistance were observed in all Penang and Perlis strains to all tested insecticides. Moreover, the detection of the F1534L mutation and newly discovered non-synonymous mutations (A1022S/P, E1041K, P1585R, and F1695L) suggest the progression of resistance alleles dissemination in these strains. The analysis of genetic variations, resistance allele distribution patterns, and haplotype networks showed evidence for multiple origins of these mutations. Data also revealed the discovered mutations affect the affinity of *vgsc*-binding proteins to pyrethroids.

**Conclusions:**

This study highlights the genotype–phenotype associations in *Ae. albopictus* and their genetic links to pyrethroid resistance, offering insights to strengthen vector control strategies in Malaysia.

**Graphical Abstract:**

**Figure:**
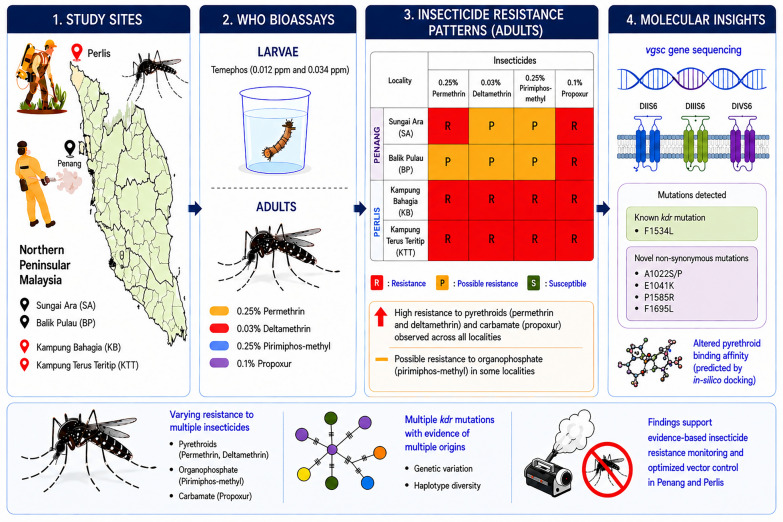

**Supplementary Information:**

The online version contains supplementary material available at 10.1186/s13071-026-07496-w.

## Background

*Aedes albopictus* (Skuse), one of the most invasive mosquito species in tropical and subtropical regions, serves as a primary vector for dengue, chikungunya, and Zika virus [1]. *Aedes albopictus*, second only to *Ae. aegypti* in its role as dengue virus vector inflicts the disease onto millions of people in tropical and subtropical regions worldwide [2]. However, *Ae. albopictus* is gaining significance as a vector, with dengue virus detected in this arboreal mosquito, including in Malaysia [3]. Although cumulative dengue cases decreased from 122,423 in 2024 to 55,399 in 2025 following the implementation of the Dengue-Free Community (KomBeD) initiative and expanded *Wolbachia* mosquito operations, the threat nevertheless persists [4]. This species also possesses strong ecological adaptability, coupled with its ability to thrive near human dwellings, survive transport of its cold-hardy and drought-resistant eggs, and live in close association with humans, further underscores its importance [5], particularly on its invasiveness and ability to displace *Ae. aegypti*.

Despite the introduction of Qdenga, the latest licensed dengue vaccine in Malaysia which is still in the process of gaining public confidence, vector control through the use of synthetic insecticide remains the gold standard method for controlling *Aedes* mosquitoes’ densities in the country [6–8]. In Malaysia, vector control programmes often involve the use of pyrethroids, organophosphates, and carbamates, a practice carried out by the Ministry of Health, private pest control companies, and local communities [9, 10] as well as in the agrarian sector [11]. These insecticides target the insect’s nervous system, albeit through different mechanisms, ultimately causing its death [12]. However, their prolonged and excessive use has fostered an overreliance on these insecticides, which has contributed to the emergence of resistance in the mosquito vector [13].

The extensive use of insecticides is a major driver behind the growing reports of insecticide resistance in *Aedes* populations both in Malaysia and globally [14], jeopardizing the efficiency of existing vector control measures. In Malaysia, the primary vector, *Ae. aegypti*, has been the focus of numerous local studies in epidemic-prone cities [7, 15–18]. These studies have sparked interest in *Ae. albopictus* populations from various states, including Kuala Lumpur [8, 16], Selangor [8, 19], Perak [17], Sabah [20], as well as Pahang and Johor [8], highlighting the lack of comprehensive data on strains from Northern Peninsular Malaysia. This region, with its agricultural landscapes and diverse economic activities bordering Thailand, is prone to *Ae. albopictus* infestations in both urban and rural areas. Extensive insecticide use in these regions raises concerns about the emergence and evolution of insecticide resistance within this species.

Insecticide resistance primarily arises from two factors: changes in target sites and an increase in the rate of insecticide metabolism [21]. Metabolic resistance is mainly driven by three enzyme families, namely, cytochrome P450s, esterases, and glutathione S-transferases (GSTs), while target-site resistance is typically caused by one or more mutations in the insecticide's target site [13, 22]. A key mutation linked to target-site resistance is the 'knockdown resistance' (*kdr*) mutation in the *voltage-gated sodium channel* (*vgsc*) gene, which has been increasingly evolving in *Ae. albopictus*. This includes the emergence of V410L/A/G in Thailand and Italy [23], V1016G in China [24, 25], India [26], and Europe [27], F1534S/C/L/W/I/R in Singapore [28], Indonesia [29], and China [23, 30–33], and I1532T in China [25, 31, 34], all of which are putatively confers resistance to Dichlorodiphenyltrichloroethane (DDT) and pyrethroid insecticides.

Pyrethroid resistance is a major concern in Malaysia, where *Ae. albopictus* has been reported to have two identified mechanisms of resistance: target-site resistance (knockdown resistance, *kdr*) [19] and metabolic resistance [35]. The null detection of *kdr* mutations in *Ae. albopictus* from Malaysian in 2015 [16] was overturned five years later with the first discovery of the F1534L mutation in this species [19]. Even so, the genotypic screening of *kdr* mutations in Malaysian *Ae. albopictus* remains poorly defined despites various attempts at insecticide susceptibility profiling across different geographic regions of this species over time. Currently, there have been no attempts to investigate the distribution of alleles, the burden of *vgsc* mutations, or their impact on pyrethroid’s binding affinity in *Ae. albopictus* from Malaysia, limiting our understanding of factors driving pyrethroid resistance. Such fundamental insights are essential as an integral component of vector control programmes and a prerequisite for effective interventions, forming the foundation of the present study on *Ae. albopictus* in Northern Peninsular Malaysia.

## Methods

### Study site

**Fig. 1:**
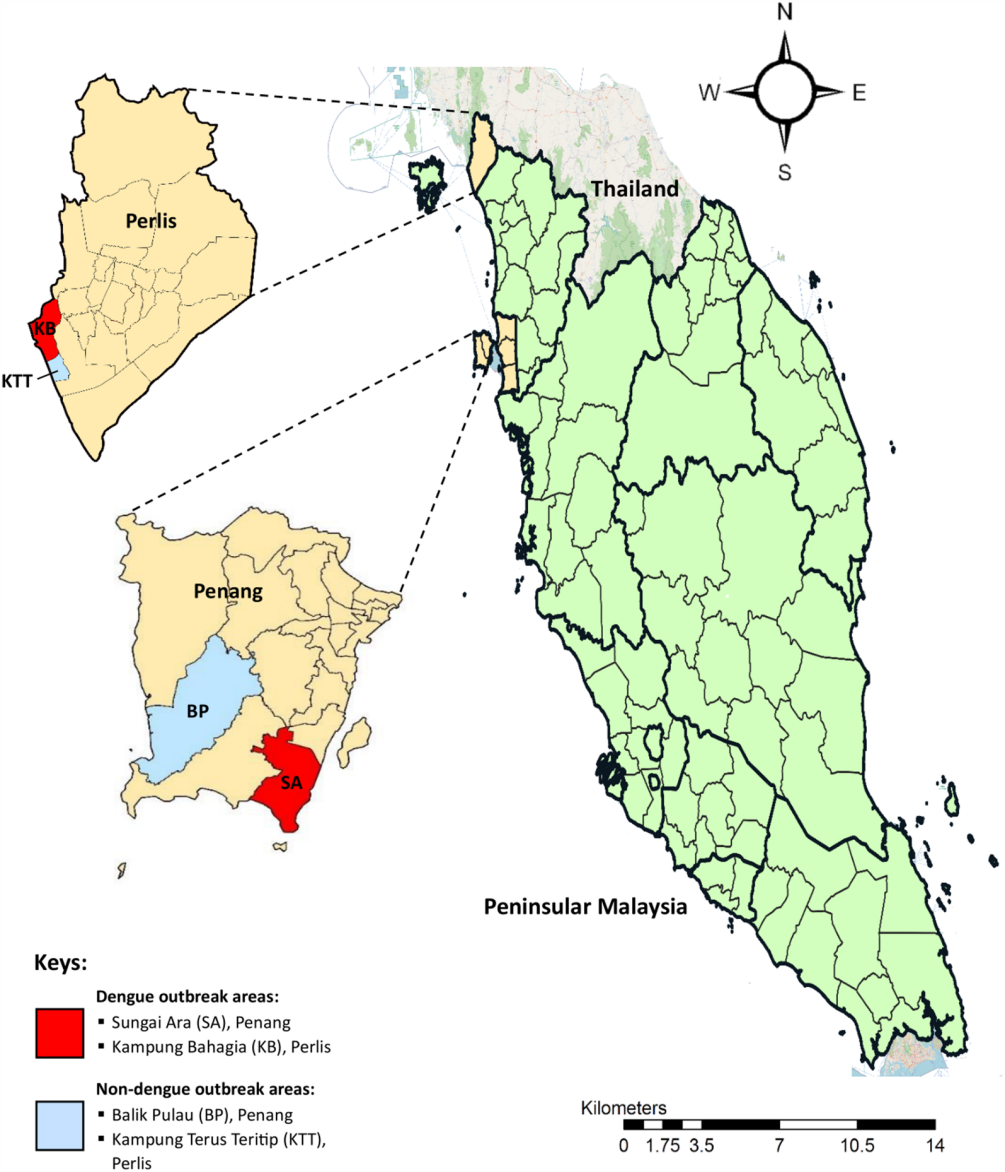
Geographical location of the study. The map of the studied locations in Northern Peninsular Malaysia was created using QGIS v3.38.3, with the base map adapted from open data provided by Natural Earth (https://www.naturalearthdata.com/; public domain)

Field collections were conducted in residential areas of Northern Peninsular Malaysia: Penang (Sungai Ara, Balik Pulau) and Perlis (Kampung Bahagia, Kampung Terus Teritip) (Fig. 1). In each state, one dengue outbreak and one non-outbreak locality were selected using the iDengue Malaysia portal (https://idengue.mysa.gov.my/) between January 2022 and March 2023. Site selection prioritized areas with > 14 days of dengue cases, high *Aedes* density, and extensive insecticide use, in collaboration with local Health District Officers.

### Mosquito collection

Samples were collected using 100 labelled ovitraps, consisting of 400 ml black containers with a 6.5 cm diameter and 10 cm height, each containing a diagonally placed hardboard paddle measuring 10 × 2.5 × 0.3 cm and 5.5 cm of dechlorinated water [36]. The traps were randomly deployed 5 to 10 m apart near human dwellings in accordance with Ministry of Health guidelines [37], retrieved after 5 to 6 days, and transported to the insectary. Additionally, *Aedes* larvae and pupae were collected from water-holding containers such as flowerpots, tree holes, and old tyres for culturing.

### Mosquito culture and establishment of F1 generations

All immature *Aedes* stages were reared to adults under standard conditions (27 ± 2 ℃, 75 ± 10% RH, 12 h:12 h photoperiod) [38] and fed a larval diet of ground cat biscuit, beef liver powder, milk powder, and yeast (2:1:1:1). Emerging adults were morphologically identified [39] and maintained on 10% sucrose solution ad libitum. Four-day-old females were blood fed on restrained rats (*Rattus norvegicus*) to generate F1 progeny following approval by the USM Institutional Animal Care and Use Committee (USM IACUC; Approval No.: USM/IACUC/2021/(128)(1141). Three days post-feeding, oviposition was induced using moist filter papers placed over water, and F1 eggs were collected for experiments. The susceptible laboratory strain from the Vector Control Research Unit (USM_VCRU) served as the reference.

### World Health Organization (WHO) susceptibility test

#### Larval bioassays

Larval bioassays were conducted following the World Health Organization (WHO) standard protocol [40] using late third- and early fourth-instar F_1_ larvae. Groups of 25 larvae were exposed in 250 mL test solutions containing 6–8 temephos concentrations (0.004–0.042 ppm) prepared in distilled water, with four replicates per concentration. Two control groups containing ethanol (1 mL in 249 mL distilled water) were included. Mortality was recorded after 24 h; larvae unable to swim to the surface were scored as dead, and pupae were excluded. Lethal concentrations (LC_50_, LC_95_, and LC_99.9_) were estimated by probit regression (IBM SPSS Statistics v27.0). Resistance ratios (RR_50_ and RR_95_) were calculated by comparing field strains with the *Ae. albopictus* USM_VCRU susceptible strain to quantify resistance intensity. Diagnostic dose assays were performed using the WHO discriminating concentration (0.012 ppm) and a strain-specific diagnostic dose (0.034 ppm; 2 × LC_99.9_ of the susceptible strain). Susceptibility status was classified according to WHO mortality criteria [40], while RR values were used to compare resistance magnitude among populations rather than for primary classification.

#### Adult bioassays

The adult bioassays were conducted according to WHO protocol [40] using 3–5-day-old non-blood-fed F1 female *Ae. albopictus*. A total of 150 mosquitoes were exposed per insecticide, consisting of 4 replicates of 25 mosquitoes per tubes alongside two replicates for control. The insecticides that were tested are as follows: 0.25% permethrin (Type I pyrethroid), 0.03% deltamethrin (Type II pyrethroid), 0.25% pirimiphos-methyl (organophosphate), and 0.1% propoxur (carbamate) using WHO-standard impregnated papers supplied by Vector Control Research Unit (VCRU), Universiti Sains Malaysia (USM), with replications of assay run for three times. Bioassays were conducted at 28 ± 2 °C and 75 ± 10% relative humidity. For pyrethroids, knockdown was recorded at 5-min intervals during the 1-h exposure period. Knockdown times (KdT_50_ and KdT_95_) were estimated by probit analysis, and knockdown resistance ratios (RR_50_ and RR_95_) were calculated relative to the susceptible *Ae. albopictus* USM_VCRU strain. Mortality was assessed after 24 h according to WHO susceptibility criteria [40]. Both dead and surviving mosquitoes were preserved at − 80 °C for subsequent molecular analyses.

#### Piperonyl butoxide (PBO) synergist bioassays

The effect of the synergist piperonyl butoxide (PBO) was evaluated in populations showing < 98% mortality to pyrethroids. Female F1 mosquitoes (3–5 days old, non-blood fed) were pre-exposed to 4% PBO for 1 h, followed by 0.25% permethrin or 0.03% deltamethrin using WHO susceptibility tests. Mortality was recorded 24 h post-exposure and compared with results from mosquitoes exposed to each insecticide alone. PBO assays were conducted for pyrethroids to assess cytochrome P450-mediated detoxification as part of the targeted investigation of *vgsc*-associated resistance.

### Genotyping of *kdr* mutations in voltage-gated sodium channel (*vgsc*) gene domains

#### Genomic DNA (gDNA) extraction

Genomic DNA was individually extracted from ten female *Ae. albopictus* per locality, comprising five pyrethroid-susceptible and five resistant mosquitoes exposed to each pyrethroid insecticide, as well as from the susceptible USM_VCRU reference strain. Extractions followed a modified Livak protocol [41], with adjusted centrifugation speed and duration. DNA concentration and purity were assessed using a Nanodrop spectrophotometer via A260/A280 and A260/A230, and samples were stored at − 20 °C for downstream analyses.

#### Species identification

For each sample, the species identity of *Ae. albopictus* was confirmed using a PCR-based assay to amplify the ribosomal internal transcribed spacer *ITS1* with species-specific primers, as previously described by Ishak et al. [42].

#### kdr genotyping

Polymorphisms in the partial *vgsc* gene were genotyped in 90 female *Ae. albopictus* using three primer sets targeting DIIS6 (exons 22–23), DIIIS6 (exon 31), and DIVS6 (exon 33), adapted from Ahmad et al. [19] and Kasai et al. [28] (Additional file 1: Table S1). PCRs were performed using 2 × Taq Plus Master Mix II (Vazyme, China) in 25 µL reactions containing 2 mM dNTPs, 6 mM MgCl_2_, 10 µM primers, and 100 ng DNA. Thermocycling conditions were 95 °C for 3 min, 35 cycles of 95 °C for 30 s, 52–55 °C for 30 s, 72 °C for 1 min, and a final extension at 72 °C for 5 min. PCR products for each domain were separated on 1.5% agarose gels pre-stained with RedSafe (INTRON Biotechnology, Korea), purified, and directly sequenced using an Abi 3730xl Big Dye Terminator v3.1 automated sequencer (Applied Biosystems, Foster City, CA) via service from the Apical Scientific (Malaysia). Chromatograms were inspected using Chromas Lite v2.1.1 (Technelysium Pty Ltd, Australia), and all sequences (*n* = 270) were validated by Basic Local Alignment Search Tool (BLAST) and aligned against reference sequences deposited in GenBank. Sequences were assembled, aligned, and trimmed in Molecular Evolutionary Genetics Analysis (MEGA) v11.0 software [43], and both synonymous and non-synonymous mutations, including *kdr* variants, were identified. All sequences were deposited in NCBI GenBank (PQ660263-PQ660378).

#### Statistical analysis

Phenotypic status was determined from 24-h post-exposure mortality following WHO criteria: susceptible (≥ 98%), possibly resistant (90–97%), and resistant (< 90%). Mortality differences among populations were analysed using a bias-reduced binomial generalized linear model (GLM) in R v4.5.1 [44], with Population, Insecticide, and PBO treatment as fixed effects; interaction terms were assessed by Type III ANOVA. Post hoc comparisons of estimated marginal means were conducted using Tukey-adjusted *P*-values. Cross-resistance among adulticides was evaluated using Pearson correlation of mortality rates (*P* < 0.05). Associations between *vgsc* genotypes and pyrethroid resistance were assessed using odds ratios (ORs) with 95% confidence intervals (CI) and Fisher’s exact test, comparing dead (susceptible) and alive (resistant) mosquitoes. Mutations were coded as present/absent at each locus, with alternative substitutions collapsed and zygosity excluded to avoid sparse-data bias. Multi-locus mutation burden was defined as the number of mutated loci per individual, and a Haldane–Anscombe correction [45, 46] was applied where necessary. Analyses were performed using R v4.5.1 and IBM SPSS Statistics v27.0.

### Genetic diversity analysis of *vgsc* gene domains

DNA polymorphism in the *vgsc* gene domains, based on interpopulation variability, was assessed in terms of the number of polymorphic sites, parsimony informative sites, haplotype diversity (Hd), nucleotide diversity (π), the number of segregating sites (S), the average number of nucleotide differences (K), and neutrality tests (Tajima’s D) across the alignment of all 270 sequences (DIIS6, DIIIS6, and DIVS6). These analyses were conducted according to geographical origins using the DnaSP v.5.10.1 software [47].

### Haplotype networks

A haplotype network based on the partial *vgsc* gene domains was constructed using Population Analysis with Reticulate Trees (PopART) (https://popart.maths.otago.ac.nz/) to assess the genealogical relationship of *Ae. albopictus vgsc*-*kdr* haplotypes. Haplotypes were linked from the shortest to the longest distance until all were integrated completely. Population divergence for probabilities was analysed using TCS networks inference [48] constructed within the PopART software.

### Homology modelling of partial *vgsc* gene domain’s protein

The 3D protein structure of partial *vgsc* DIIS6, DIIIS6, and DIVS6 proteins were constructed by ColabFold v1.5.5: AlphaFold2 using MMseqs2 [49] pipeline and default AlphaFold parameters. Five models per domain were evaluated via PROCHECK (UCLA-DOE LAB SAVES v6.1) using Ramachandran plots and pLDDT scores > 70. Models with the highest residues in allowed regions and minimal disallowed residues were selected for further analyses (Additional file 2: Fig. S1).

### Docking

3D conformers of deltamethrin and permethrin were retrieved from PubChem (https://pubchem.ncbi.nlm.nih.gov/docs/compounds) and energy minimized using the MMFF94 force field in Open Babel. Docking simulations were performed with AutoDock Vina in PyRx 0.8 [50], recording binding affinities and lowest-RMSD poses. Differences between wild-type and mutant proteins were assessed by aligning and visualizing the best-docked poses in UCSF ChimeraX [51]. Analysis of protein–ligand interaction was performed using Protein–Ligand Interaction Profiler (PLIP) [52], based on the docking results.

## Results

### Susceptibility profiling to insecticides

#### Susceptibility status of larval *Ae. albopictus*

*Aedes albopictus* larvae exposed to 0.012 and 0.034 ppm temephos exhibited low mortality across all field populations (5.7–59.0%; Additional file 3: Table S2, Fig. S2), with slightly higher resistance ratios observed in the KB strain from Perlis (Additional file 3: Table S3). Bias-reduced binomial GLM analysis confirmed USM_VCRU as the most susceptible population (Firth’s penalized likelihood test, OR = 401, 95% CI 24.8–6477.9, *P* < 0.001), whereas SA (OR = 0.0017, 95% CI 0.00010–0.028, *P* < 0.001), BP (OR = 0.0036, 95% CI 0.00022–0.059, *P* < 0.001), KB (OR = 0.00015, 95% CI 9.1 × 10^–6^–0.0026, *P* < 0.001), and KTT (OR = 0.00026, 95% CI 1.57 × 10^–5^–0.0043, *P* < 0.001) were significantly more resistant. Type III ANOVA on the GLM revealed significant effects of population (*χ*^2^ = 684.86, *df* = 4, *P* < 0.0001), insecticide concentration (*χ*^2^ = 534.99, *df* = 1, *P* < 0.0001), and their interaction (*χ*^2^ = 110.07, *df* = 4, *P* < 0.0001), indicating population-specific differences in temephos susceptibility. Post hoc comparisons showed USM_VCRU mortality was higher than all field populations at 0.012 ppm (*P* < 0.001), whereas at 0.034 ppm, differences among resistant populations were reduced (SA vs KB, *P* = 0.808), although KB and KTT remained highly resistant (*P* < 0.001). Overall, KB and KTT exhibited the strongest resistance, and increasing temephos concentration only partially mitigated mortality differences*.*

#### Susceptibility status of adults Ae. albopictus

*Mortality rates and cross-resistance correlation*. All field populations of *Ae. albopictus* from Penang and Perlis exhibited varying resistance to carbamates, organophosphates, and pyrethroids (Additional file 4: Table S4). Adult mortality differed significantly among populations (bias-reduced binomial GLM; Type III likelihood-ratio *χ*^2^ = 1878.80, *df* = 4, *P* < 0.0001) and insecticides (*χ*^2^ = 1672.28, *df* = 3, *P* < 0.0001), with a significant Population × Insecticide interaction (*χ*^2^ = 314.76, *df* = 12, *P* < 0.0001), indicating population-specific variation in insecticide response. Compared with the susceptible USM_VCRU strain, mortality was markedly reduced in all field populations: SA (Firth’s penalized likelihood test, OR = 0.0062, 95% CI 0.00038–0.10, *P* < 0.001), BP (OR = 0.0216, 95% CI 0.0013–0.36, *P* = 0.0076), KB (OR = 0.00086, 95% CI 0.00005–0.014, *P* < 0.001), and KTT (OR = 0.0016, 95% CI 0.00010–0.026, *P* < 0.001), consistent with strong resistance across populations. Post hoc comparisons confirmed significantly lower mortality in all field populations for most insecticides (*P* < 0.05), with BP showing particularly low mortality to propoxur (OR = 0.0117, 95% CI 0.0002–0.622, *P* = 0.028). Propoxur resistance was universal, with KB exhibiting the lowest mortality (2.0%), whereas resistance to pirimiphos-methyl was observed in all populations (KTT: 70.0%; BP: 92.3%). Pyrethroid assays revealed resistance in Penang populations (permethrin: SA 79.0%, BP 93.0%; deltamethrin: SA 96.7%, BP 90.0%) and lower susceptibility in Perlis (permethrin: KB 34.0%, KTT 48.7%; deltamethrin: KB 3.7%, KTT 10.5%). Exploratory correlation analyses suggested a positive, non-significant trend towards cross-resistance between permethrin and deltamethrin (*r* = 0.950, *P* = 0.050), with no significant associations between other insecticide pairs.

*Knockdown effects*. Knockdown times for 50% (KdT_50_) and 95% (KdT_95_) of *Ae. albopictus* were determined for pyrethroid exposure, revealing substantial heterogeneity among field populations (KdT_50_: 15.57–199.45 min; KdT_95_: 28.22–378.38 min) (Additional file 4: Table S5). Deltamethrin was most effective in Penang populations (SA: KdT_50_ = 15.57 min; BP: 16.86 min), whereas permethrin was less effective. In contrast, Perlis populations (KB, KTT) exhibited very low knockdown to deltamethrin (< 5%), precluding KdT estimation and indicating pronounced resistance. Permethrin elicited measurable knockdown in Perlis populations, but overall susceptibility was higher in Penang than in Perlis. Distinct non-overlapping confidence intervals among populations confirmed significant variation in knockdown responses. KdT_50_ and KdT_95_ were used to quantify resistance intensity and compare populations, while susceptibility was determined independently based on WHO mortality criteria.

**Fig. 2:**
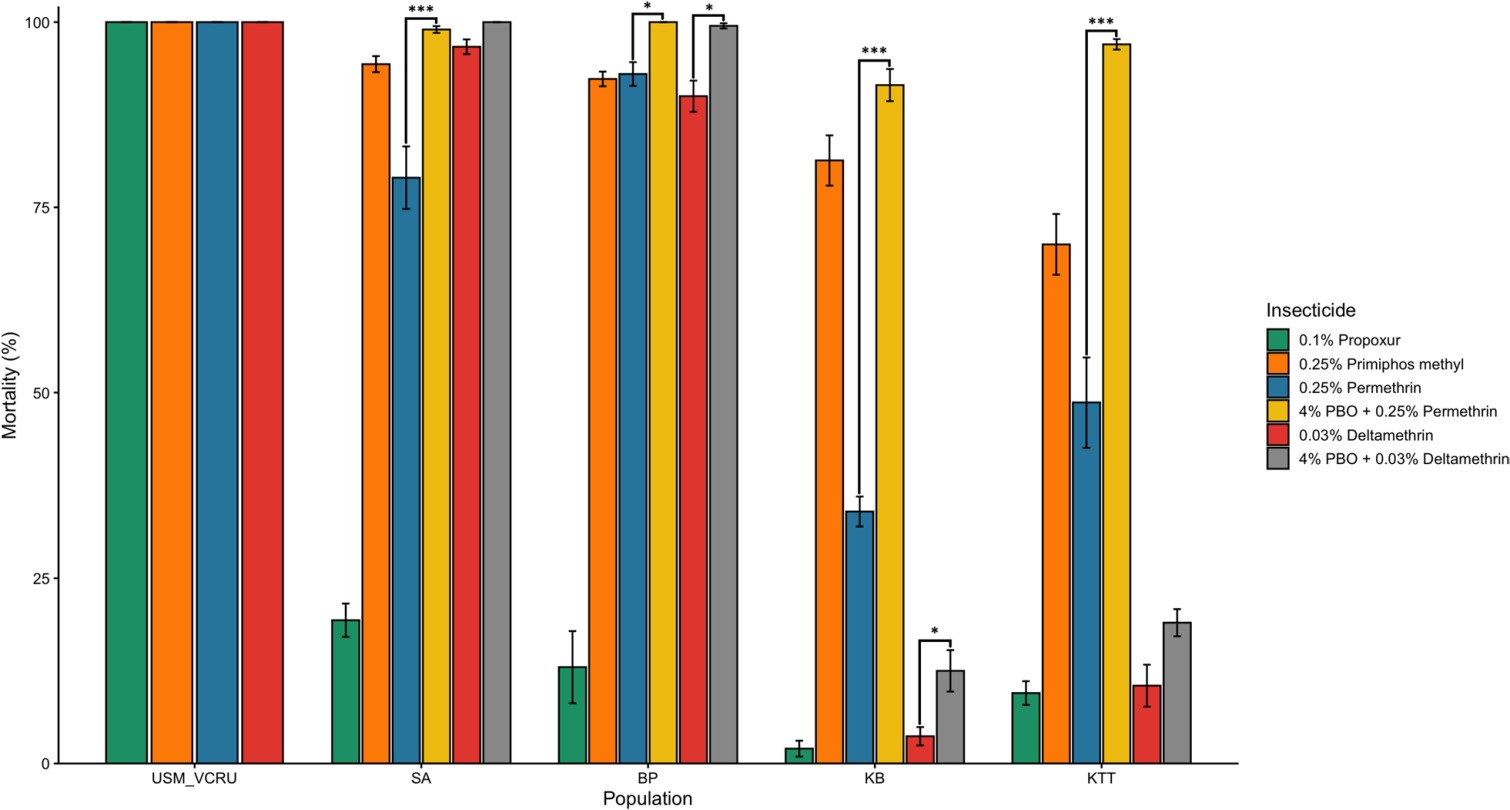
Percentage mortality of female *Ae. albopictus* from four different localities in Penang and Perlis, Malaysia towards three classes of insecticides and a PBO-synergist. Bars represent mean mortality values, and error bars denote standard deviation (± SD) based on replicates. Mortality differences for the pyrethroid insecticides tested (0.25% permethrin and 0.03% deltamethrin), together with their respective PBO-synergized treatments, were analysed using a bias-reduced binomial generalized linear model (GLM). Statistical significance shown in the figure refers specifically to the effect of PBO pre-treatment. Asterisks on top of the bars indicate statistical significance, where * = *P* < 0.05 and *** = *P* < 0.001 when mosquitoes were pre-treated with PBO, indicating restoration of susceptibility to the insecticides

*Synergism assay with PBO.* Pre-exposure to 4% PBO for 1 h prior to pyrethroid exposure increased mortality across all *Ae. albopictus* populations. In Penang, susceptibility was largely restored, with 99.0% (SA) and 99.5% (BP) mortality following PBO and permethrin, and complete recovery with deltamethrin. Perlis populations exhibited strong synergism with permethrin (KB: 34.0 to 91.5%; KTT: 48.7 to 97.0%) but minimal restoration with deltamethrin (KB: 3.7 to 12.5%; KTT: 10.5 to 19.0%). Bias-reduced binomial GLM confirmed that PBO significantly increased mortality overall (*P* < 0.05), with no significant Population × PBO Interaction, indicating generally comparable recovery among populations. Post hoc analysis showed significant enhancement of permethrin-induced mortality in all populations (*P* < 0.05; strongest in SA, KB, and KTT, *P* < 0.001), whereas deltamethrin mortality increased significantly only in BP and KB (Additional file 4: Table S4 and Fig. 2).

### Genotyping of *kdr *mutations of *Ae. albopictus* across Northern Peninsular Malaysia

#### Detection of kdr mutations in the vgsc gene domains of Ae. albopictus

**Fig. 3:**
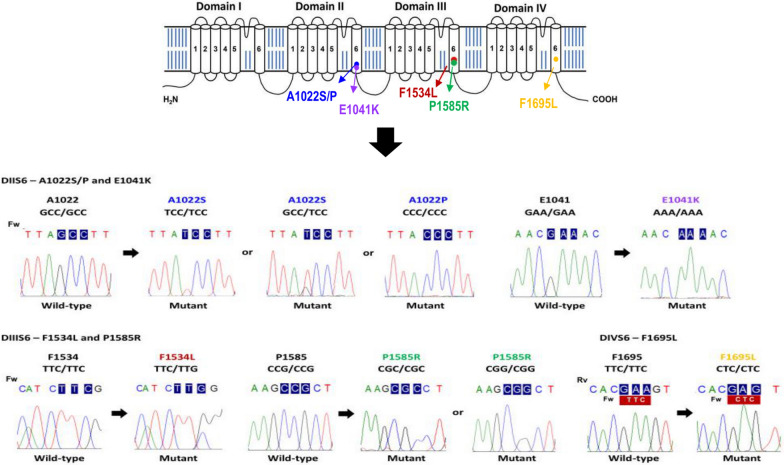
Chromatogram of the wild-type and mutated alleles found in this study. Schematics diagram of voltage-gated sodium channel (*vgsc*) with the occurrence of several mutations in the studied *Ae. albopictus* illustrated by chromatogram of sequence data, displaying both wild-type and mutated alleles that could affect the binding of pyrethroid insecticides to the *vgsc* target site

**Fig. 4:**
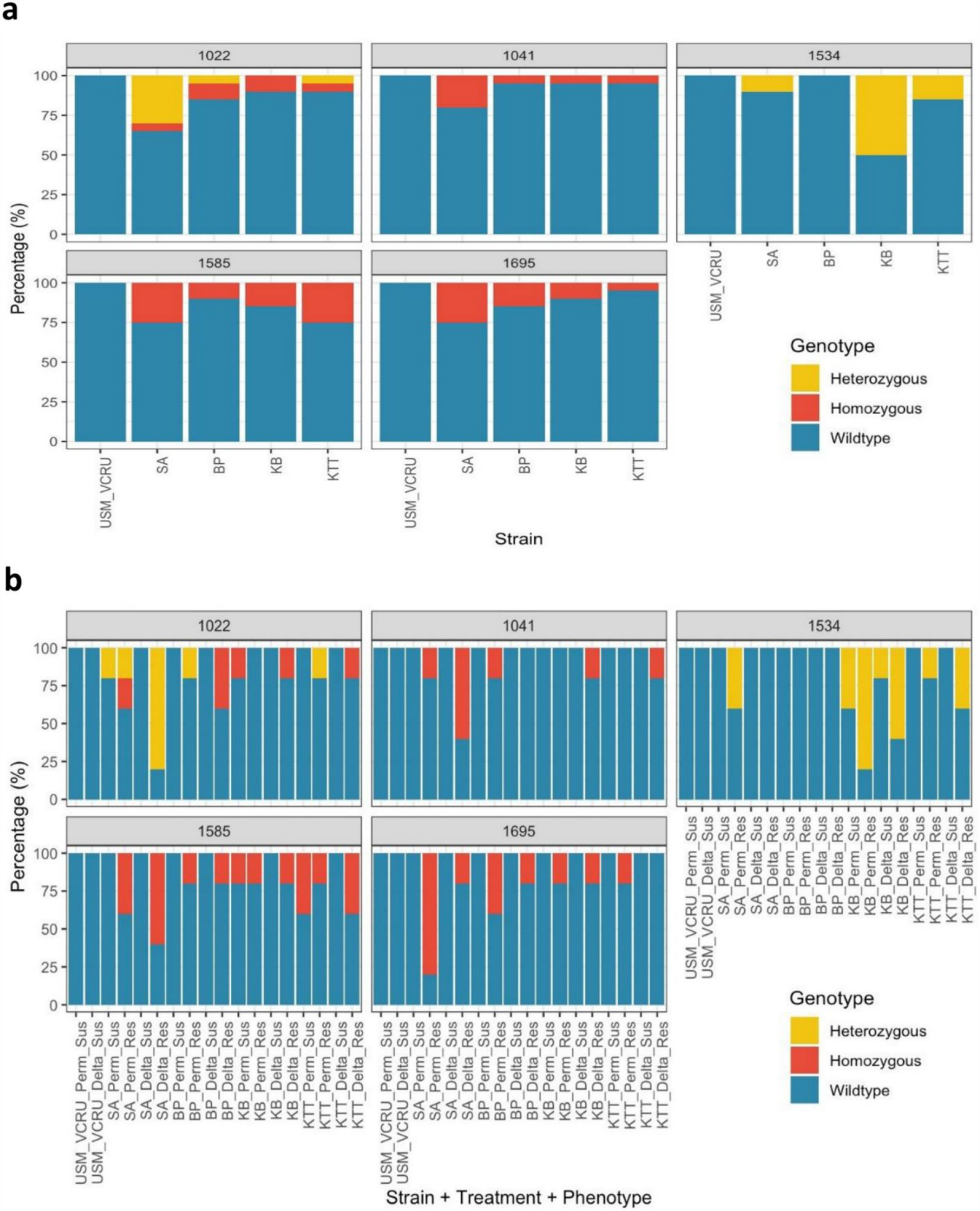
The percentage distributions of different genotypes (wild type, heterozygous, and homozygous) in *Ae. albopictus* from USM_VCRU, SA, BP, KB, and KTT are shown at *vgsc* loci 1022, 1041, 1534, 1585, and 1695. **a** Genotype distribution by strain; **b** Genotype distribution by strain, treatment, and phenotype

Ninety *Ae. albopictus* samples from pyrethroid-resistant and -susceptible field strains were screened for known *kdr* mutations at codons 989, 1011, 1016, 1520, 1532, 1534, and 1763 across DIIS6, DIIIS6, and DIVS6, and the results are tabulated (Additional file 5: Table S6). Only F1534L was detected in both Penang and Perlis populations, with higher heterozygous prevalence in Perlis (32.5%, 13/40) than in Penang (5%, 2/40), primarily in KB strains. Multiple novel non-synonymous mutations were identified, mostly in resistant mosquitoes. In DIIS6, A1022S occurred heterozygously in Penang (17.5%) and homozygously in Perlis (7.5%), while A1022P and E1044K were restricted to Penang resistant strains. In DIIIS6, P1585R was prevalent in resistant SA (50%) and distributed in KB and KTT (20%). In DIVS6, no *kdr* 1763 alleles were detected, but L/CTC was observed in resistant mosquitoes (SA: 50%; BP: 30%). Genotypic prevalence of alleles in DIIS6, DIIIS6, and DIVS6 is shown in Fig. 3, and locus-specific genotypic distributions in Penang and Perlis are summarized in Fig. 4. Overall, mutant alleles accounted for 13.8% of total detections in Northern Peninsular Malaysia (Additional file 5: Table S7). Notably, a proportion of susceptible individuals also carried *vgsc* mutations, with mutation frequencies of 4.44% (A1022), 0% (E1041), 6.67% (F1534), 6.98% (P1585), and 0% (F1695) (Additional file 5: Table S7), indicating incomplete concordance between genotype and phenotype.

#### Distribution of single-, dual-, triple-, and quadruple-loci of the genotypic combination in DIIS6, DIIIS6, and DIVS6 of vgsc gene in Ae. albopictus

**Fig. 5:**
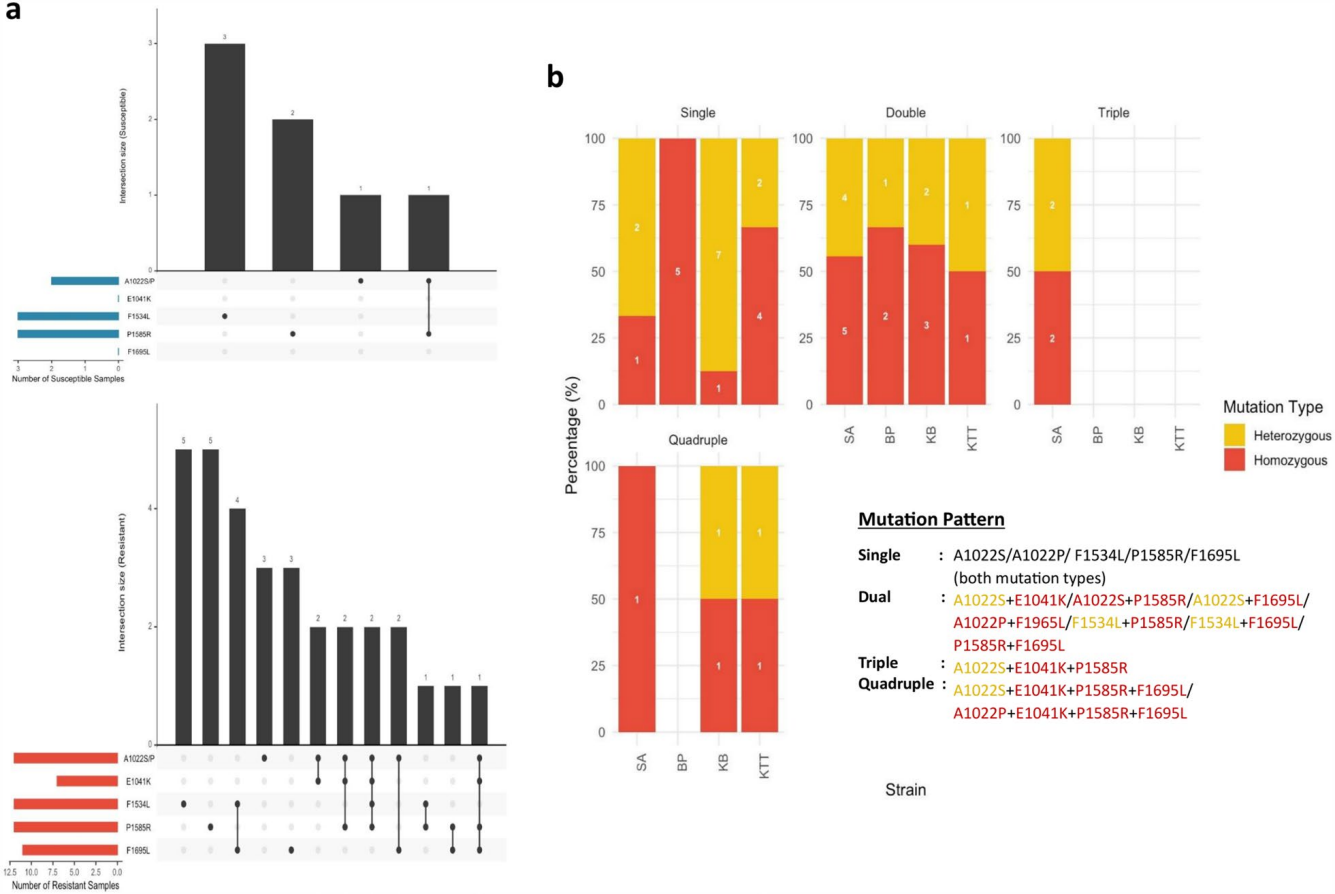
Analysis of mutation patterns and frequencies. **a** The Upset plot shows the intersection sizes of different mutation patterns and the percentage of samples carrying specific mutation types (single, double, triple, quadruple) at loci 1022, 1041, 1534, 1585, and 1695. Vertical bars indicate the number of samples containing particular mutation combinations, while the connected dots below each bar represent the specific mutations present in each intersection. **b** The histogram shows the percentage distribution of single, double, triple, and quadruple mutation types across different strains, highlighting which mutation types are more prevalent. Specific mutation patterns observed within each strain are also indicated

The distribution of *kdr* F1534L and novel non-synonymous mutations (A1022S/P, E1041K, P1585R, F1695L) with pyrethroid resistance were examined by genotyping 90 resistant and susceptible *Ae. albopictus* across DIIS6, DIIIS6, and DIVS6. Among 80 field-strain mosquitoes from Northern Peninsular Malaysia, 16 distinct substitution patterns were identified: 5 single-locus (27.5%), 7 dual-loci (13.8%), 2 triple-loci (2.5%), and 3 quadruple-loci (3.8%) combinations (Additional file 5: Table S8). Single mutations predominated in resistant (17.8%) and susceptible (6.7%) mosquitoes, with F1534L detected in 8 resistant Perlis samples. Dual- and multi-locus mutations, often involving E1041K co-occurrences, were mostly found in resistant mosquitoes. Triple and quadruple combinations, including A1022P/S + E1041K + P1585R ± F1695L, were restricted to resistant samples. Susceptible mosquitoes primarily carried single mutations, whereas resistant populations exhibited higher prevalence of multiple concurrent mutations (Fig. 5), indicating a potential cumulative contribution to pyrethroid resistance.

#### The association between vgsc mutation burden in DIIS6, DIIIS6, and DIVS6 segments and pyrethroid resistance across Ae. albopictus populations

The association between *vgsc* mutations and pyrethroid resistance in the studied *Ae. albopictus* is summarized in Table 1. Mutation burden is used to reference the presence of either single or multiple mutations present. The susceptible reference strain (USM_VCRU) exclusively of mosquitoes without *vgsc* mutations and showed no resistant individuals, providing a baseline for genotype–phenotype comparisons. Across field populations, mosquitoes lacking *vgsc* mutations were predominantly susceptible, whereas the presence of mutations was associated with increased resistance.

**Table 1:** Multi-locus *vgsc* mutation categories and their association with pyrethroid resistance in *Ae. albopictus* from Penang (SA and BP) and Perlis (KB and KTT)

Genotype category	Mutation combination	USM VCRU Sus	SA Sus	SA Res	BP Sus	BP Res	KB Sus	KB Res	KTT Sus	KTT Res	Total Sus	Total Res	OR	*P*-value
WT only	None	10	9	0	10	3	6	2	8	4	43	9	Ref	–
Single mutation	All single mutants	0	1	2	0	5	3	5	2	4	6	16	4.7979	0.0029^**^
	A1022P	0	0	0	0	1	0	0	0	0	0	1	∞	0.2057
	A1022S	0	1	1	0	0	0	0	0	1	1	2	2.5523	0.5829
	F1534L	0	0	0	0	0	3	4	0	1	3	5	2.2180	0.4584
	P1585R	0	0	1	0	2	0	0	2	2	2	5	3.3827	0.2345
	F1695L	0	0	0	0	2	0	1	0	0	0	3	∞	0.0401
Double mutations	All duo-loci mutants	0	0	5	0	1	1	2	0	1	1	9	15.9013	0.0020^**^
	A1022P + F1695L	0	0	0	0	1	0	0	0	0	0	1	∞	0.2057
	A1022S + E1041K	0	0	1	0	1	0	0	0	0	0	2	∞	0.1948
	A1022S + F1695L	0	0	1	0	0	0	0	0	0	0	1	∞	0.2057
	A1022S + P1585R	0	0	0	0	0	1	0	0	0	1	0	0.0000	0.5031
	F1534L + P1585R	0	0	0	0	0	0	1	0	0	0	1	∞	0.2057
	F1534L + F1695L	0	0	3	0	0	0	1	0	1	0	5	∞	0.0074^**^
Triple mutations	All triple-loci mutants	0	0	2	0	0	0	0	0	0	0	2	∞	0.1948
	A1022S + E1041K + P1585R	0	0	2	0	0	0	0	0	0	0	2	∞	0.1948
Quadruple mutations	All quadruple-loci mutants	0	0	1	0	0	0	1	0	1	0	3	∞	0.0401^*^
	A1022P + E1041K + P1585R + F1695L	0	0	1	0	0	0	0	0	0	0	1	∞	0.2057
	A1022S + E1041K + F1534L + P1585R	0	0	0	0	0	0	1	0	1	0	2	∞	0.1948

Ref: Reference group. (–): NA. Range of odds ratio (OR): OR = 1 (no association between genotype and phenotype), OR < 1 (low likelihood of insecticide resistance), OR > 1 (high likelihood of insecticide resistance), OR = ∞ reflects an unbounded odds ratio due to zero susceptible individuals observed in the corresponding genotype category; a 0.5 continuity (Haldane–Anscombe) correction was applied (higher likelihood of insecticide resistance). Statistical significance: Fisher’s exact test was used; *P* < 0.05 indicates statistical significance (*P* < 0.01, **; *P* < 0.05, *). Odds ratios (OR) and Fisher’s exact tests were calculated using WT-only mosquitoes as the reference group. Individual mutation combinations within double, triple, and quadruple categories are shown descriptively due to low sample sizes. Mutations were coded as binary presence/absence regardless of zygosity

When all single-mutation genotypes were pooled, a significant association with resistance was detected (Fisher’s exact test, *P* = 0.0029, OR = 4.80, 95% CI 1.53–17.05), although individual loci showed heterogeneous effects. As the analysis was conducted at the individual mutation level, subgroup sizes were small, which may limit statistical power and should be considered when interpreting these estimates. Only F1695L exhibited a significant association (*P* = 0.0401, OR = ∞, 95% CI 0.81-∞), while other single mutations showed elevated but non-significant odds. In contrast, multi-locus genotypes displayed substantially stronger associations with resistance. Pooled double-mutation genotypes were strongly associated with resistance (*P* = 0.0020, OR = 15.90, 95% CI 2.07–720.19), and the F1534L + F1695L combination was detected exclusively among resistant mosquitoes. Triple- and quadruple-mutation genotypes were rare but occurred predominantly in resistant individuals, with quadruple-mutation genotypes showing a significant association (*P* = 0.0401, OR = ∞, 95% CI 0.81–∞). Overall, resistance increased stepwise with mutation accumulation, indicating a descriptive dose-dependent pattern of multi-locus *vgsc* mutations. Individual mosquito-level odds ratio and mutation-specific odd ratio estimates showed substantial instability and provided weak interpretation of resistance risk due to low mutation frequencies and sparse contingency tables (Additional file 6: Tables S9, S10). Accordingly, these odds ratios are presented as supplementary descriptive analyses. The pooled multi-locus and mutation burden odds ratios are emphasized as they provide more robust and biologically interpretable estimates of resistance risk. Detailed zygosity distributions are provided in Additional file 7: Tables S11, S12.

#### Insecticide-specific association between vgsc mutation burden and pyrethroid resistance

Stratified analysis demonstrated a strong association between *vgsc* mutation burden and pyrethroid resistance after adjustment for insecticide exposure (Mantel–Haenszel Chi-square test, *χ*^2^ = 15.16, *P* < 0.001; common OR = 24.47, 95% CI 4.00–149.55). Based on Table 2, single-mutation genotypes were not significantly associated with resistance (*P* = 0.33, OR = 2.18, 95% CI 0.52–9.76) under permethrin selection, whereas double-mutation genotypes showed a significant increase in resistance risk (*P* = 0.029, OR = 6.58, 95% CI 1.10–72.32). Higher-order mutation categories were rare and did not yield statistically robust estimates. In contrast, deltamethrin resistance was evident at lower mutation burdens, with single-mutation genotypes significantly associated with resistance (*P* = 0.012, OR = 7.62, 95% CI 1.26–84.83) and stronger effects observed for double mutations (*P* = 0.038, OR = ∞, 95% CI 0.83–∞). All mosquitoes carrying higher mutation burdens under deltamethrin exposure were resistant, indicating a clear insecticide-specific, dose-dependent effect of *vgsc* mutation accumulation.

**Table 2:** Insecticide-specific association of multi-locus *vgsc* mutation burden with pyrethroid resistance in *Ae. albopictus*, showing odds ratios and Fisher’s exact test *P*-values for 0.25% permethrin and 0.03% deltamethrin

Insecticide	Mutation	Mutation counts	OR	*P*-value
0.25% Permethrin	Single	13	2.1827	0.3330
	Double	8	6.5812	0.0293
	Triple	0	NA	NA
	Quadruple	1	∞	0.2048
0.03% Deltamethrin	Single	9	7.6230	0.0125
	Double	3	∞	0.0376
	Triple	2	∞	0.2474
	Quadruple	2	∞	0.2474

‘NA’ indicates ORs could not be estimated because of zero cell counts. Range of odds ratio (OR): OR = 1 (no association between genotype and phenotype), OR < 1 (low likelihood of insecticide resistance), OR > 1 (high likelihood of insecticide resistance), OR = ∞ indicates no susceptible mosquitoes were observed within a mutation category; a 0.5 continuity (Haldane–Anscombe) correction was applied (higher likelihood of insecticide resistance). Statistical significance was defined as *P* < 0.05

### Haplotype distributions and polymorphism analysis of the partial *vgsc* gene domains (DIIS6, DIIIS6, and DIVS6) in Northern Peninsular Malaysian of *Ae. albopictus*

#### kdr-vgsc haplotypes

**Fig. 6:**
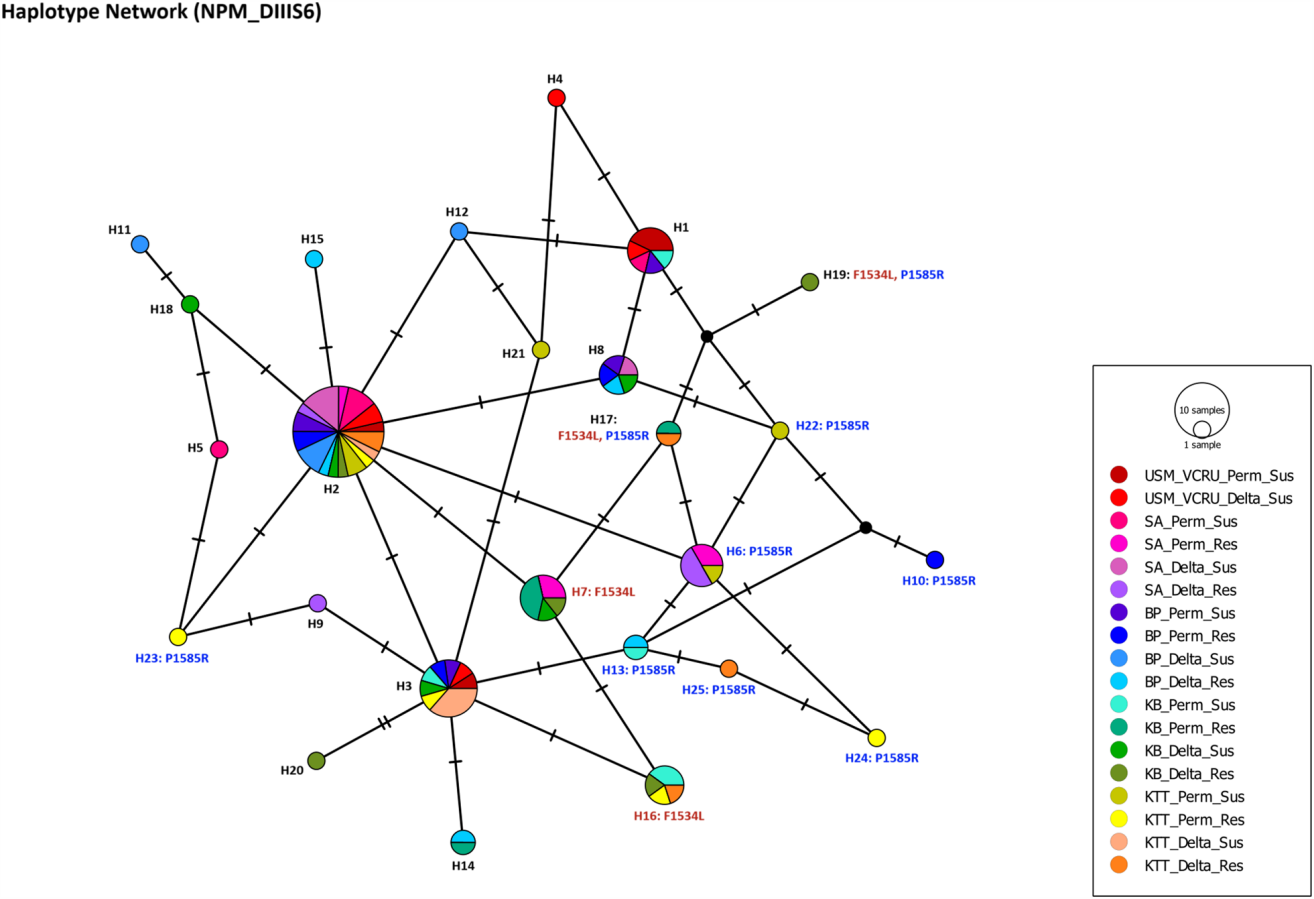
Haplotype network showing the genealogical relationships of 25 haplotypes in DIIIS6 based on four strains of Northern Peninsular Malaysia populations of *Ae. albopictus*: wild-type haplotypes are depicted in black, haplotypes with *kdr* mutations associated with resistance phenotypes are marked in red, and those with new non-synonymous mutations are highlighted in blue

Genealogical network analysis of 90 *Ae. albopictus* revealed 41, 25, and 50 haplotypes based on 326 bp, 221 bp, and 283 bp of DIIS6, DIIIS6, and DIVS6, respectively, all deposited in NCBI GenBank (Additional file 8: Table S13). In DIIS6, 45 polymorphic sites produced 41 haplotypes (Additional file 8: Fig. S3), with H3 (16.7%) representing the ancestral wild-type genotype V1016/A1022/E1041, dominant in USM_VCRU, suggesting pre-insecticide prevalence. Seven haplotypes carried A1022S/P and six carried A1022S/P + E1041K, distributed in both Penang and Perlis. In DIIIS6, *kdr*-F1534L (H7) and P1585R (H17/H19) arose via independent mutational events, primarily in Perlis, while other unique haplotypes represent resistant mosquitoes (Fig. 6). DIVS6 lacked D1763 *kdr* haplotypes but exhibited 39 unique haplotypes from common ancestors (Additional file 8: Fig. S4), reflecting selection-driven diversity. Shared haplotypes indicate gene flow between populations, with clustering observed across susceptible and resistant alleles.

#### Genetic diversity and neutrality test

Alignment of 90 *Ae. albopictus* across partial *vgsc* domains (DIIS6, DIIIS6, DIVS6) revealed domain-specific genetic diversity and neutrality indices (Table 3). Nucleotide diversity (π) was moderate for DIIS6 (0.01201) and DIVS6 (0.01008) and lower for DIIIS6 (0.00823), with the highest haplotype diversity in DIVS6 (Hd = 0.961). Neutrality tests indicated evidence of adaptive evolution in DIIS6 (Tajima’s D = − 1.922, *P* < 0.01; Fu’s *Fs* = − 29.893), whereas DIIIS6 and DIVS6 did not significantly depart from neutrality (DIIIS6: D = − 0.946, Fu’s *Fs* = − 20.363; DIVS6: D = − 1.272, Fu’s *Fs* = − 33.646), consistent with recent population expansion or purifying selection [53, 54].

**Table 3:** Genetic diversity, neutrality tests, and mutations in the partial *vgsc* gene domains between pyrethroids susceptible and resistant *Ae. albopictus* across Northern Peninsular Malaysia (Penang and Perlis)

		Genetic diversity analysis							Neutrality test		Mutations (n)	
	Samples/Phenotypes	n	#	S	h	Hd (SD)	π	k	D	*Fs*	Syn	Nsyn
	VCRU Susceptible strain											
	VCRU_Sus	10	1	5	3	0.644 (0.101)	0.00409	1.33333	− 0.98485	1.056	5	0
	Penang strains											
	SA_Sus	10	10	11	8	0.956 (0.056)	0.01404	4.57778	0.79395	− 2.234	10	1
	SA_Res	10	13	26	10	1.000 (0.045)	0.02713	8.84444	− 0.65791	− 3.850	13	16
	BP_Sus	10	3	8	6	0.778 (0.137)	0.00688	2.24444	− 0.89031	− 1.533	7	1
	BP_Res	10	5	19	10	1.000 (0.045)	0.01541	5.02222	− 1.36143	− 5.898	7	13
DIIS6	Overall	40	19	31	28	0.968 (0.016)	0.01668	5.43718	− 1.24811	− 18.143	19	17
	Perlis strains											
	KB_Sus	10	4	16	8	0.933 (0.077)	0.01391	4.5333	− 1.14206	− 2.262	13	4
	KB_Res	10	2	12	4	0.733 (0.101)	0.00886	2.88889	− 1.44062	1.454	7	5
	KTT_Sus	10	6	12	5	0.756 (0.077)	0.01145	3.73333	− 0.54145	0.837	12	0
	KTT_Res	10	3	15	7	0.911 (0.077)	0.01063	3.46667	− 1.59457	− 1.657	10	5
	Overall	40	16	31	17	0.917 (0.023)	0.01201	3.91667	− 1.77277	− 5.288	24	10
	Northern Peninsular Malaysia strains											
	Overall	90	32	45	41	0.939 (0.014)	0.01201	4.48639	− 1.92183^*^	− 29.893	36	20
	VCRU Susceptible strain											
	VCRU_Sus	10	3	3	4	0.778 (0.091)	0.00714	1.57778	1.75514	0.078	3	0
	Penang strains											
	SA_Sus	10	1	4	4	0.533 (0.180)	0.00432	0.95556	− 1.24468	− 0.971	4	0
	SA_Res	10	2	4	4	0.733 (0.120)	0.00593	1.31111	− 0.27911	− 0.312	2	2
	BP_Sus	10	2	5	6	0.778 (0.137)	0.00593	1.31111	− 1.03527	− 3.023	5	0
	BP_Res	10	3	6	7	0.911 (0.077)	0.00885	1.95556	− 0.32254	− 3.347	5	1
DIIIS6	Overall	40	7	10	14	0.801 (0.058)	0.00673	1.48718	− 1.28942	− 9.284	9	2
	Perlis strains											
	KB_Sus	10	3	6	8	0.956 (0.059)	0.00895	1.97778	− 0.27920	− 5.126	4	2
	KB_Res	10	3	8	7	0.867 (0.107)	0.01036	2.28889	− 0.82232	− 2.845	6	2
	KTT_Sus	10	2	4	5	0.800 (0.100)	0.00593	1.31111	− 0.27922	− 1.587	3	1
	KTT_Res	10	4	5	7	0.911 (0.077)	0.00915	2.02222	0.57806	− 3.238	3	2
	Overall	40	6	11	18	0.910 (0.025)	0.00884	1.95385	− 0.74185	− 13.465	9	2
	Northern Peninsular Malaysia strains											
	Overall	90	9	13	25	0.872 (0.026)	0.00823	1.81898	− 0.94611	− 20.363	12	2
	VCRU Susceptible strain											
	VCRU_Sus	10	4	5	5	0.822 (0.097)	0.00683	1.93333	0.37639	− 0.680	5	0
	Penang strains											
	SA_Sus	10	3	8	8	0.933 (0.077)	0.00793	2.24444	− 0.89013	− 4.619	8	0
	SA_Res	10	3	8	8	0.956 (0.059)	0.00927	2.62222	− 0.31377	− 4.032	7	1
	BP_Sus	10	3	8	7	0.911 (0.077)	0.00864	2.44444	− 0.58500	− 2.644	8	0
	BP_Res	10	6	10	10	1.000 (0.045)	0.01264	3.57778	0.05382	− 7.424	9	1
DIVS6	Overall	40	11	18	27	0.946 (0.027)	0.01022	2.89231	− 1.03377	− 26.975	17	1
	Perlis strains											
	KB_Sus	10	6	7	7	0.933 (0.062)	0.00927	2.62222	0.25304	− 2.435	7	0
	KB_Res	10	7	9	10	1.000 (0.045)	0.01084	3.06667	− 0.15788	− 8.194	8	1
	KTT_Sus	10	4	9	8	0.956 (0.059)	0.01005	2.84444	− 0.46374	− 3.741	9	0
	KTT_Res	10	3	9	9	0.978 (0.054)	0.00989	2.80000	− 0.92145	− 5.830	8	1
	Overall	40	12	17	29	0.978 (0.012)	0.01070	3.02949	− 0.92790	− 31.227	16	1
	Northern Peninsular Malaysia strains											
	Overall	90	18	24	50	0.961 (0.011)	0.01008	2.85194	− 1.27244	− 33.646	23	1

*n*: sample size; #: no of parsimony informative sites; S: number of variable sites; h: number of haplotypes per locality; Hd: haplotype diversity; π: nucleotide diversity; K: average number of nucleotide differences; D: Tajima’s *D*; *Fs:* Fu’s *Fs* statistic. *Statistical significance (D test): *P* < 0.01; Syn: synonymous mutation; Nsyn: Non-synonymous mutations; level of genetic diversity: very low π (0–0.0001), low π (0.001–0.01), moderate π (0.01–0.05), high π (0.05–0.1 and above) (Nei, 1987; Kartavtsev, 2011)

### Protein prediction structures and docking of permethrin and deltamethrin to the partial DIIS6, DIIIS6, and DIVS6 *vgsc* gene of *Ae. albopictus*

#### Protein prediction structures

Homology models of the partial *vgsc* protein domains DIIS6, DIIIS6, and DIVS6 were generated for wild-type, mutant, and combined alignments, highlighting the mutations identified in this study (A1022S/P, E1041K, F1534L, P1585R, and F1695L; Additional file 9: Fig. S5). Ligand interactions with permethrin and deltamethrin were analysed for each domain, and positional differences at mutation sites are shown in Additional file 9: Fig. S6, illustrating altered binding relative to the wild type.

#### Protein–ligand interactions

**Fig. 7:**
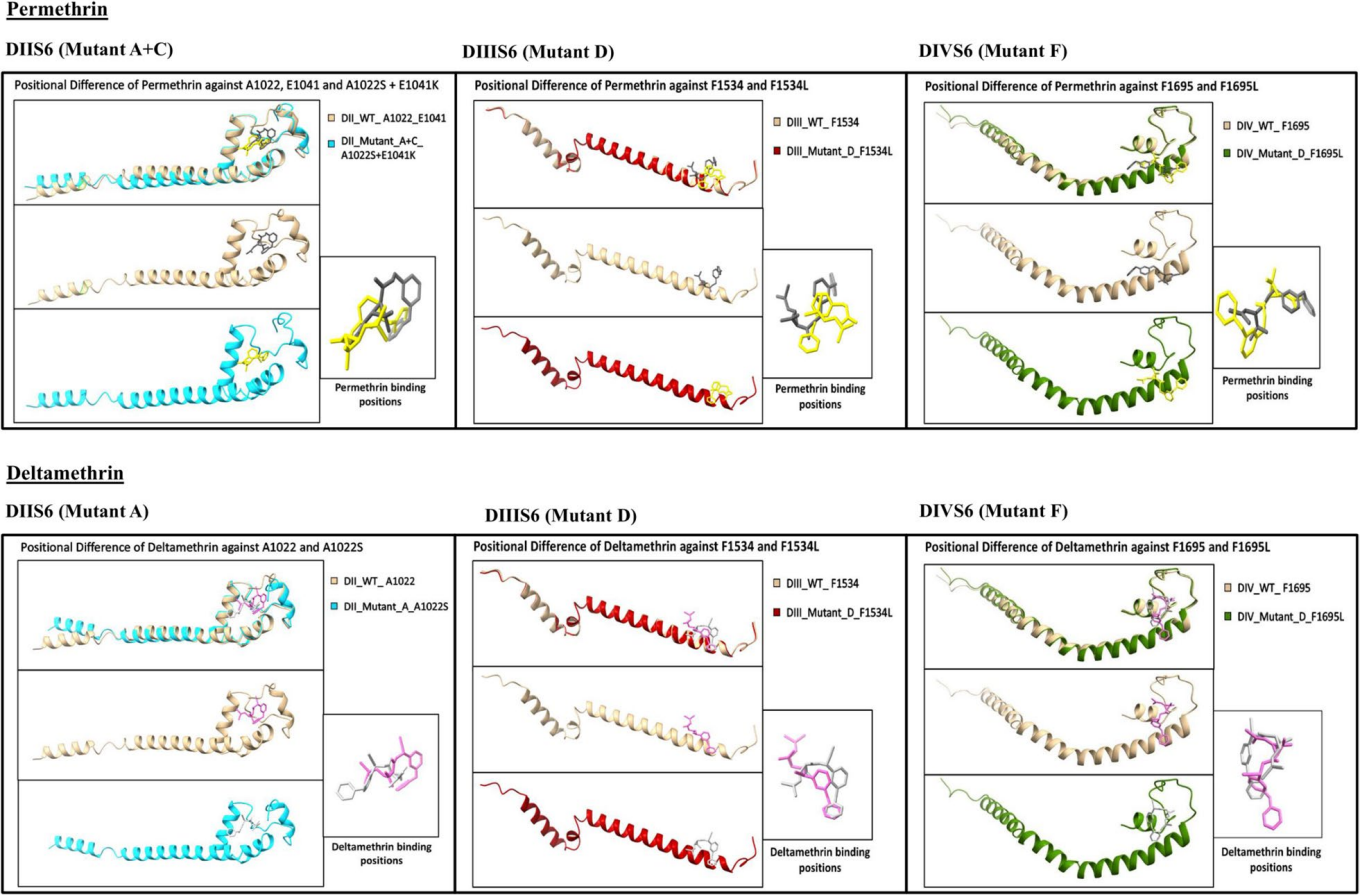
Positional difference binding of permethrin and deltamethrin against the wild-type and mutant variants in DIIS6, DIIIS6, and DIVS6 with the lowest-affinity interactions

In silico docking of permethrin and deltamethrin with partial *vgsc* domains revealed distinct binding affinities (Table 4), with predicted structures of lowest-affinity interactions, and changes in residue interactions are shown in Fig. 7. Lower binding scores indicate stronger affinity. In DIIS6, mutations generally reduced ligand binding, with A1022S showing the largest decrease for deltamethrin (− 7.8 to − 6.0 kcal/mol) and E1041K and combined mutants (A1022S + E1041K, A1022P + E1041K) also exhibiting notable reductions (− 7.3 to − 6.5 kcal/mol). For DIIIS6, F1534L and F1534L + P1585R decreased affinity (− 6.3 to − 5.0 kcal/mol), whereas P1585R alone had minimal effect. Similar patterns were observed for permethrin, with E1041K and A1022S + E1041K showing the greatest reductions, while DIVS6 F1695L did not alter binding for either insecticide. These results indicate that DIIS6 and DIIIS6 mutations variably impair pyrethroid binding, whereas DIVS6 remains unaffected.

**Table 4:** Molecular docking interactions of permethrin and deltamethrin with wild-type and mutant *vgsc* residues across domains DIIS6, DIIIS6, and DIVS6

Insecticide	*vgsc* domain	Genotype (WT/Mutant)	Interaction/List of Interacting amino acid (Chain A)	Binding energy (kcal/mol)
Permethrin	DIIS6	A1022, E1041 (WT)	Hydrophobic ASN9A, PHE13A, LEU18A, PRO19A, ASN22A, PHE23A, THR24A, ILE32A, ASP47A, and VAL51A	− 7.9
		A1022S (Mutant A)	Hydrophobic PHE13A, PHE23A, and VAL51A Halogen Bonds ASN9A	− 6.9
		A1022P (Mutant B)	Hydrophobic PRO6A, PHE13A, PRO19A, ASN22A, PHE23A, THR24A, and VAL51A Hydrogen Bonds ASN9A	-7.6
		E1041K (Mutant C)	Hydrophobic PHE13A, PRO19A, ASN22A, PHE23A, and PHE59A Halogen Bonds SER55A	− 6.9
		A1022S + E1041K (Mutant A + C)	Hydrophobic PHE13A, PRO19A, PHE23A, ASP47A, and VAL51A Halogen Bonds CYS48A	− 6.5
		A1022P + E1041K (Mutant B + C)	Hydrophobic PRO6A, PHE13A, PRO19A, ASN22A, THR24A, and VAL51A Hydrogen Bonds ASN9A	− 7.5
	DIIIS6	F1534, P1585 (WT)	Hydrophobic Interactions MET9A, TYR12A, and PHE15A π-Stacking TYR12A	− 6.3
		F1534L (Mutant D)	Hydrophobic LEU11A, TYR12A, and PHE15A Hydrogen Bonds ASN6A	− 5.0
		P1585R (Mutant E)	Hydrophobic TYR12A, PHE15A, PHE19A, and PHE23A π-Stacking PHE19A	− 6.2
		F1534L + P1585R (Mutant D + E)	Hydrophobic LEU11A and PHE15A Hydrogen Bonds ASN6A π-Stacking TYR12A	− 5.1
	DIVS6	F1695 (WT)	Hydrophobic PHE60A, PHE68A, VAL71A, PHE83A, LEU92A, and LEU93A π-Stacking PHE83A	− 6.5
		F1695L (Mutant F)	Hydrophobic Interactions PHE60A, PHE63A, PHE67A, PHE68A, PHE83A, and LEU93A π-Stacking PHE67A	− 6.5
Deltamethrin	DIIS6	A1022, E1041 (WT)	Hydrophobic ASN9A, PHE13A, LEU18A, PRO19A, ASN22A, PHE23A, THR24A, and VAL51A	− 7.8
		A1022S (Mutant A)	Hydrophobic PHE23A, VAL51A, and PHE59A π-Stacking PHE59A	− 6.0
		A1022P (Mutant B)	Hydrophobic PHE13A, PRO19A, ASN22A, PHE23A, THR24A, and VAL51A	− 7.1
		E1041K (Mutant C)	Hydrophobic PHE13A, PRO19A, PHE23A, THR24A, and VAL51A Halogen Bonds SER29A	− 7.3
		A1022S + E1041K (Mutant A + C)	Hydrophobic PHE13A, LEU18A, PRO19A, PHE23A, THR24A, ASP47A, and VAL51A	− 7.0
		A1022P + E1041K (Mutant B + C)	Hydrophobic ASN9A, PHE13A, ASN22A, VAL51A, and PHE59A π-Stacking PHE59A	− 6.5
	DIIIS6	F1534, P1585 (WT)	Hydrophobic TYR8A, LEU11A, PHE15A, and PHE16A π-Stacking TYR12A	− 6.6
		F1534L (Mutant D)	Hydrophobic TYR8A, LEU11A, TYR12A, and PHE15A π-Stacking TYR12A	− 6.2
		P1585R (Mutant E)	Hydrophobic TYR8A, LEU11A, TYR12A, PHE15A, and PHE16A π-Stacking TYR12A	− 6.7
		F1534L + P1585R (Mutant D + E)	Hydrophobic TYR8A, LEU11A, TYR12A, and PHE15A π-Stacking TYR12A	− 6.2
	DIVS6	F1695 (WT)	Hydrophobic PHE60A, PHE63A, PHE67A, PHE68A, VAL71A, LEU77A, PHE83A, and LEU93A	− 6.1
		F1695L (Mutant F)	Hydrophobic Interactions VAL56A, PHE60A, PHE63A, PHE67A, and PHE68A Hydrogen Bonds GLY64A π-Stacking PHE60A	− 6.1

Binding affinities and interacting amino acids (Chain A) are presented for substitutions at positions A1022, E1041, F1534, P1585, and F1695, including combined mutations

WT: wild type. Binding energy (kcal/mol) represents the predicted ligand–protein binding affinity calculated using the AutoDock Vina scoring function, where the lower energy indicates stronger binding. Interactions include hydrophobic contacts, hydrogen bonds, halogen bonds, and π-stacking between insecticides and *vgsc* residues (Chain A) were identified using PLIP. Amino acid residues are presented with position and chain identifier (A)

## Discussion

Chemical insecticide strategies face increasing challenges due to widespread mosquito resistance [55], undermining vector control efficacy globally. In Malaysia, outdated and fragmented resistance data further amplify these concerns. This study provides an updated assessment of insecticide susceptibility and evaluates the contribution of *vgsc* target-site mutations to pyrethroid resistance in *Ae. albopictus* across Northern Peninsular Malaysia.

### Resistance in Northern Peninsular Malaysian *Ae. albopictus*

The results emphasize a major concern regarding the widespread resistance to permethrin, deltamethrin, pirimiphos-methyl, and propoxur among adults, as well as resistance to temephos in *Ae. albopictus* larvae. In Malaysia, temephos is the primary larvicide used to treat mosquito larvae in stagnant water. Abate, a potent temephos-derived larvicide, has been in use since 1973 [56], contributing to the emergence of resistant larvae following prolonged reliance on a single larvicide [57, 58]. The widespread use of temephos larvicides in agricultural settings may further reduce its effectiveness [57]. This is consistent with the observed resistance in KB and KTT larval populations, where abundant stagnant water and rich microbial environments may influence larvicide persistence and efficacy. Variability in susceptibility may reflect differences in exposure history [8], acetylcholinesterase insensitivity (*Ace-1*) [58, 59], and enhanced metabolic detoxification [60], all shaped by local selective pressures.

Adult *Ae. albopictus* populations exhibited variable susceptibility patterns across regions, irrespective of dengue classification. The low mortality observed across all populations exposed to pyrethroids, organophosphates, and carbamates indicates resistance, consistent with recent Malaysian findings [6, 19, 61]. These results indicate reduced susceptibility that may compromise control efficacy and warrant further evaluation using resistance intensity assays. The observed carbamate resistance aligns with reports from multiple regions, including Malaysia, Thailand, India, and China [16, 43, 62–67], likely reflecting long-term use in agriculture and public health [14, 68]. Similarly, resistance to pirimiphos-methyl is consistent with continued organophosphate application in vector control programmes, including thermal fogging and indoor residual spraying [17, 18, 20].

Despite confirmed resistance to pyrethroids based on WHO mortality criteria, clear differences were observed between permethrin and deltamethrin responses across populations. Permethrin produced measurable knockdown in all populations, with moderately increased KdT_50_ values in Penang (SA: 42.73 min; BP: 35.91 min; RR_50_ = 1.14–1.35) and substantially prolonged knockdown times in Perlis (KB: 95.19 min; KTT: 73.02 min; RR_50_ = 2.32–3.02), indicating reduced susceptibility and elevated resistance intensity. In contrast, deltamethrin remained highly effective in Penang populations, with rapid knockdown (KdT_50_ < 17 min; RR_50_ < 1), consistent with previous Malaysian reports [69, 70], but was markedly ineffective in Perlis. In KB, knockdown was < 5%, precluding KdT estimation, while KTT exhibited extremely prolonged KdT_50_ (768.86 min) and KdT_95_ (> 7000 min), indicating pronounced resistance. These findings demonstrate substantial heterogeneity in pyrethroid response, with markedly higher resistance intensity in Perlis populations, particularly against deltamethrin. This pattern is likely influenced by prolonged use of Type II pyrethroids in local vector control programmes, as reported by the Perlis Vector and Pest Unit, where sustained application of insecticides such as cypermethrin has been practised over several years. Given the shared mode of action and structural similarity among Type II pyrethroids [71], such exposure may promote cross-resistance to deltamethrin. In contrast, the comparatively lower resistance intensity to permethrin is consistent with reports of limited or variable resistance in Malaysian *Ae. albopictus* populations [7, 16, 61, 72].

These regional differences in resistance profiles likely reflect underlying ecological and operational heterogeneity between Penang and Perlis. The Penang sites represent urban and peri-urban settings with mixed residential and industrial activities, whereas Perlis sites are predominantly agricultural and located near the Thailand border. Such environmental contrasts may shape mosquito breeding ecology, insecticide exposure patterns, and the intensity of selection pressures [73]. In agricultural landscapes, repeated exposure to agrochemicals, particularly the widespread use of pyrethroids such as cypermethrin in Perlis may promote cross-resistance and could contribute to the elevated resistance intensity and prolonged knockdown times observed in these populations. In contrast, the absence of documented insecticide usage histories for Penang limits direct comparison of selection pressures between regions. Similarly, the lack of baseline susceptibility data for Perlis constrains interpretation of whether the observed resistance reflects recent emergence or long-term establishment. Differences in local vector control practices, including insecticide type, application frequency, and rotation strategies, may further influence resistance trajectories [74]. Seasonal variation in mosquito abundance and environmental conditions such as rainfall, temperature, and water availability may also modulate population turnover and insecticide exposure [75], thereby affecting allele frequency dynamics and the selection of *vgsc* target-site mutations.

### Genotype–phenotype associations and their relationship to pyrethroid insecticides affinity

Insecticide resistance in mosquitoes is often mediated by the interaction of multiple mechanisms. In *Ae. albopictus*, pyrethroid resistance is frequently associated with both metabolic resistance and target-site resistance [76]. Local evidence of target-site resistance emerged following the identification of the F1534L variant in permethrin-resistant *Ae. albopictus* from Kuala Lumpur [19], indicating an increasing role of sodium channel mutations in Malaysia. In the present study, F1534L was detected at frequencies of 5% in Penang and 32.5% in Perlis, representing its second report in the country and suggesting ongoing spatial spread, particularly in Northern populations. The origin of this mutation in Malaysia remains unclear and warrants broader geographic surveillance. Globally, F1534L (TTC to TTG or CTC) has been reported across diverse regions, including Florida, USA (2011) [77], Italy (2011) [23], China (2014–2020) [24, 31, 34, 68, 78, 79], and Indonesia (2024) [29], supporting its recurrent emergence or dissemination under insecticide pressure. At the mutation level, F1534L showed only a weak association (*P* < 0.05) between heterozygous genotypes (TTC/TTG) and permethrin or deltamethrin resistance in Penang and Perlis populations (Additional file 6: Table S10(D)), consistent with reports of no significant association for this mutation [31, 79, 80], although protective effects against deltamethrin have been described elsewhere [63, 81]. This limited association may reflect allele dosage effects, biochemical susceptibility thresholds, context-dependent fitness trade-offs, and the small sample sizes for each phenotype [82, 83]. This reflects the stratified mutation-level design, which results in small subgroup sizes and may limit statistical power; therefore, association estimates should be interpreted with caution.

Multiple mutations (A1022S/P, E1041K, P1585R, and F1695L) co-occurred with F1534L in *Ae. albopictus* populations from Penang and Perlis (*P* < 0.05) were strongly associated with resistance to permethrin and deltamethrin, especially in dengue outbreak areas. This pattern suggests that increasing mutation burden is associated with higher resistance. However, no regression model was applied to formally test additive or interaction (epistatic) effects among loci. Therefore, these observations should be interpreted as descriptive cumulative associations, and the specific statistical nature of locus-locus interaction cannot be determined within the present analytical framework. Similar multi-mutation patterns have been reported in *Ae. albopictus* and other mosquito species [29, 84, 85]. Additionally, compensatory mutations (A1022S/P, E1041K, P1585R, and F1695L) may offset fitness costs associated with *kdr* substitutions [86], which could contribute to the persistence and co-occurrence of multiple mutations observed in this study.

To provide structural context, docking analyses of the DIIS6, DIIIS6, and DIVS6 segments revealed consistent reductions in permethrin and deltamethrin binding affinity associated with *vgsc* mutations, despite most substitutions being distal from the predicted binding pocket. These findings suggest that mutations may alter local structural properties or interaction geometry within S6 helices rather than directly disrupting ligand contact residues. Such localized effects are biologically plausible given the functional role of S6 segments in pyrethroid binding and channel gating [87–89].

Although the docking analyses were restricted to individual S6 domains rather than the full-length *vgsc* protein, this hotspot-focused approach targets regions that harbour the majority of functionally validated *kdr* mutations [89–91]. Accordingly, the docking results are interpreted as supportive regional structural inference rather than full mechanistic modelling. The observed concordance between reduced predicted binding affinity and increased resistance frequency provides complementary context for the genotype–phenotype associations.

Single substitutions were generally associated with modest reductions in predicted binding affinity, consistent with their weak phenotypic associations. In contrast, combined mutations predicted to show greater reductions in binding affinity within DIIS6 (A1022S + E1041K) and DIIIS6 (F1534L + P1585R), aligning descriptively with the higher resistance odds observed for multi-mutation genotypes. Mutations such as F1695L (DIVS6) exhibited minimal predicted effects when present alone but were more frequently associated with multi-locus backgrounds, suggesting a potential modifier role for secondary substitutions [90, 91]. Nevertheless, taking into account that *vgsc* is a transmembrane protein, it is important to note that simulating the natural environment by applying molecular dynamic simulation (MDS) provides better accuracy. MDS may consider the fluidity and environment of the membrane, the time factor, and the flexibility of the protein, resulting in higher accuracy based on trajectory observation rather than assumption, as in the docking approach. Docking analysis nevertheless provides useful preliminary structural insight into how mutations may influence ligand interactions.

Although deltamethrin generally exhibited stronger absolute *vgsc* binding affinities than permethrin, mosquitoes exposed to deltamethrin carried higher *vgsc* mutation burdens. As a Type II pyrethroid, deltamethrin induces prolonged sodium channel opening and stronger neurotoxic effects, potentially requiring multiple structural alterations to reduce sensitivity [88]. This interpretation is consistent with both the docking results and population-level observations, which indicate that resistance to deltamethrin is more frequently associated with multi-mutation genotypes [92, 93].

Detailed interaction analysis further supports these predictive findings (Table 4). Across domains, wild-type sequences consistently exhibited lower binding energies and a greater number of interacting residues, indicating stronger ligand affinity. In contrast, mutant and double-mutant forms showed reduced interaction profiles and higher binding energies. For example, in DIIS6, the wild-type A1022 residue displayed strong interaction with permethrin (− 7.9 kcal/mol; 10 hydrophobic interactions), whereas the A1022S substitution was associated with reduced predicted binding affinity (− 6.9 kcal/mol; three hydrophobic interactions). The A1022P substitution was predicted to have a comparatively moderate effect (− 7.6 kcal/mol; seven hydrophobic and one halogen interaction), suggesting that amino acid properties may influence ligand binding. In some cases, predicted ligand repositioning was associated with altered interaction types from stronger hydrophobic interactions to weaker hydrogen bonds, π–π stacking, or halogen interactions, further contributing to reduced predicted binding stability. Notably, both permethrin and deltamethrin were predicted to show stronger affinity for DIIS6 relative to other domains.

In summary, integrating genotype–phenotype associations with hotspot-focused docking is consistent with a cumulative multi-locus pattern of target-site resistance in *Ae. albopictus*. Although interaction effects were not formally modelled, the co-occurrence and accumulation of *vgsc* mutations are consistent with insecticide-driven selection. These findings highlight the importance of considering multi-mutation genotypes in resistance monitoring and underscore the need for further investigation into their effects on haplotype structure and population-level genetic variation.

### Impacts of insecticide-driven selection on the emergence of haplotypes and genetic variation

Prolonged pyrethroid use in agriculture and vector control imposes strong selective pressure on *Ae. albopictus*, accelerating the emergence and persistence of resistance-associated alleles [94]. In this study, high haplotype diversity (Hd = 0.872–0.961) coupled with low to moderate nucleotide diversity (π = 0.008–0.012) across partial *vgsc* domains indicates numerous closely related haplotypes, consistent with recent population expansion or rapid mutation accumulation under selective pressure rather than long-term neutral divergence [95].

DIIS6 exhibited the strongest signal of adaptive evolution, supported by a significantly negative Tajima’s D (− 1.921, *P* < 0.01), highly negative Fu’s *Fs* (− 29.893), and a higher proportion of non-synonymous mutations, suggesting directional selection driven by pyrethroid exposure [90]. In contrast, DIIIS6 and DIVS6 showed weaker or mixed evolutionary signals, with non-significant neutrality tests despite negative Fu’s *Fs* values, consistent with purifying selection and/or demographic processes [53, 54]. These findings highlight domain-specific selective pressures acting on functionally important regions of the *vgsc* gene.

Haplotype network analyses further provide insights into resistance evolution. The predominance of the ancestral wild-type haplotype (H3; V1016/A1022/E1041) supports a pre-insecticide genetic background, from which derived haplotypes carrying A1022S/P and A1022S/P + E1041K likely arose through stepwise mutational events. In DIIIS6, *kdr* mutations (F1534L, P1585R) occurring on distinct haplotype backgrounds suggest patterns consistent with multiple independent origins rather than a single selective sweep [23, 96, 97]. The restriction of specific haplotypes (H17, H19) to Perlis suggests localized selection pressures, whereas shared haplotypes between Penang and Perlis are consistent with ongoing gene flow. The reticulated network structure supports stepwise mutation and complex mutational processes generating diverse resistance-associated genotypes [98]. The absence of D1763 *kdr* mutations in DIVS6, despite high haplotype diversity, suggests that this domain may be under different selective constraints.

At the population level, heterogeneous genetic diversity among resistant groups may reflect patterns of multiple independent mutational events rather than the expansion of a single dominant lineage [23, 96]. Geographic differences between Penang and Perlis, characterized by both population-specific and shared haplotypes, further support a balance between localized selection pressures and population connectivity, likely influenced by ecological heterogeneity, land use, and insecticide exposure intensity [99, 100]. Notably, the proximity of Perlis to the Thailand border suggests potential cross-border gene flow, with mosquito dispersal and human-mediated movement facilitating the regional spread of resistance-associated alleles, as reported in neighbouring Southeast Asia [93, 101].

Nonetheless, this study has limitations in providing quantitative estimates of genetic structuring between Penang and Perlis. Although metrics such as F < sub > ST < /sub > and AMOVA could be applied, their interpretation would be constrained by reliance on a single candidate gene under strong selection. As the *vgsc* locus is non-neutral, inferred differentiation would reflect both selective and demographic processes, limiting the ability to disentangle gene flow from insecticide-driven selection [102]. Thus, the observed patterns should be interpreted as locus-specific signals of adaptive differentiation rather than indicators of genome-wide population structure. Future studies incorporating neutral, multi-locus markers, and broader sampling are needed for robust population genetic inference.

Overall, these findings are suggestive of a model of resistance evolution shaped by directional selection, demographic processes, and gene flow. Integrating genetic, phenotypic, and ecological data is essential to understand resistance emergence and spread. Within this framework, the contribution of metabolic resistance alongside target-site mutations is essential to fully explain the observed resistance patterns.

### Role of metabolic resistance in Northern Peninsular Malaysian *Ae. albopictus*

PBO synergism assays highlighted the contribution of cytochrome P450-mediated metabolic resistance alongside *vgsc* mutations. Some susceptible individuals carried *vgsc* mutations, suggesting target-site resistance alone is insufficient and supporting the contribution of additional mechanisms, particularly metabolic detoxification pathways [12, 13]. In Penang, substantial restoration of susceptibility after PBO exposure indicates strong cytochrome P450 involvement. Perlis populations showed strong synergism for permethrin but limited effect for deltamethrin, reflecting possible differences in metabolic pathways or enzyme specificity. These findings are consistent with regional studies where PBO did not fully restore pyrethroid susceptibility [103]. Overall, PBO-based interventions may help manage resistance, but effectiveness varies by insecticide and population.

## Conclusions

This study provides a comprehensive update on insecticide resistance in *Ae. albopictus* across Northern Peninsular Malaysia, revealing widespread resistance to pyrethroids, organophosphates, and carbamates. Notably, we report the discovery of novel multi-mutation *vgsc* genotypes (A1022S/P, E1041K, P1585R, and F1695L) that co-occur with the F1534L mutation, which is spreading in Penang and Perlis populations. These multi-locus genotypes, alongside elevated cytochrome P450-mediated metabolic detoxification, underlie high resistance intensity, particularly in agricultural and border-adjacent Perlis populations. Haplotype analyses suggest patterns consistent with multiple independent mutational events, with localized selection pressures and gene flow potentially contributing to complex population structures. Structural modelling of *vgsc* domains provides supportive evidence for the potential cumulative effects of mutations on predicted pyrethroid binding, complementing genotype–phenotype observations. Collectively, these findings underscore the urgent need for integrated, evidence-driven vector control strategies, including insecticide rotation, synergist-based interventions, and continuous resistance surveillance, to mitigate the emergence and spread of complex resistance profiles in Malaysian *Ae. albopictus* populations.

## Supplementary Information

**Additional file 1: Table S1.** Lists of primers used in this study.

**Additional file 2: Figure S1.** Ramachandran plot. Structural validation based on Ramachandran plot analysis.

**Additional file 3: Table S2.** Percentage mortality of females *Ae. albopictus* at 24 h post-treatment against diagnostic dose of 0.012 ppm and 0.034 ppm temephos larvicides, respectively. Figure S2. Percentage mortality of *Ae. albopictus* larvae from the different localities in Penang and Perlis, Malaysia towards 0.012 ppm and 0.034 ppm of temephos. Error bars represent 95% confidence interval (CI). Black horizontal line indicates resistant threshold level (mortality < 90% is considered phenotypically resistant; WHO, 2016). Table S3. Summary of LC_50_, LC_95_, diagnostic dose (DD), and resistance ratios (RR) from larval bioassays of temephos in field strains of *Ae. albopictus* compared with the USM_VCRU laboratory strain.

**Additional file 4: Table S4.** Percentage mortality of females *Ae. albopictus* at 24 h post-treatment against several insecticides from carbamate, organophosphate and pyrethroid classes, together with 4% PBO as a synergist for pyrethroid insecticides. Table S5. Knockdown rates of *Ae. albopictus* in Penang and Perlis, Malaysia against pyrethroid adulticides.

**Additional file 5: Table S6.** Distributions of genotypes in *vgsc* gene domains of pyrethroids susceptible and resistant *Ae. albopictus* across Northern Peninsular Malaysia (NPM) populations. Table S7. Distributions of prevalent mutations in *vgsc* gene domains of pyrethroids susceptible and resistant *Ae. albopictus* across Northern Peninsular Malaysia (NPM) populations. Table S8. Distribution of single-, duo-, triple-, and quadruple-loci of the genotypic combination in DIIS6, DIIIS6, and DIVS6 of *vgsc* gene in field-collected *Ae. albopictus*.

**Additional file 6: Table S9.** Individual-level genotype–phenotype associations between *vgsc* mutation burden and pyrethroid resistance in *Ae. albopictus*. Table S10. Genotype–phenotype association based on a single mutation-specific. (A) Association of genotype F1534L with the resistance phenotype towards pyrethroid insecticides for the Penang and Perlis strains of *Ae. albopictus*. (B) Association of genotype A1022S/P with the resistance phenotype towards pyrethroid insecticides for the Penang and Perlis strains of *Ae. albopictus*. (C) Association of genotype E1041K with the resistance phenotype towards pyrethroid insecticides for the Penang and Perlis strains of *Ae. albopictus*. (D) Association of genotype P1585R with the resistance phenotype towards pyrethroid insecticides for the Penang and Perlis strains of *Ae. albopictus*. (E) Association of genotype F1695L with the resistance phenotype towards pyrethroid insecticides for the Penang and Perlis strains of *Ae. albopictus*.

**Additional file 7: Table S11.** Zygosity distribution of single-locus mutations by strain and phenotype of the permethrin and deltamethrin exposed *Ae. albopictus*. Table S12. Zygosity distribution of multi-locus mutations by strain and phenotype of the permethrin and deltamethrin exposed *Ae. albopictus*.

**Additional file 8: Table S13.** Haplotypes frequency. (A) Forty-one haplotypes were identified from 90 nucleotide sequences of DIIS6 in *vgsc* gene fragment of local *Ae. albopictus* in Penang and Perlis, and deposited in the NCBI GenBank. (B) Twenty-five haplotypes were identified from 90 nucleotide sequences of DIIIS6 in *vgsc* gene fragment of local *Ae. albopictus* in Penang and Perlis, and deposited in the NCBI GenBank. (C) Fifty haplotypes were identified from 90 nucleotide sequences of DIVS6 in *vgsc* gene fragment of local *Ae. albopictus* in Penang and Perlis, and deposited in the NCBI GenBank. Figure S3. Haplotype network showing the genealogical relationships of 41 haplotypes in DIIS6 based on four strains of Northern Peninsular Malaysia populations of *Ae. albopictus*: wild-type haplotypes are depicted in black, haplotypes with *kdr* mutations associated with resistance phenotypes are marked in red, and those with new non-synonymous mutations are highlighted in blue. Figure S4. Haplotype network showing the genealogical relationships of 50 haplotypes in DIVS6 based on four strains of Northern Peninsular Malaysia populations of *Ae. albopictus*: wild-type haplotypes are depicted in black, haplotypes with *kdr* mutations associated with resistance phenotypes are marked in red, and those with new non-synonymous mutations are highlighted in blue.

**Additional file 9: Figure S5.** Structural alignment of partial *vgsc* protein domains. (A) Structural alignment of partial *vgsc* gene domains DIIS6 between wild-type and mutant variants. (B) Structural alignment of partial *vgsc* gene domains DIIIS6 between wild-type and mutant variants. (C) Structural alignment of partial *vgsc* gene domains DIVS6 between wild-type and mutant variants. Figure S6. Protein–ligand interactions. (A) Positional difference binding of permethrin against the wild-type and mutant variants in DIIS6. (B) Positional difference binding of permethrin against the wild-type and mutant variants in DIIIS6. (C) Positional difference binding of permethrin against the wild-type and mutant variants in DIVS6. (D) Positional difference binding of deltamethrin against the wild-type and mutant variants in DIIS6. (E) Positional difference binding of deltamethrin against the wild-type and mutant variants in DIIIS6. (F) Positional difference binding of deltamethrin against the wild-type and mutant variants in DIVS6.

## Data Availability

All data generated or analysed during this study are included in this published article and its supplementary information files. The sequences generated in this study have been deposited in GenBank under accession numbers PQ660263-PQ660378.

## Abbreviations

WHO: World Health Organization
vgsc: Voltage-gated sodium channel
PCR: Polymerase chain reaction
SNP: Single-nucleotide polymorphism
Ace-1: Acetylcholinesterase
MDS: Molecular Dynamic Simulation
kdr: Knockdown resistance
VCRU: Vector Control Research Unit
PBO: Piperonyl butoxide
PLIP: Protein–Ligand Interaction Profiler
BLAST: Basic Local Alignment Search Tool
DDT: Dichlorodiphenyltrichloroethane
RR: Resistance ratio
OR: Odds ratio
vs: Versus
