# Supplementary material for: Emerging and regionally novel voltage-gated sodium channel mutations associated with pyrethroid resistance and altered binding affinity in Aedes albopictus (Skuse) from Northern Peninsular Malaysia
Source: Parasit Vectors. 2026 Jun 21;19:321. doi: 10.1186/s13071-026-07496-w (PMC13449526; doi:10.1186/s13071-026-07496-w)
Supplement: Supplementary file 1 [file 13071_2026_7496_MOESM1_ESM.pdf]

Additional file 1: Table S1: Lists of primers used in this study.

Domain	Primer name and sequences (5'-3')	Size (bp)	References
**DII**	AlbSCF22: TTA TCC CTG CTC CGC CAT CAG<br>AlbSCR23: GAC AGC GAC GAT GAA CCG AA	~ 414	Primer designed
**DIII**	Alb171F: CCG ATT CGC GAG ACC AAC AT<br>AegSCR8: TAG CTT TCA GCG GCT TCT TC	~ 220	Ahmad et al. (2022)
**DIV**	AlbSCF6: TCG AGA AGT ACT TCG TGT CG<br>AlbSCF8: AAC AGC AGG ATC ATG CTC TG	~ 280	Kasai et al. (2011)
