# Supplementary material for: Emerging and regionally novel voltage-gated sodium channel mutations associated with pyrethroid resistance and altered binding affinity in Aedes albopictus (Skuse) from Northern Peninsular Malaysia
Source: Parasit Vectors. 2026 Jun 21;19:321. doi: 10.1186/s13071-026-07496-w (PMC13449526; doi:10.1186/s13071-026-07496-w)
Supplement: Supplementary file 2 [file 13071_2026_7496_MOESM2_ESM.pdf]

Additional file 2: Fig. S1. Ramachandran plot. Structural validation based on Ramachandran plot analysis.

DII_WildType_A1022

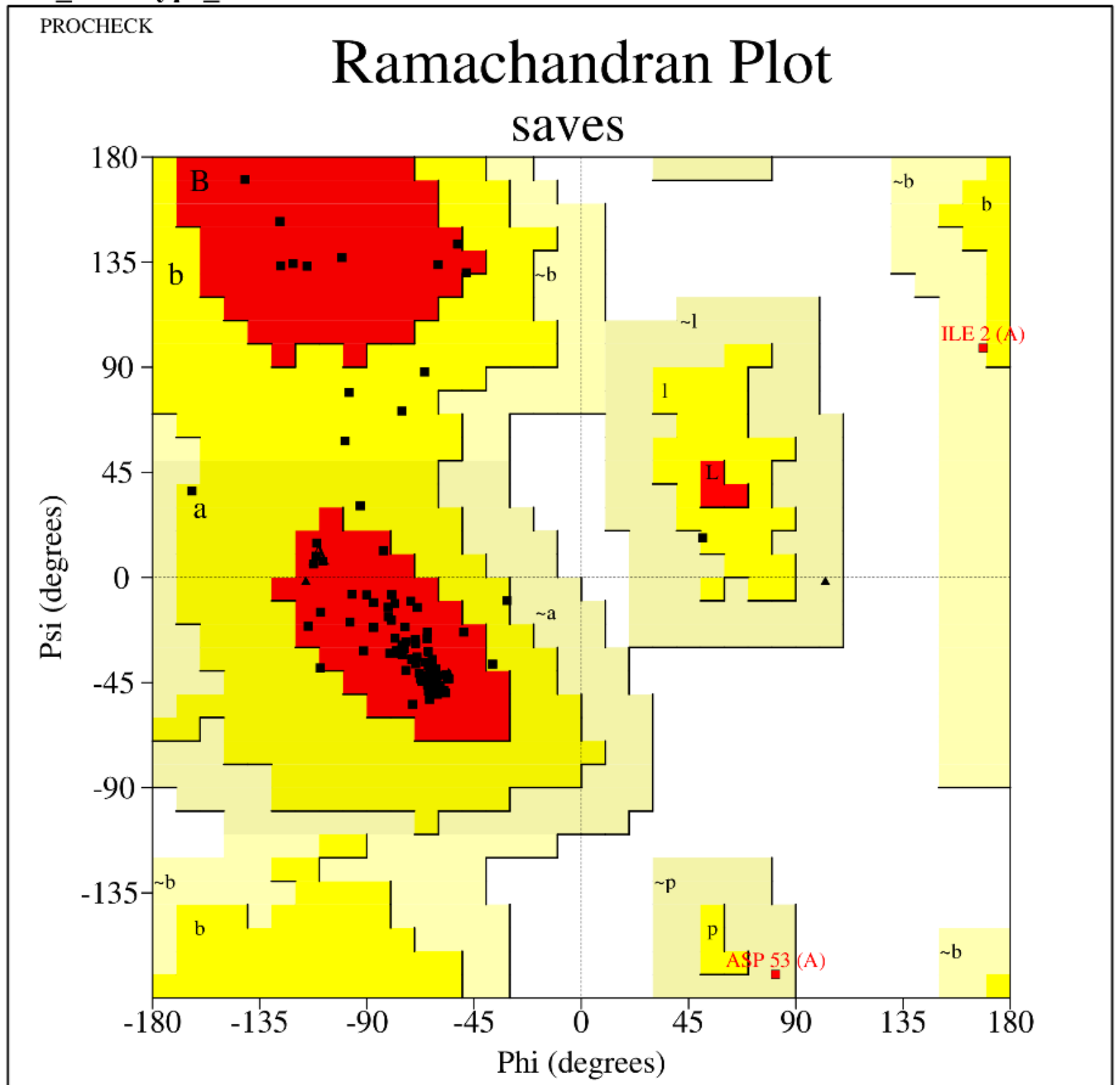

Plot statistics

Residues in most favoured regions [A,B,L]	87	91.6%
Residues in additional allowed regions [a,b,l,p]	6	6.3%
Residues in generously allowed regions [~a,~b,~l,~p]	2	2.1%
Residues in disallowed regions	0	0.0%
	----	------
Number of non-glycine and non-proline residues	95	100.0%
Number of end-residues (excl. Gly and Pro)	2	
Number of glycine residues (shown as triangles)	4	
Number of proline residues	7	

Total number of residues	108	

Based on an analysis of 118 structures of resolution of at least 2.0 Angstroms and R-factor no greater than 20%, a good quality model would be expected to have over 90% in the most favoured regions.

saves_01.ps

DII_Mutant_A_A1022S

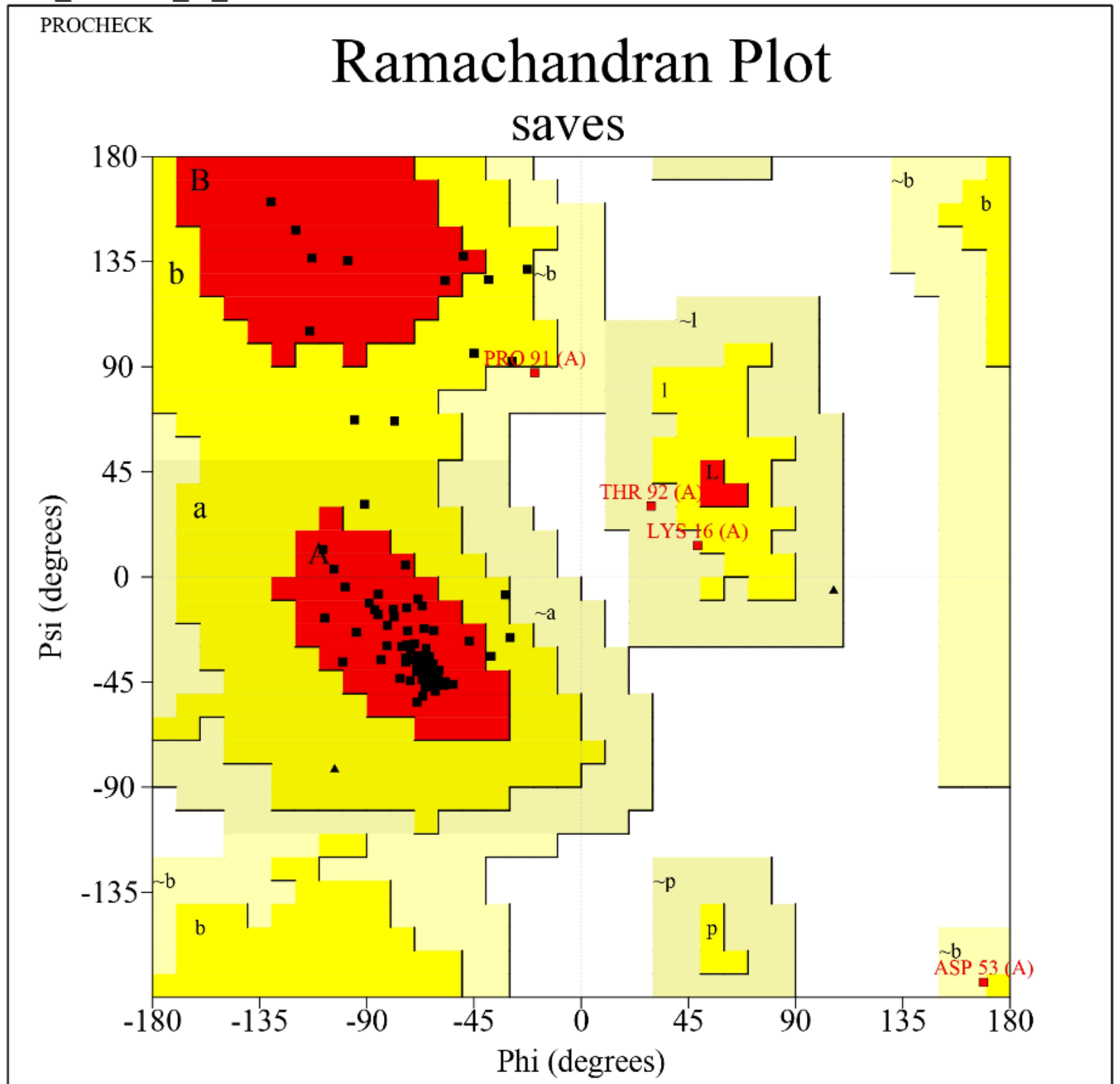

Plot statistics

Residues in most favoured regions [A,B,L]	85	89.5%
Residues in additional allowed regions [a,b,l,p]	7	7.4%
Residues in generously allowed regions [~a,~b,~l,~p]	3	3.2%
Residues in disallowed regions	0	0.0%
	----	------
Number of non-glycine and non-proline residues	95	100.0%
Number of end-residues (excl. Gly and Pro)	2	
Number of glycine residues (shown as triangles)	4	
Number of proline residues	7	

Total number of residues	108	

Based on an analysis of 118 structures of resolution of at least 2.0 Angstroms and R-factor no greater than 20%, a good quality model would be expected to have over 90% in the most favoured regions.

saves_01.ps

DII_Mutant_B_A1022P

PROCHECK

Ramachandran Plot

saves

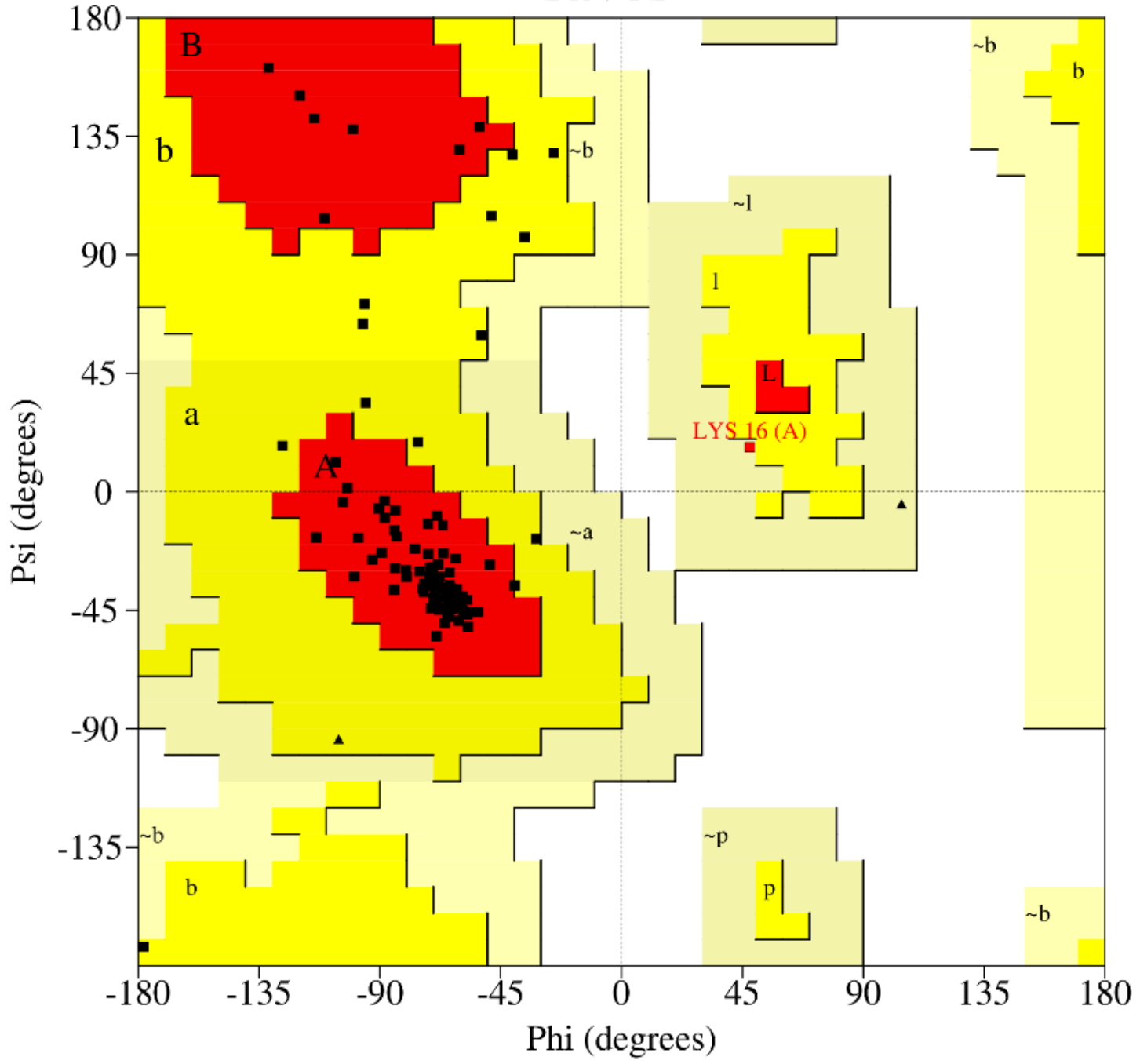

Plot statistics

Residues in most favoured regions [A,B,L]	84	89.4%
Residues in additional allowed regions [a,b,l,p]	9	9.6%
Residues in generously allowed regions [~a,~b,~l,~p]	1	1.1%
Residues in disallowed regions	0	0.0%
	----	------
Number of non-glycine and non-proline residues	94	100.0%
Number of end-residues (excl. Gly and Pro)	2	
Number of glycine residues (shown as triangles)	4	
Number of proline residues	8	

Total number of residues	108	

Based on an analysis of 118 structures of resolution of at least 2.0 Angstroms and R-factor no greater than 20%, a good quality model would be expected to have over 90% in the most favoured regions.

saves_01.ps

DII_Mutant_C_E1041K

PROCHECK

Ramachandran Plot

saves

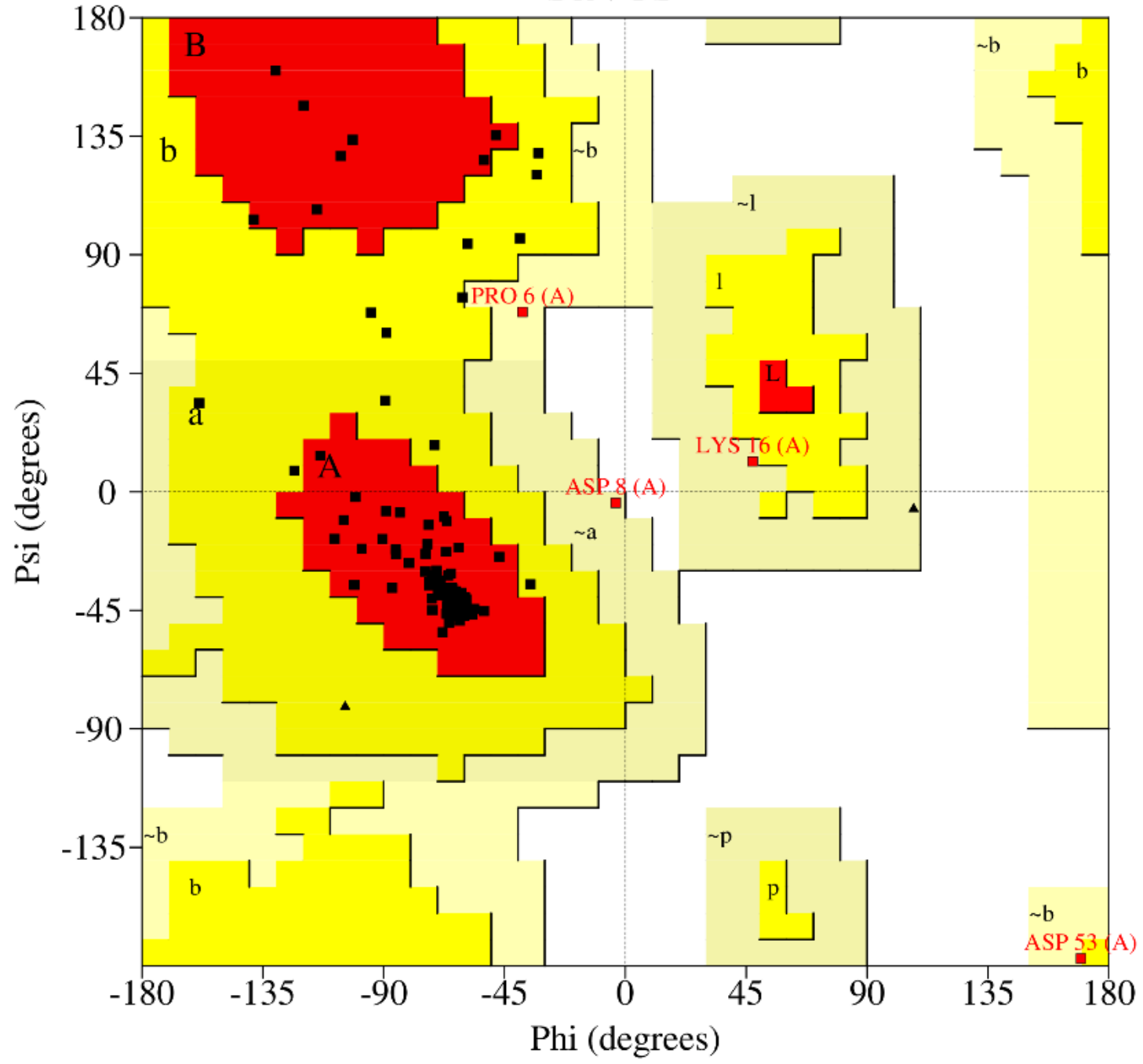

Plot statistics

Residues in most favoured regions [A,B,L]	83	87.4%
Residues in additional allowed regions [a,b,l,p]	9	9.5%
Residues in generously allowed regions [~a,~b,~l,~p]	3	3.2%
Residues in disallowed regions	0	0.0%
	----	------
Number of non-glycine and non-proline residues	95	100.0%
Number of end-residues (excl. Gly and Pro)	2	
Number of glycine residues (shown as triangles)	4	
Number of proline residues	7	

Total number of residues	108	

Based on an analysis of 118 structures of resolution of at least 2.0 Angstroms and R-factor no greater than 20%, a good quality model would be expected to have over 90% in the most favoured regions.

saves_01.ps

DII_Mutant_A+C_A1022S_E1041K

PROCHECK

Ramachandran Plot

saves

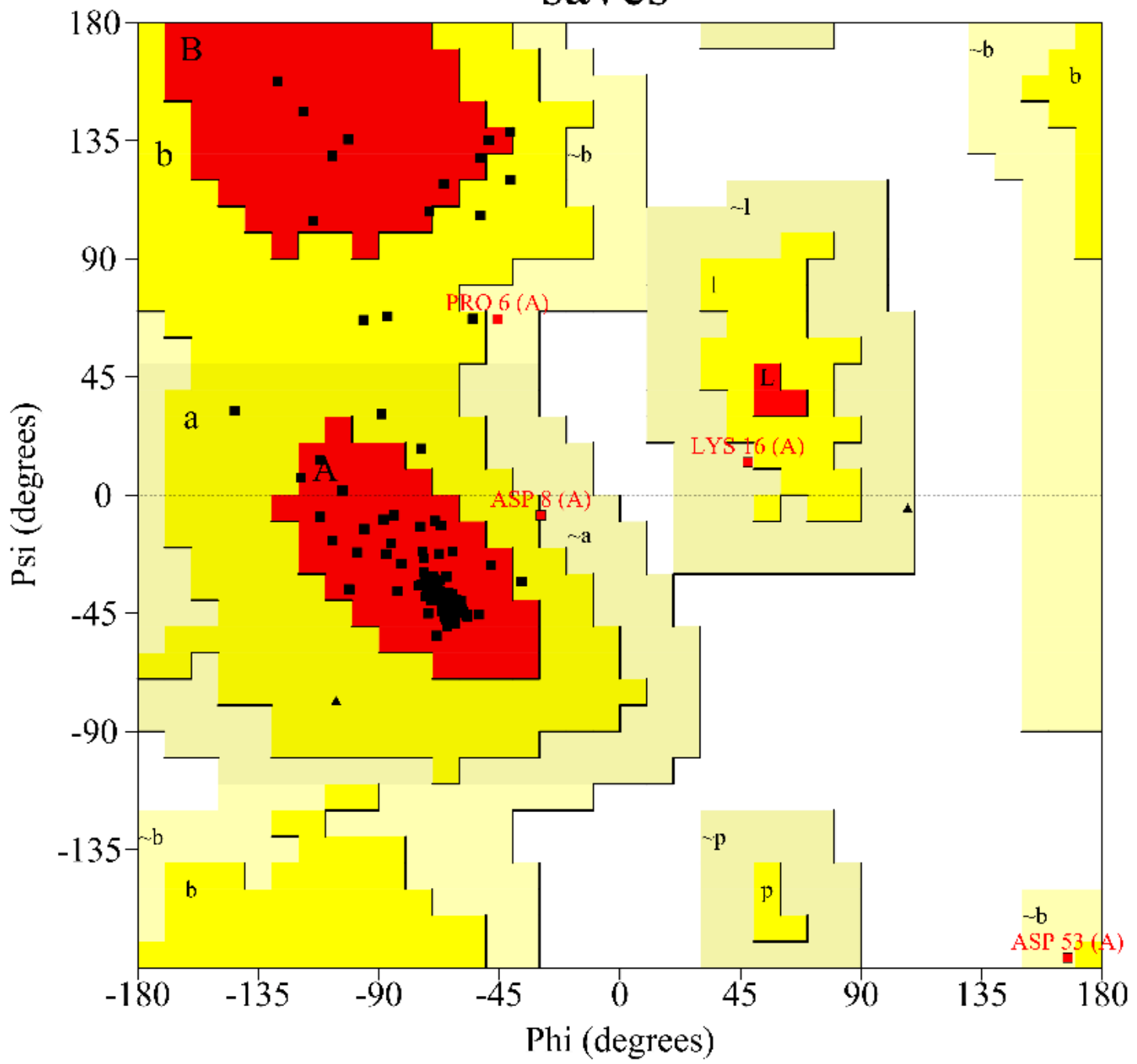

Plot statistics

Residues in most favoured regions [A,B,L]	85	89.5%
Residues in additional allowed regions [a,b,l,p]	7	7.4%
Residues in generously allowed regions [~a,~b,~l,~p]	3	3.2%
Residues in disallowed regions	0	0.0%
	----	------
Number of non-glycine and non-proline residues	95	100.0%
Number of end-residues (excl. Gly and Pro)	2	
Number of glycine residues (shown as triangles)	4	
Number of proline residues	7	

Total number of residues	108	

Based on an analysis of 118 structures of resolution of at least 2.0 Angstroms and R-factor no greater than 20%, a good quality model would be expected to have over 90% in the most favoured regions.

saves_01.ps

DII_Mutant_B+C_A1022P_E1041K

PROCHECK

Ramachandran Plot

saves

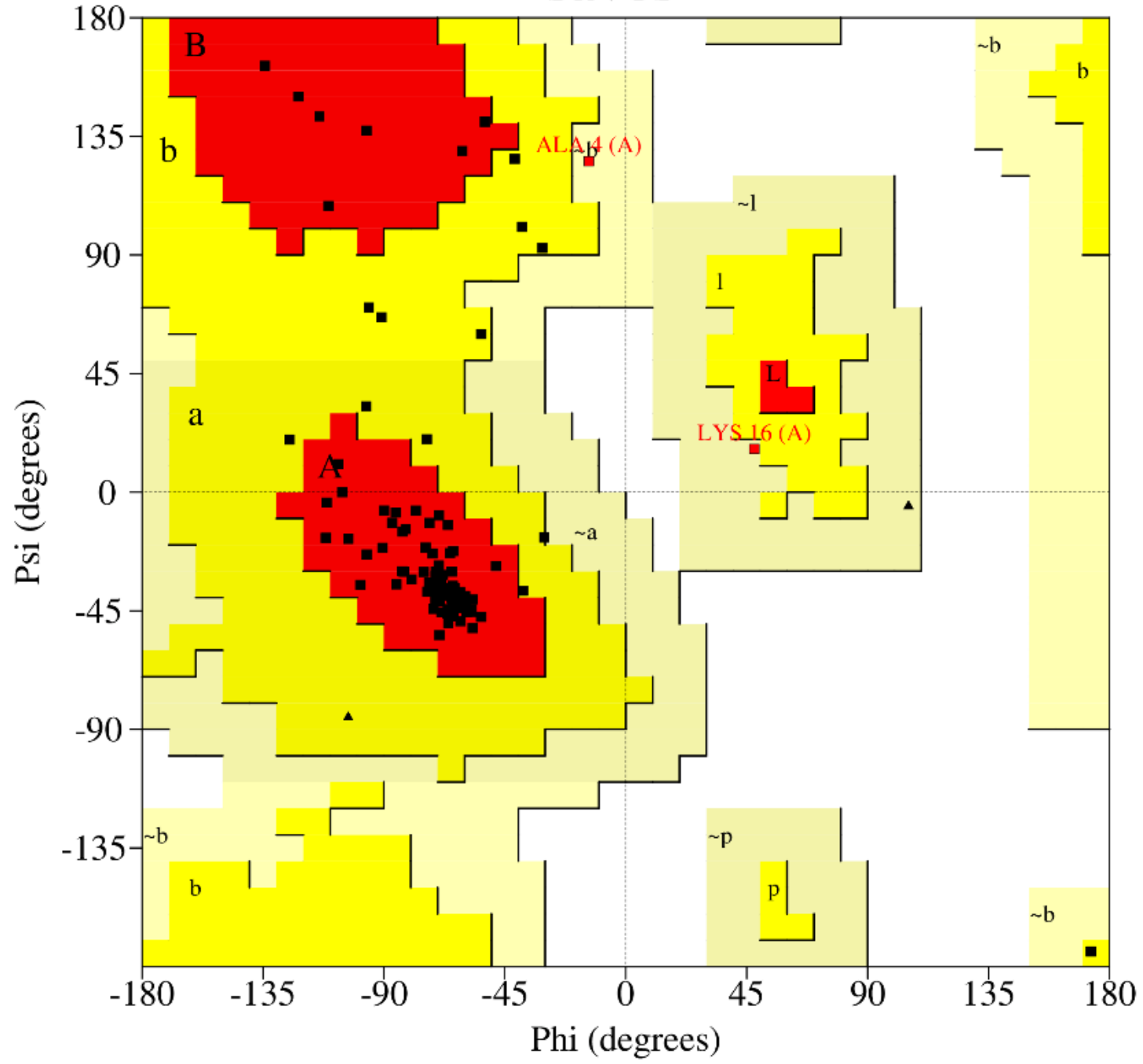

Plot statistics

Residues in most favoured regions [A,B,L]	84	89.4%
Residues in additional allowed regions [a,b,l,p]	8	8.5%
Residues in generously allowed regions [~a,~b,~l,~p]	2	2.1%
Residues in disallowed regions	0	0.0%
	----	------
Number of non-glycine and non-proline residues	94	100.0%
Number of end-residues (excl. Gly and Pro)	2	
Number of glycine residues (shown as triangles)	4	
Number of proline residues	8	

Total number of residues	108	

Based on an analysis of 118 structures of resolution of at least 2.0 Angstroms and R-factor no greater than 20%, a good quality model would be expected to have over 90% in the most favoured regions.

saves_01.ps

DIII_WildType_F1534_P1585

PROCHECK

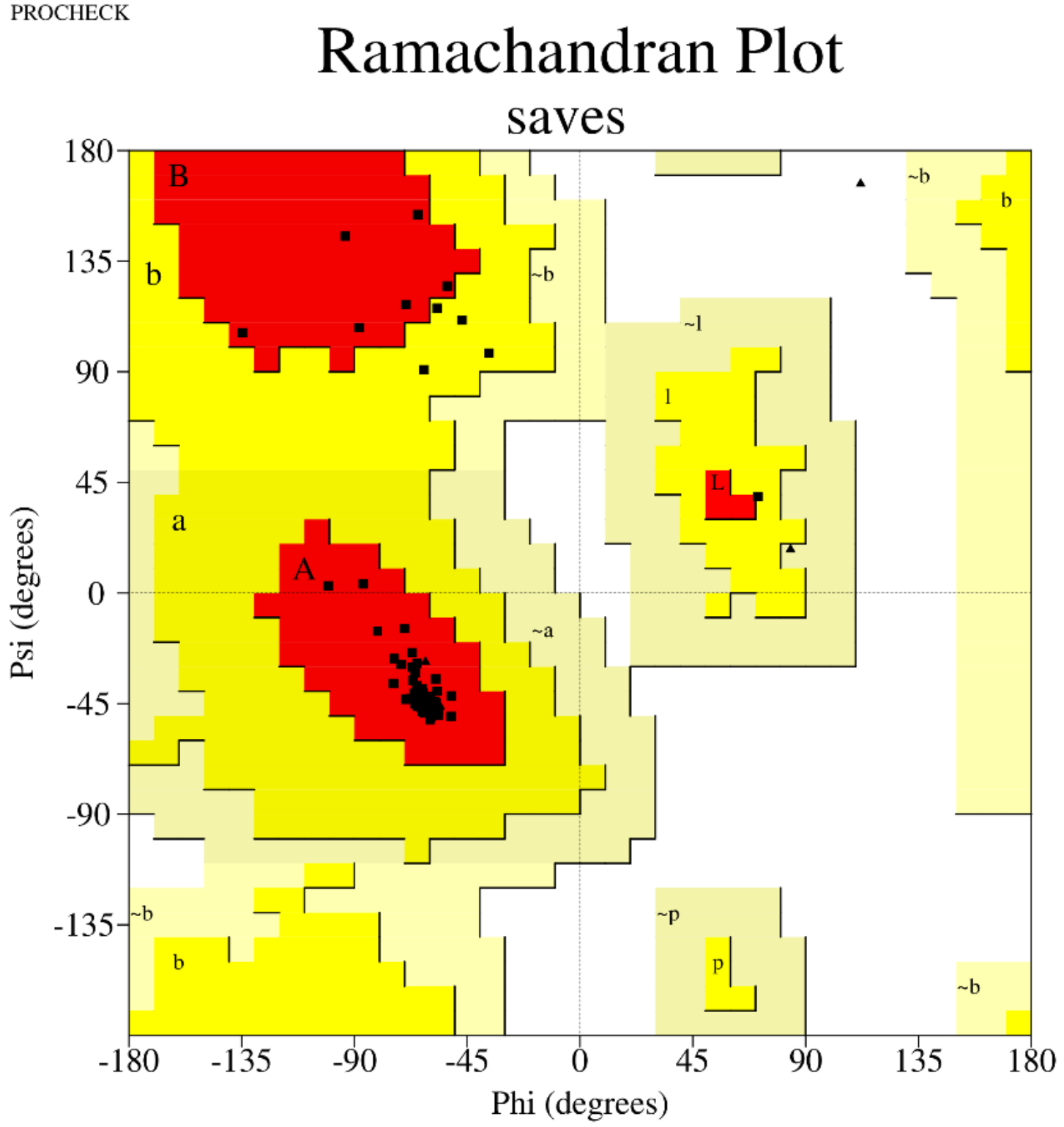

Plot statistics

Residues in most favoured regions [A,B,L]	61	93.8%
Residues in additional allowed regions [a,b,l,p]	4	6.2%
Residues in generously allowed regions [~a,~b,~l,~p]	0	0.0%
Residues in disallowed regions	0	0.0%
	----	------
Number of non-glycine and non-proline residues	65	100.0%
Number of end-residues (excl. Gly and Pro)	1	
Number of glycine residues (shown as triangles)	5	
Number of proline residues	2	

Total number of residues	73	

Based on an analysis of 118 structures of resolution of at least 2.0 Angstroms and R-factor no greater than 20%, a good quality model would be expected to have over 90% in the most favoured regions.

saves_01.ps

DIII_Mutant_D_F1534L

PROCHECK

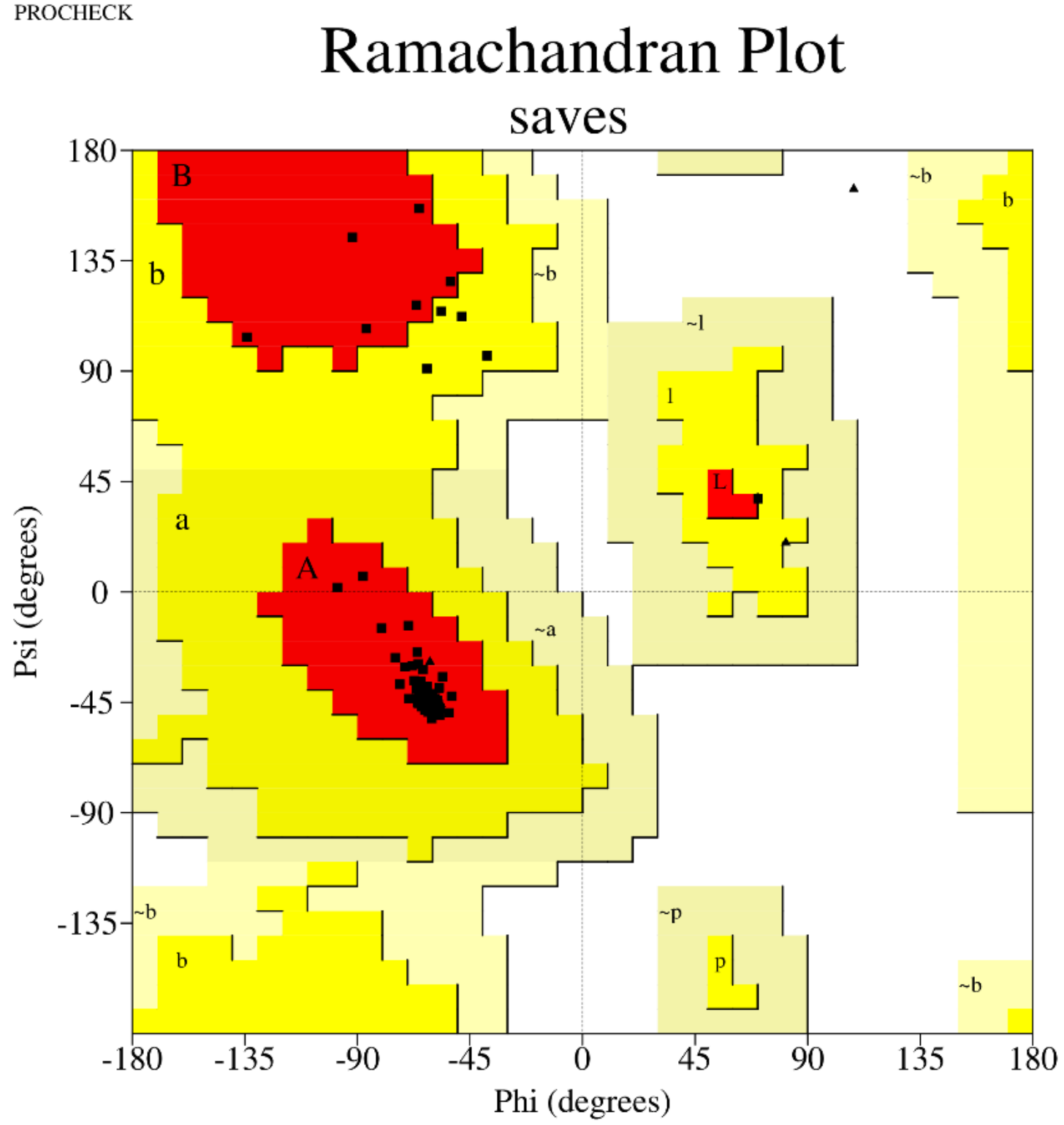

Plot statistics

Residues in most favoured regions [A,B,L]	61	93.8%
Residues in additional allowed regions [a,b,l,p]	4	6.2%
Residues in generously allowed regions [~a,~b,~l,~p]	0	0.0%
Residues in disallowed regions	0	0.0%
	----	------
Number of non-glycine and non-proline residues	65	100.0%
Number of end-residues (excl. Gly and Pro)	1	
Number of glycine residues (shown as triangles)	5	
Number of proline residues	2	

Total number of residues	73	

Based on an analysis of 118 structures of resolution of at least 2.0 Angstroms and R-factor no greater than 20%, a good quality model would be expected to have over 90% in the most favoured regions.

saves_01.ps

DIII_Mutant_E_P1585R

PROCHECK

Ramachandran Plot

saves

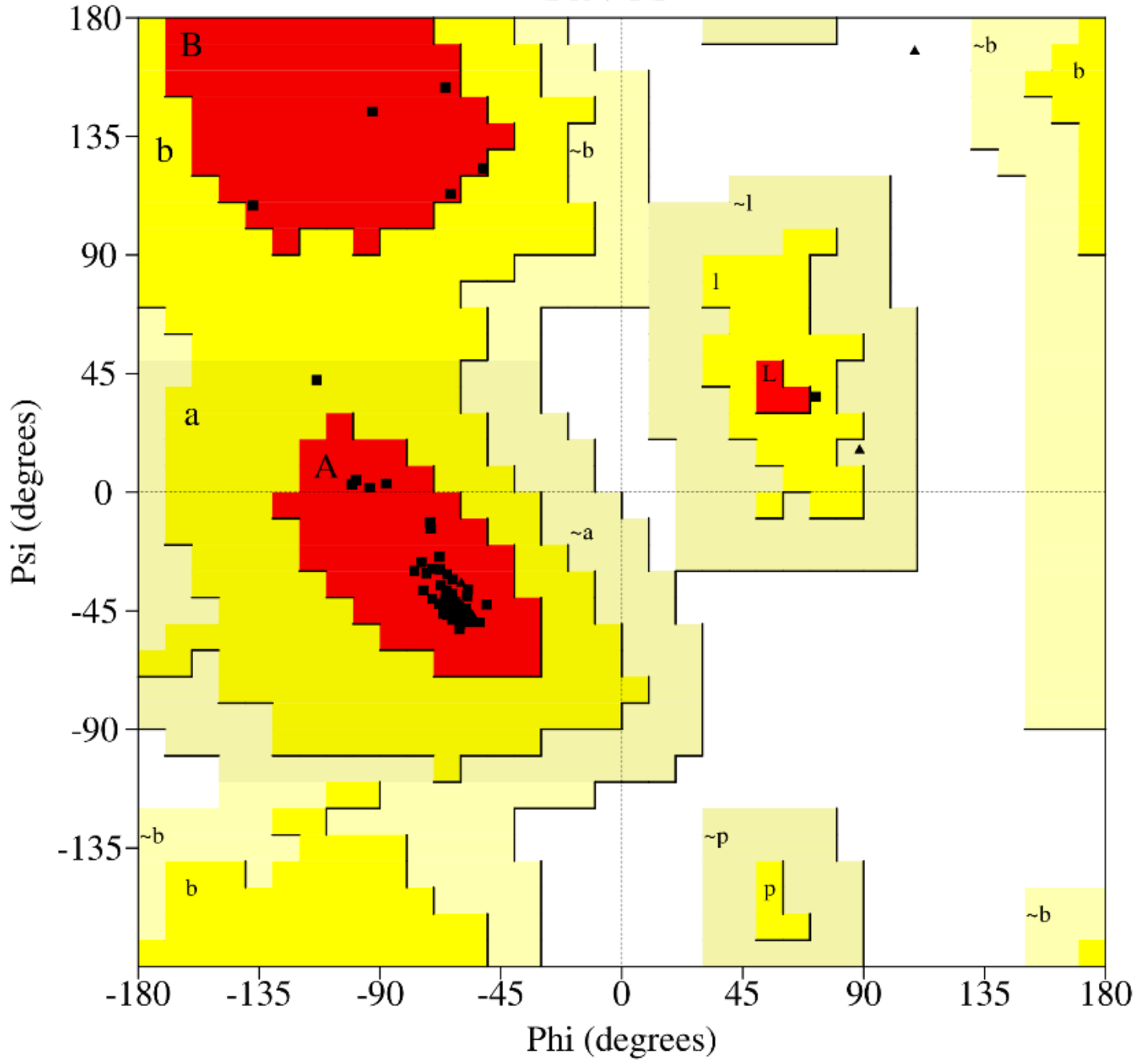

Plot statistics

Residues in most favoured regions [A,B,L]	64	97.0%
Residues in additional allowed regions [a,b,l,p]	2	3.0%
Residues in generously allowed regions [~a,~b,~l,~p]	0	0.0%
Residues in disallowed regions	0	0.0%
	----	------
Number of non-glycine and non-proline residues	66	100.0%
Number of end-residues (excl. Gly and Pro)	1	
Number of glycine residues (shown as triangles)	5	
Number of proline residues	1	

Total number of residues	73	

Based on an analysis of 118 structures of resolution of at least 2.0 Angstroms and R-factor no greater than 20%, a good quality model would be expected to have over 90% in the most favoured regions.

saves_01.ps

DIII_Mutant_D+E_F1524L+P1585R

PROCHECK

Ramachandran Plot

saves

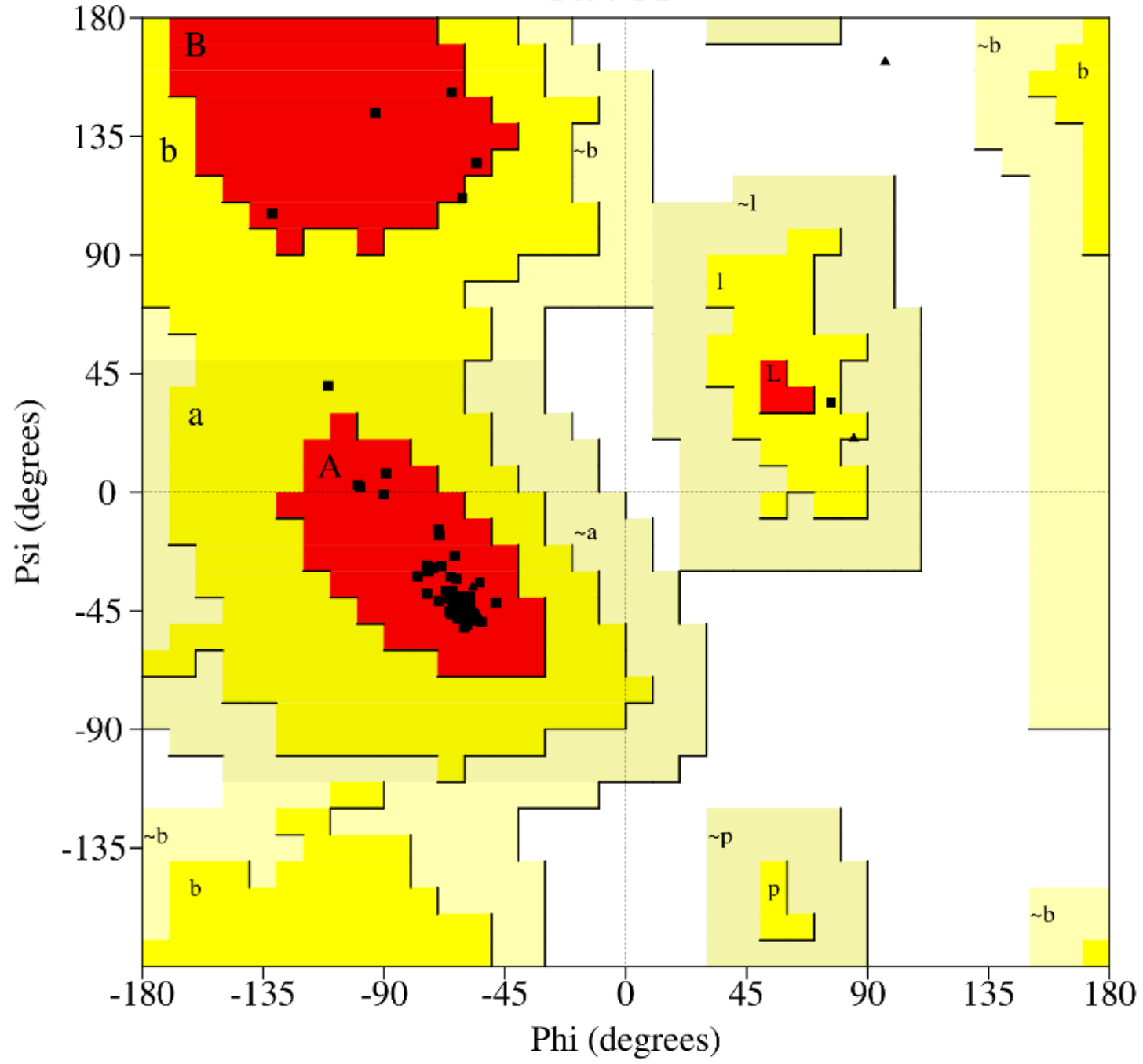

Plot statistics

Residues in most favoured regions [A,B,L]	64	97.0%
Residues in additional allowed regions [a,b,l,p]	2	3.0%
Residues in generously allowed regions [~a,~b,~l,~p]	0	0.0%
Residues in disallowed regions	0	0.0%
	----	------
Number of non-glycine and non-proline residues	66	100.0%
Number of end-residues (excl. Gly and Pro)	1	
Number of glycine residues (shown as triangles)	5	
Number of proline residues	1	

Total number of residues	73	

Based on an analysis of 118 structures of resolution of at least 2.0 Angstroms and R-factor no greater than 20%, a good quality model would be expected to have over 90% in the most favoured regions.

saves_01.ps

DIV_WildType_F169

PROCHECK

Ramachandran Plot

saves

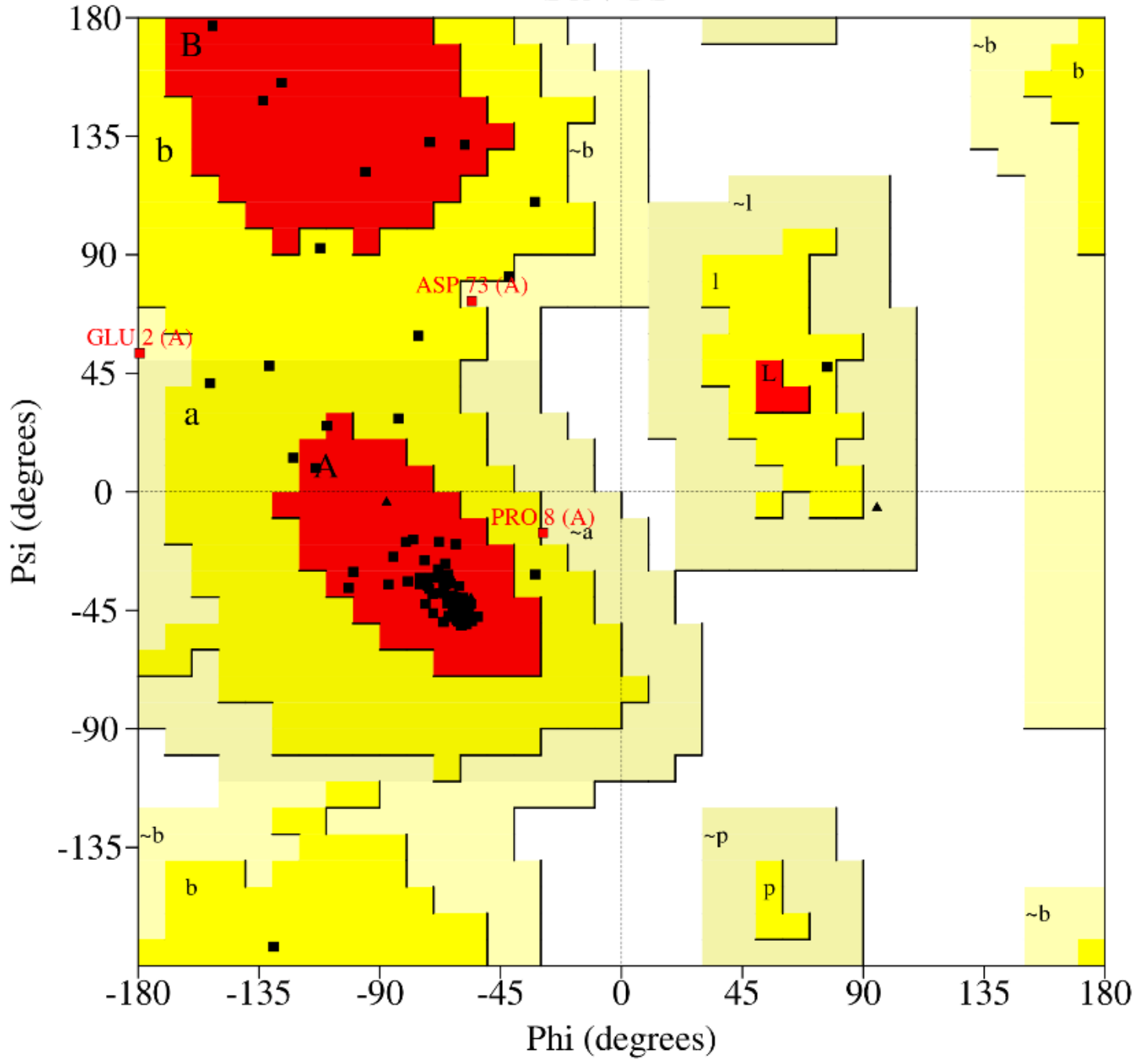

Plot statistics

Residues in most favoured regions [A,B,L]	71	84.5%
Residues in additional allowed regions [a,b,l,p]	11	13.1%
Residues in generously allowed regions [~a,~b,~l,~p]	2	2.4%
Residues in disallowed regions	0	0.0%
	----	------
Number of non-glycine and non-proline residues	84	100.0%
Number of end-residues (excl. Gly and Pro)	2	
Number of glycine residues (shown as triangles)	6	
Number of proline residues	2	

Total number of residues	94	

Based on an analysis of 118 structures of resolution of at least 2.0 Angstroms and R-factor no greater than 20%, a good quality model would be expected to have over 90% in the most favoured regions.

saves_01.ps

DIV_Mutant_F_F1695L

PROCHECK

Ramachandran Plot

saves

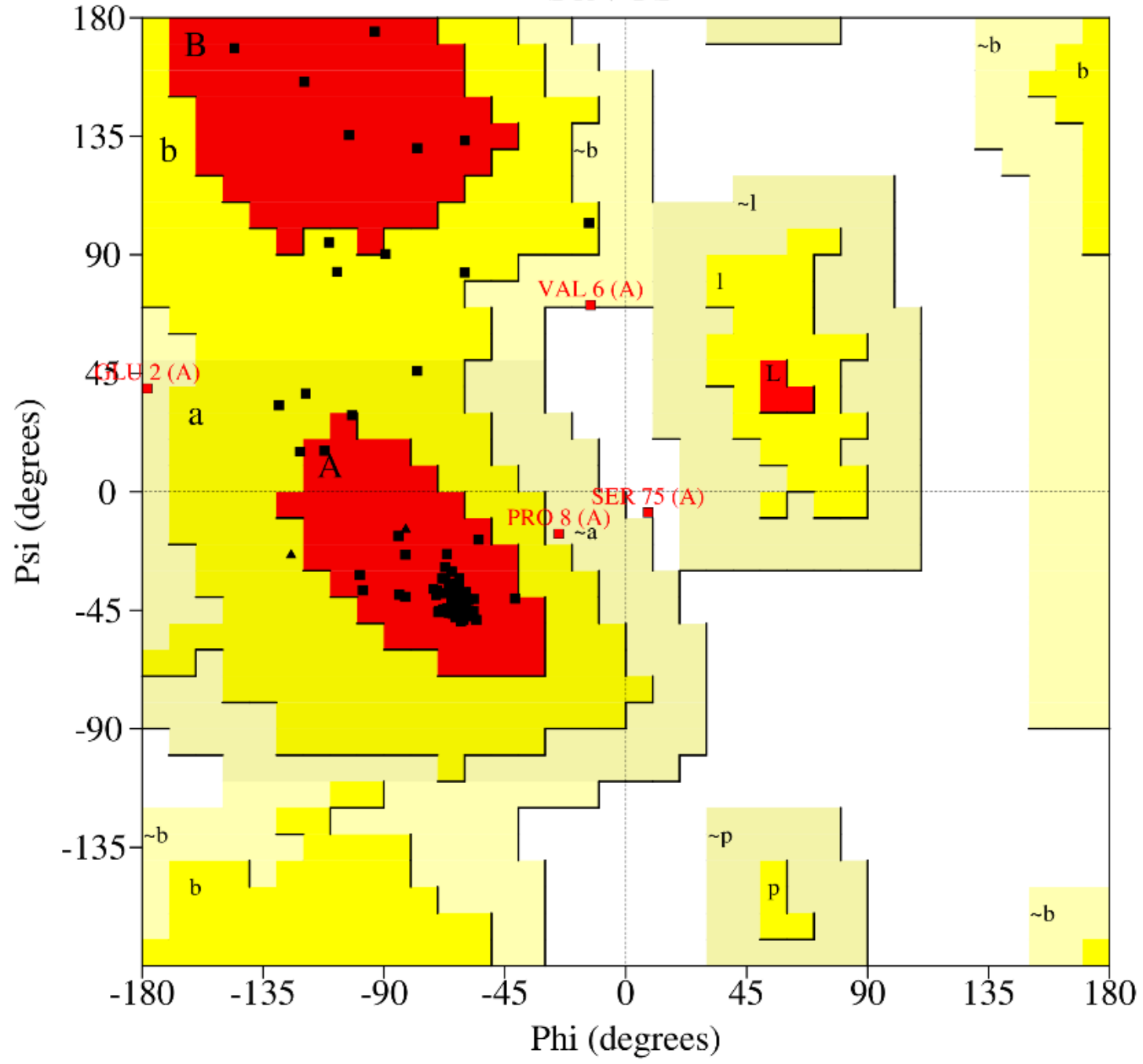

Plot statistics

Residues in most favoured regions [A,B,L]	72	85.7%
Residues in additional allowed regions [a,b,l,p]	9	10.7%
Residues in generously allowed regions [~a,~b,~l,~p]	2	2.4%
Residues in disallowed regions	1	1.2%
	----	------
Number of non-glycine and non-proline residues	84	100.0%
Number of end-residues (excl. Gly and Pro)	2	
Number of glycine residues (shown as triangles)	6	
Number of proline residues	2	

Total number of residues	94	

Based on an analysis of 118 structures of resolution of at least 2.0 Angstroms and R-factor no greater than 20%, a good quality model would be expected to have over 90% in the most favoured regions.

saves_01.ps
