# Supplementary material for: Emerging and regionally novel voltage-gated sodium channel mutations associated with pyrethroid resistance and altered binding affinity in Aedes albopictus (Skuse) from Northern Peninsular Malaysia
Source: Parasit Vectors. 2026 Jun 21;19:321. doi: 10.1186/s13071-026-07496-w (PMC13449526; doi:10.1186/s13071-026-07496-w)
Supplement: Supplementary file 3 [file 13071_2026_7496_MOESM3_ESM.pdf]

Additional file 3: Table S2: Percentage mortality of *Ae. albopictus* larvae at 24h post-treatment against diagnostic dose of 0.012 ppm and 0.034 ppm temephos larvicides, respectively.

State	Sample	Percentage of mortality (% ± SD)		Interpretation of susceptibility status (based on 0.034 ppm)
		0.012 ppm	0.034 ppm	
**Penang**	**USM VCRU**	100.00 ± 0.00 [S] (200)	100.00 ± 0.00 [S] (200)	Susceptible
	**SA**	40.50 ± 1.55 [R] (200)	65.67 ± 2.06 [R] (300)	Confirmed resistance
	**BP**	59.00 ± 1.98 [R] (200)	85.00 ± 3.14 [R] (300)	Confirmed resistance
**Perlis**	**KB**	5.67 ± 1.44 [R] (300)	61.33 ± 2.10 [R] (300)	Confirmed resistance
	**KTT**	9.33 ± 1.61 [R] (300)	80.00 ± 2.59 [R] (300)	Confirmed resistance

n = 200-300. The sample size was not standardized due to insufficient number of F1 mosquito samples. Percentage mortality after 24 hours = % ± SD; [S] = susceptible (98-100% mortality), [P] = possible resistance/incipient resistance, [R] = confirmed resistance (< 90% mortality), as determined by WHO (2016)

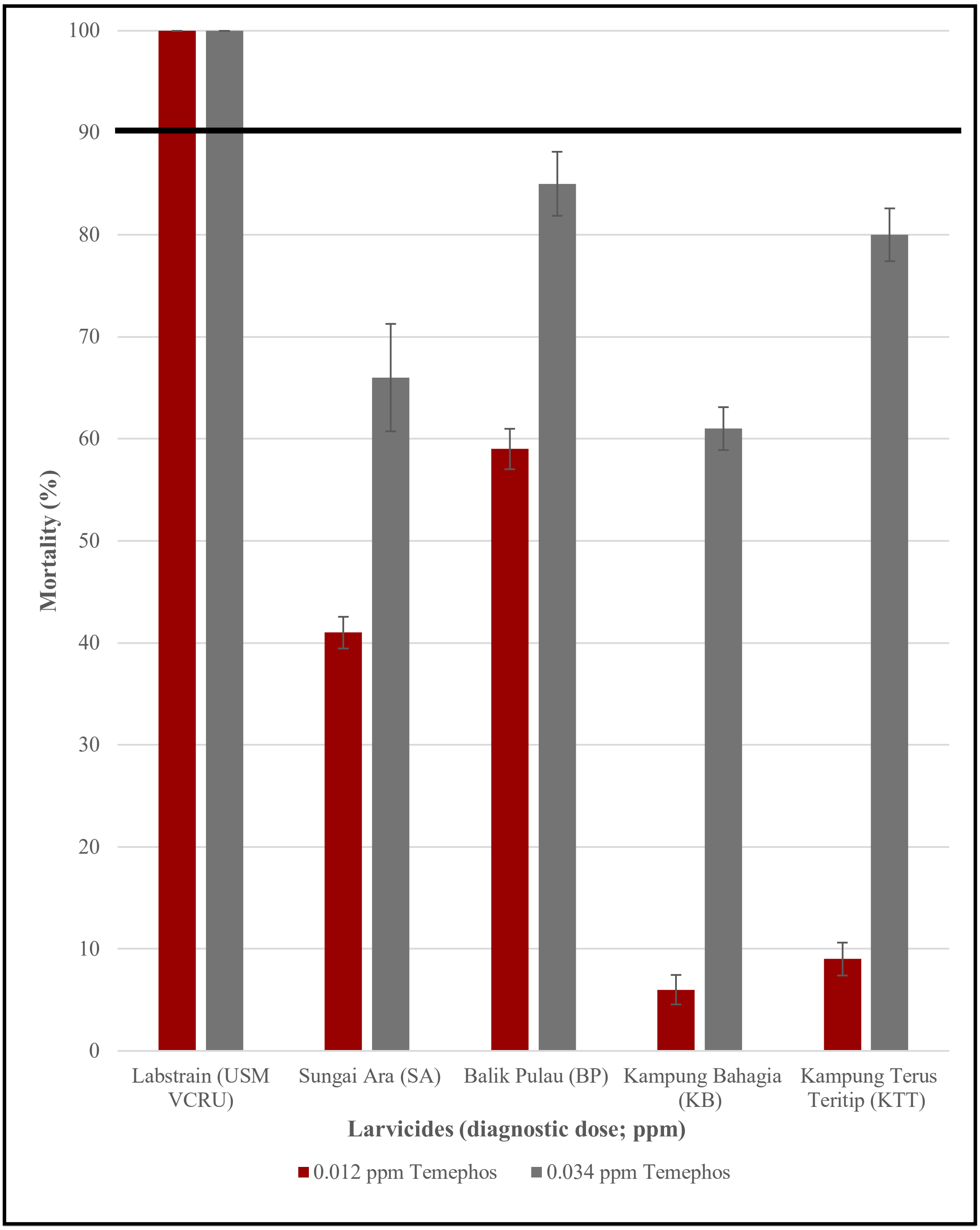

Additional file 3: Fig. S2: Percentage mortality of *Ae. albopictus* larvae from the different localities in Penang and Perlis, Malaysia towards 0.012 ppm and 0.034 ppm of temephos. Error bars represent 95% confidence interval (CI). Black horizontal line indicates resistant threshold level (mortality <90% is considered phenotypically resistant; WHO, 2016).

Additional file 3: Table S3: Summary of LC_{50}, LC_{95}, diagnostic dose (DD), and resistance ratios (RR) from larval bioassays of temephos in field strains of *Ae. albopictus* compared with the USM VCRU laboratory strain.

State	Sample	Sample size (n)	LC_{50}, ppm (95% CL)	LC_{95}, ppm (95% CL)	DD, ppm	RR_{50}, folds	RR_{95}, folds
**Penang**	**USM VCRU**	650	0.007 (0.006-0.008)[a]	0.013 (0.011-0.024)[a]	0.034	1	1
	**SA**	850	0.017 (0.014-0.02)[b]	0.049 (0.035-0.095)[b]	0.152	2.43	3.77
	**BP**	550	0.011 (0.010-0.11)[c]	0.025 (0.021-0.033)[a]	0.072	1.57	1.92
**Perlis**	**KB**	750	0.028 (0.024-0.032)[d]	0.056 (0.045-0.086)[b]	0.148	4	4.31
	**KTT**	750	0.024 (0.023-0.025)[d]	0.054 (0.048-0.063)[b]	0.152	3.43	4.15

All LC_{50} and LC_{99} are statistically significant using overlapping of confidence limits test. CL = Confidence limits; [a,b,c,d] Statistically significant using overlapping of confidence interval test. Susceptible: RR is <5; Moderate resistance: RR is 5-10; Highly resistance: RR is >10 as determined by WHO (2016).
