# Supplementary material for: Emerging and regionally novel voltage-gated sodium channel mutations associated with pyrethroid resistance and altered binding affinity in Aedes albopictus (Skuse) from Northern Peninsular Malaysia
Source: Parasit Vectors. 2026 Jun 21;19:321. doi: 10.1186/s13071-026-07496-w (PMC13449526; doi:10.1186/s13071-026-07496-w)
Supplement: Supplementary file 4 [file 13071_2026_7496_MOESM4_ESM.pdf]

Additional file 4: Table S4: Percentage mortality of females *Ae. albopictus* at 24h post-treatment against several insecticides from carbamate, organophosphate and pyrethroid classes, together with 4% PBO as a synergist for pyrethroid insecticides.

State	Sample	% Mortality ± SD					
		Adulticides					
		Carbamate	Organophosphate	Pyrethroid		Synergist	
		0.1% Propoxur	0.25% Pirimiphos methyl	0.25% Permethrin	0.03% Deltamethrin	4% PBO + 0.25% Permethrin	4% PBO + 0.03% Deltamethrin
**Penang**	**USM VCRU**	100.00 ± 0.00 [S] (300)	100.00 ± 0.00 [S] (300)	100.00 ± 0.00 [S] (300)	100.00 ± 0.00 [S] (300)	NA	NA
	**SA**	19.33 ± 2.25 [R] (300)	94.33 ± 1.08 [P] (300)	79.00 ± 4.22 [R] (300)	96.67 ± 0.99 [P] (300)	99.00 ± 0.46 (200)	100.00 ± 0.00 (200)
	**BP**	13.00 ± 4.86 [R] (200)	92.33 ± 0.99 [P] (300)	93.00 ± 1.60 [P] (300)	90.00 ± 2.11 [P] (300)	99.50 ± 0.35 (200)	100.00 ± 0.00 (200)
**Perlis**	**KB**	2.00 ± 1.07 [R] (200)	81.33 ± 3.39 [R] (300)	34.00 ± 2.02 [R] (300)	3.67 ± 1.24 [R] (300)	91.50 ± 2.17 (200)	12.50 ± 2.80 (200)
	**KTT**	9.50 ± 1.60 [R] (200)	70.00 ± 4.10 [R] (300)	48.67 ± 6.07 [R] (300)	10.50 ± 2.83 [R] (200)	97.00 ± 0.71 (200)	19.00 ± 1.83 (200)

n = 200-300. The sample size was not standardized due to insufficient number of F1 mosquito samples. Percentage mortality after 24 hours = % ± SD; [S] = susceptible (98-100% mortality), [P] = possible resistance/incipient resistance, [R] = confirmed resistance (< 90% mortality), as determined by WHO (2016).

Additional file 4: Table S5: Knockdown rates of *Ae. albopictus* in Penang and Perlis, Malaysia against pyrethroid adulticides.

State	Sample	Sample size (n)	Adulticides							
			Pyrethroid							
			0.25% Permethrin				0.03% Deltamethrin			
			KdT_{50} (min) (95% CL)	RR_{50}, folds	KdT_{95} (min) (95% CL)	RR_{95}, folds	KdT_{50} (min) (95% CL)	RR_{50}, folds	KdT_{95} (min) (95% CL)	RR_{95}, folds
**Penang**	**USM VCRU**	300	31.54 (30.19-32.88)[a]	1	56.68 (52.29-63.02)[a]	1	21.19 (20.71-21.68)[a]	1	33.28 (31.86-35.04)[a]	1
	**SA**	300	42.73 (41.67-43.85)[b]	1.35	94.06 (87.84-101.87)[b]	1.66	15.57 (12.99-18.13)[b]	0.73	30.65 (24.31-54.00)[a]	0.92
	**BP**	300	35.91 (35.07-36.74)[c]	1.14	72.89 (69.38-77.10)[c]	1.29	16.86 (15.36-18.41)[b]	0.79	28.22 (24.60-35.79)[a]	0.85
**Perlis**	**KB**	300	95.19 (81.91-121.19)[d]	3.02	378.38 (250.02-744.93)[d]	6.68	NA	NA	NA	NA
	**KTT**	300	73.02 (67.62-81.13)[e]	2.32	199.45 (159.78-273.48)[e]	3.52	768.86 (ND)	NA	7070.70 (ND)	NA

CL, confidence limit; [a,b,c,d,e] Statistically significant using overlapping of confidence interval test; KdT, knockdown time; NA, not available; ND, not determined by probit analysis because knockdown rate <5%, indicating high resistance. RR values of <5 = low resistance, 5–10 = medium resistance and >10 = high resistance
