# Supplementary material for: Emerging and regionally novel voltage-gated sodium channel mutations associated with pyrethroid resistance and altered binding affinity in Aedes albopictus (Skuse) from Northern Peninsular Malaysia
Source: Parasit Vectors. 2026 Jun 21;19:321. doi: 10.1186/s13071-026-07496-w (PMC13449526; doi:10.1186/s13071-026-07496-w)
Supplement: Supplementary file 5 [file 13071_2026_7496_MOESM6_ESM.pdf]

Additional file 6: Table S9: Individual-level genotype-phenotype associations between *vgsc* mutation burden and pyrethroid resistance in *Ae. albopictus*.

Strain	ID	Phenotype	Mutation_Count	Mutation_Combination	OR (CI)	*P*-value
USM_VCRU	USM_VCRU_Perm_1	Sus	0	None	NA	NA
USM_VCRU	USM_VCRU_Perm_2	Sus	0	None	NA	NA
USM_VCRU	USM_VCRU_Perm_3	Sus	0	None	NA	NA
USM_VCRU	USM_VCRU_Perm_4	Sus	0	None	NA	NA
USM_VCRU	USM_VCRU_Perm_5	Sus	0	None	NA	NA
USM_VCRU	USM_VCRU_Delta_1	Sus	0	None	NA	NA
USM_VCRU	USM_VCRU_Delta_2	Sus	0	None	NA	NA
USM_VCRU	USM_VCRU_Delta_3	Sus	0	None	NA	NA
USM_VCRU	USM_VCRU_Delta_4	Sus	0	None	NA	NA
USM_VCRU	USM_VCRU_Delta_5	Sus	0	None	NA	NA
SA	SA_Perm_1	Sus	0	None	NA	NA
SA	SA_Perm_2	Sus	0	None	NA	NA
SA	SA_Perm_3	Sus	1	A1022S	4.80 (1.53-17.05)	0.00292
SA	SA_Perm_4	Sus	0	None	NA	NA
SA	SA_Perm_5	Sus	1	None	NA	NA
SA	SA_Perm_1	Res	4	A1022P+E1041K+P1585R+F1695L	∞ (0.81-∞)	0.04006
SA	SA_Perm_2	Res	1	P1585R	4.80 (1.53-17.05)	0.00292
SA	SA_Perm_3	Res	2	F1534L+F1695L	8.15 (1.58-81.25)	0.00401
SA	SA_Perm_4	Res	2	A1022S+F1695L	8.15 (1.58-81.25)	0.00401
SA	SA_Perm_5	Res	2	F1534L+F1695L	8.15 (1.58-81.25)	0.00401
SA	SA_Delta_1	Sus	0	None	NA	NA
SA	SA_Delta_2	Sus	0	None	NA	NA
SA	SA_Delta_3	Sus	0	None	NA	NA
SA	SA_Delta_4	Sus	0	None	NA	NA
SA	SA_Delta_5	Sus	0	None	NA	NA
SA	SA_Delta_1	Res	2	A1022S+E1041K	8.15 (1.58-81.25)	0.00401
SA	SA_Delta_2	Res	3	A1022S+E1041K+P1585R	∞ (0.24-∞)	0.19476
SA	SA_Delta_3	Res	3	A1022S+E1041K+P1585R	∞ (0.24-∞)	1.19476
SA	SA_Delta_4	Res	2	F1534L+F1695L	8.15 (1.58-81.25)	0.00401
SA	SA_Delta_5	Res	1	A1022S	4.80 (1.53-17.05)	0.00292
BP	BP_Perm_1	Sus	0	None	NA	NA
BP	BP_Perm_2	Sus	0	None	NA	NA
BP	BP_Perm_3	Sus	0	None	NA	NA

BP	BP_Perm_4	Sus	0	None	NA	NA
BP	BP_Perm_5	Sus	0	None	NA	NA
BP	BP_Perm_1	Res	2	A1022S+E1041K	8.15 (1.58-81.25)	0.00401
BP	BP_Perm_2	Res	0	None	NA	NA
BP	BP_Perm_3	Res	1	P1585R	4.80 (1.53-17.05)	0.00292
BP	BP_Perm_4	Res	1	F1695L	4.80 (1.53-17.05)	0.00292
BP	BP_Perm_5	Res	1	F1695L	4.80 (1.53-17.05)	0.00292
BP	BP_Delta_1	Sus	0	None	NA	NA
BP	BP_Delta_2	Sus	0	None	NA	NA
BP	BP_Delta_3	Sus	0	None	NA	NA
BP	BP_Delta_4	Sus	0	None	NA	NA
BP	BP_Delta_5	Sus	0	None	NA	NA
BP	BP_Delta_1	Res	0	None	NA	NA
BP	BP_Delta_2	Res	1	P1585R	4.80 (1.53-17.05)	0.00292
BP	BP_Delta_3	Res	1	A1022P	4.80 (1.53-17.05)	0.00292
BP	BP_Delta_4	Res	0	None	NA	NA
BP	BP_Delta_5	Res	2	A1022P + F1695L	8.15 (1.58-81.25)	0.00401
KB	KB_Perm_1	Sus	0	None	NA	NA
KB	KB_Perm_2	Sus	1	F1534L	4.80 (1.53-17.05)	0.00292
KB	KB_Perm_3	Sus	1	F1534L	4.80 (1.53-17.05)	0.00292
KB	KB_Perm_4	Sus	0	None	NA	NA
KB	KB_Perm_5	Sus	2	A1022S+P1585R	8.15 (1.58-81.25)	0.00401
KB	KB_Perm_1	Res	2	F1534L+F1695L	8.15 (1.58-81.25)	0.00401
KB	KB_Perm_2	Res	1	F1534L	4.80 (1.53-17.05)	0.00292
KB	KB_Perm_3	Res	2	F1534L+P1585R	8.15 (1.58-81.25)	0.00401
KB	KB_Perm_4	Res	0	None	NA	NA
KB	KB_Perm_5	Res	1	F1534L	4.80 (1.53-17.05)	0.00292
KB	KB_Delta_1	Sus	0	None	NA	NA
KB	KB_Delta_2	Sus	1	F1534L	4.80 (1.53-17.05)	0.00292
KB	KB_Delta_3	Sus	0	None	NA	NA
KB	KB_Delta_4	Sus	0	None	NA	NA
KB	KB_Delta_5	Sus	0	None	NA	NA
KB	KB_Delta_1	Res	1	F1534L	4.80 (1.53-17.05)	0.00292
KB	KB_Delta_2	Res	4	A1022S+E1041K+F1534L+P1585R	∞ (0.81-∞)	0.04006
KB	KB_Delta_3	Res	0	None	NA	NA

KB	KB_Delta_4	Res	1	F1695L	4.80 (1.53-17.05)	0.00292
KB	KB_Delta_5	Res	1	F1534L	4.80 (1.53-17.05)	0.00292
KTT	KTT_Perm_1	Sus	0	None	NA	NA
KTT	KTT_Perm_2	Sus	0	None	NA	NA
KTT	KTT_Perm_3	Sus	0	None	NA	NA
KTT	KTT_Perm_4	Sus	1	P1585R	4.80 (1.53-17.05)	0.00292
KTT	KTT_Perm_5	Sus	2	P1585R	4.80 (1.53-17.05)	0.00292
KTT	KTT_Perm_1	Res	0	None	NA	NA
KTT	KTT_Perm_2	Res	1	A1022S	4.80 (1.53-17.05)	0.00292
KTT	KTT_Perm_3	Res	0	None	NA	NA
KTT	KTT_Perm_4	Res	2	F1534L+F1695L	8.15 (1.58-81.25)	0.00401
KTT	KTT_Perm_5	Res	1	P1585R	4.80 (1.53-17.05)	0.00292
KTT	KTT_Delta_1	Sus	0	None	NA	NA
KTT	KTT_Delta_2	Sus	0	None	NA	NA
KTT	KTT_Delta_3	Sus	0	None	NA	NA
KTT	KTT_Delta_4	Sus	0	None	NA	NA
KTT	KTT_Delta_5	Sus	0	None	NA	NA
KTT	KTT_Delta_1	Res	1	F1534L	4.80 (1.53-17.05)	0.00292
KTT	KTT_Delta_2	Res	0	None	NA	NA
KTT	KTT_Delta_3	Res	1	P1585R	4.80 (1.53-17.05)	0.00292
KTT	KTT_Delta_4	Res	0	None	NA	NA
KTT	KTT_Delta_5	Res	4	A1022S+E1041K+F1534L+P1585R	∞ (0.81-∞)	0.04006

Range of odd-ratio (OR): OR = 1 (No association between genotype and phenotype), OR <1 (Negative association, or low likelihood of insecticide resistance), OR >1 (Positive association, or high likelihood of insecticide resistance), OR = ∞ reflects an unbounded odds ratio due to zero susceptible individuals observed in the corresponding genotype category; a 0.5 continuity (Haldane-Anscombe) correction was applied. Odds ratios are presented with 95% confidence intervals (95% CI), which may be wide in sparse data conditions. Statistical analysis: Fisher's exact test was used due to small sample sizes; $P < 0.05$ indicates statistical significance. Mutation burden was defined as the total number of detected *vgsc* mutations per mosquito, irrespective of zygosity. "NA" indicates that ORs were not estimated for wild-type individuals or categories with no mutation. Note: Estimates involving zero or low cell counts should be interpreted cautiously, as sparse data may result in wide confidence intervals and limited statistical power.

Additional file 6: Table S10(A): Association of genotype A1022S with the resistance phenotype towards pyrethroid insecticides for the Penang and Perlis strains of *Ae. albopictus.*

Population	Insecticide	Phenotype	n	A1022S					Mutant alleles frequency	Odd- ratio (OR)	Fisher's Exact test (*P*-value)
				Genotype			Allele				
				A/A	A/S	S/S	GCC	TCC			
SA	Permethrin	Sus	5	4	1	0	9	1	0.10	1.33	0.582
		Res	5	4	1	0	9	1	0.20		
	Deltamethrin	Sus	5	5	0	0	10	0	0.00	15.40	0.087
		Res	5	1	4	0	6	4	0.40		
BP	Permethrin	Sus	5	5	0	0	9	1	0.00	3.67	1.000
		Res	5	4	1	0	9	1	0.10		
	Deltamethrin	Sus	5	5	0	0	10	0	0.00	1.00	1.000
		Res	5	5	0	0	10	0	0.00		
KB	Permethrin	Sus	5	4	0	1	8	2	0.20	0.27	0. 474
		Res	5	5	0	0	10	0	0.00		
	Deltamethrin	Sus	5	5	0	0	10	0	0.00	1.22	0.474
		Res	5	4	0	1	8	2	0.10		
KTT	Permethrin	Sus	5	5	0	0	10	0	0.00	3.67	1.000
		Res	5	4	1	0	9	1	0.10		
	Deltamethrin	Sus	5	5	0	0	10	0	0.00	1.22	0.474
		Res	5	4	0	1	8	2	0.10		

Odds ratios (OR) were calculated using a 0.5 continuity (Haldane-Anscombe) correction to account for zero counts in small sample sizes. OR = 1 indicates no association between genotype and phenotype; OR < 1 indicates a negative association (reduced likelihood of insecticide resistance); and OR > 1 indicates a positive association (increased likelihood of insecticide resistance). Statistical significance was assessed using Fisher's exact test, with $P < 0.05$ considered significant.

Additional file 6: Table S10(B): Association of genotype A1022P with the resistance phenotype towards pyrethroid insecticides for the Penang and Perlis strains of *Ae. albopictus.*

Population	Insecticide	Phenotype	n	A1022P					Mutant alleles frequency	Odd- ratio (OR)	Fisher's Exact test (*P* -value)
				Genotype			Allele				
				A/A	A/P	P/P	GCC	CCC			
SA	Permethrin	Sus	5	5	0	0	10	0	0.00	0.33	0.242
		Res	5	4	0	1	8	2	0.20		
	Deltamethrin	Sus	5	5	0	0	10	0	0.00	1.00	1.000
		Res	5	5	0	0	10	0	0.00		
BP	Permethrin	Sus	5	5	0	0	10	0	0.00	1.00	1.000
		Res	5	5	0	0	10	0	0.00		
	Deltamethrin	Sus	5	5	0	0	10	0	0.00	0.75	0.667
		Res	5	3	0	2	6	4	0.40		
KB	Permethrin	Sus	5	5	0	0	10	0	0.00	1.00	1.000
		Res	5	5	0	0	10	0	0.00		
	Deltamethrin	Sus	5	5	0	0	10	0	0.00	1.00	1.000
		Res	5	5	0	0	10	0	0.00		
KTT	Permethrin	Sus	5	5	0	0	10	0	0.00	1.00	1.000
		Res	5	5	0	0	10	0	0.00		
	Deltamethrin	Sus	5	5	0	0	10	0	0.00	1.00	1.000
		Res	5	5	0	0	10	0	0.00		

Odds ratios (OR) were calculated using a 0.5 continuity (Haldane-Anscombe) correction to account for zero counts in small sample sizes. OR = 1 indicates no association between genotype and phenotype; OR < 1 indicates a negative association (reduced likelihood of insecticide resistance); and OR > 1 indicates a positive association (increased likelihood of insecticide resistance). Statistical significance was assessed using Fisher's exact test, with *P* < 0.05 considered significant.

Additional file 6: Table S10(C): Association of genotype E1014K with the resistance phenotype towards pyrethroid insecticides for the Penang and Perlis strains of *Ae. albopictus.*

Population	Insecticide	Phenotype	n	E1041K					Mutant alleles frequency	Odd- ratio (OR)	Fisher's Exact test (*P* -value)
				Genotype			Allele				
				E/E	E/K	K/K	GAA	AAA			
SA	Permethrin	Sus	5	5	0	0	10	0	0.00	6.18	0.474
		Res	5	4	0	1	8	2	0.20		
	Deltamethrin	Sus	5	5	0	0	10	0	0.00	15.40	0.048*
		Res	5	2	0	3	4	6	0.60		
BP	Permethrin	Sus	5	5	0	0	10	0	0.00	6.18	0.474
		Res	5	4	0	1	8	2	0.20		
	Deltamethrin	Sus	5	5	0	0	10	0	0.00	1.00	1.000
		Res	5	5	0	0	10	0	0.00		
KB	Permethrin	Sus	5	5	0	0	10	0	0.00	1.00	1.000
		Res	5	5	0	0	10	0	0.00		
	Deltamethrin	Sus	5	5	0	0	10	0	0.00	6.18	0.474
		Res	5	4	0	1	8	2	0.20		
KTT	Permethrin	Sus	5	5	0	0	10	0	0.00	1.00	1.000
		Res	5	5	0	0	10	0	0.00		
	Deltamethrin	Sus	5	5	0	0	10	0	0.00	6.18	0.474
		Res	5	4	0	1	8	2	0.20		

Odds ratios (OR) were calculated using a 0.5 continuity (Haldane-Anscombe) correction to account for zero counts in small sample sizes. OR = 1 indicates no association between genotype and phenotype; OR < 1 indicates a negative association (reduced likelihood of insecticide resistance); and OR > 1 indicates a positive association (increased likelihood of insecticide resistance). Statistical significance was assessed using Fisher's exact test, with $P < 0.05$ considered significant.

Additional file 6: Table S10(D): Association of genotype F1534L with the resistance phenotype towards pyrethroid insecticides for the Penang and Perlis strains of *Ae. albopictus.*

Population	Insecticide	Phenotype	n	F1534L					Mutant alleles frequency	Odd- ratio (OR)	Fisher's Exact test (*P* -value)
				Genotype			Allele				
				F/F	F/L	L/L	TT<u>C</u>	TT<u>G</u>			
SA	Permethrin	Sus	5	5	0	0	10	0	0.00	7.86	0.444
		Res	5	3	2	0	8	2	0.20		
	Deltamethrin	Sus	5	5	0	0	10	0	0.00	1.00	1.000
		Res	5	5	0	0	10	0	0.00		
BP	Permethrin	Sus	5	5	0	0	10	0	0.00	1.00	1.000
		Res	5	5	0	0	10	0	0.00		
	Deltamethrin	Sus	5	5	0	0	10	0	0.00	1.00	1.000
		Res	5	5	0	0	10	0	0.00		
KB	Permethrin	Sus	5	3	2	0	8	2	0.20	6.00	0.524
		Res	5	1	4	0	6	4	0.40		
	Deltamethrin	Sus	5	4	1	0	9	1	0.10	6.00	0.524
		Res	5	2	3	0	7	3	0.30		
KTT	Permethrin	Sus	5	5	0	0	10	0	0.00	3.67	1.000
		Res	5	4	1	0	9	1	0.10		
	Deltamethrin	Sus	5	5	0	0	10	0	0.00	7.86	0.444
		Res	5	3	2	0	8	2	0.20		

Odds ratios (OR) were calculated using a 0.5 continuity (Haldane-Anscombe) correction to account for zero counts in small sample sizes. OR = 1 indicates no association between genotype and phenotype; OR < 1 indicates a negative association (reduced likelihood of insecticide resistance); and OR > 1 indicates a positive association (increased likelihood of insecticide resistance). Statistical significance was assessed using Fisher's exact test, with $P < 0.05$ considered significant.

Additional file 6: Table S10(E): Association of genotype P1585R with the resistance phenotype towards pyrethroid insecticides for the Penang and Perlis strains of *Ae. albopictus.*

Population	Insecticide	Phenotype	n	P1585R						Mutant alleles frequency	Odd- ratio (OR)	Fisher's Exact test (*P* -value)
				Genotype			Allele					
				P/P	P/R	R/R	CCG	CGG	CGC			
SA	Permethrin	Sus	5	5	0	0	10	0	0	0.00	7.86	0.087
		Res	5	3	0	2	6	4	0	0.40		
	Deltamethrin	Sus	5	5	0	0	10	0	0	0.00	15.40	0.011*
		Res	5	2	0	3	4	6	0	0.60		
BP	Permethrin	Sus	5	5	0	0	10	0	0	0.00	6.18	0.474
		Res	5	4	0	1	8	2	0	0.20		
	Deltamethrin	Sus	5	5	0	0	10	0	0	0.00	6.18	0.474
		Res	5	4	0	1	8	2	0	0.20		
KB	Permethrin	Sus	5	4	0	1	8	2	0	0.20	1.00	1.000
		Res	5	4	0	1	8	2	0	0.20		
	Deltamethrin	Sus	5	5	0	0	10	0	0	0.00	6.18	0.474
		Res	5	4	0	1	8	2	0	0.20		
KTT	Permethrin	Sus	5	3	0	2	6	4	0	0.40	0.82	0.629
		Res	5	4	0	1	8	0	2	0.20		
	Deltamethrin	Sus	5	5	0	0	10	0	0	0.00	7.86	0.087
		Res	5	3	0	2	6	2	2	0.40		

Odds ratios (OR) were calculated using a 0.5 continuity (Haldane-Anscombe) correction to account for zero counts in small sample sizes. OR = 1 indicates no association between genotype and phenotype; OR < 1 indicates a negative association (reduced likelihood of insecticide resistance); and OR > 1 indicates a positive association (increased likelihood of insecticide resistance). Statistical significance was assessed using Fisher's exact test, with $P < 0.05$ considered significant.

Additional file 6: Table S10(F): Association of genotype F1695L with the resistance phenotype towards pyrethroid insecticides for the Penang and Perlis strains of *Ae. albopictus.*

Population	Insecticide	Phenotype	n	F1695L					Mutant alleles frequency	Odd-ratio (OR)	Fisher's Exact test (*P*-value)
				Genotype			Allele				
				F/F	F/L	L/L	TTC	CTC			
SA	Permethrin	Sus	5	5	0	0	10	0	0.00	33.00	0.048*
		Res	5	1	0	4	2	8	0.80		
	Deltamethrin	Sus	5	5	0	0	10	0	0.00	6.18	0.477
		Res	5	4	0	1	8	2	0.20		
BP	Permethrin	Sus	5	5	0	0	10	0	0.00	7.86	0.087
		Res	5	3	0	2	6	4	0.40		
	Deltamethrin	Sus	5	5	0	0	10	0	0.00	6.18	0.477
		Res	5	4	0	1	8	2	0.20		
KB	Permethrin	Sus	5	5	0	0	10	0	0.00	6.18	0.477
		Res	5	4	0	1	8	2	0.20		
	Deltamethrin	Sus	5	5	0	0	10	0	0.00	6.18	0.477
		Res	5	4	0	1	8	2	0.20		
KTT	Permethrin	Sus	5	5	0	0	10	0	0.00	6.18	0.477
		Res	5	4	0	1	8	2	0.20		
	Deltamethrin	Sus	5	5	0	0	10	0	0.00	1.00	1.000
		Res	5	5	0	0	10	0	0.00		

Odds ratios (OR) were calculated using a 0.5 continuity (Haldane-Anscombe) correction to account for zero counts in small sample sizes. OR = 1 indicates no association between genotype and phenotype; OR < 1 indicates a negative association (reduced likelihood of insecticide resistance); and OR > 1 indicates a positive association (increased likelihood of insecticide resistance). Statistical significance was assessed using Fisher's exact test, with $P < 0.05$ considered significant.
