# Supplementary material for: Emerging and regionally novel voltage-gated sodium channel mutations associated with pyrethroid resistance and altered binding affinity in Aedes albopictus (Skuse) from Northern Peninsular Malaysia
Source: Parasit Vectors. 2026 Jun 21;19:321. doi: 10.1186/s13071-026-07496-w (PMC13449526; doi:10.1186/s13071-026-07496-w)

Additional file 8: Table 13(A): Forty-one haplotypes were identified from 90 nucleotide sequences of DIIS6 in *vgsc* gene fragment of local *Ae. albopictus* in Penang and Perlis, and deposited in the NCBI Genbank.

*Haplotype	Accession no.	**No of sequences	Individual/sequence identification	Alleles		
				Codon 1016	Codon 1022	Codon 1041
H1	PQ660263	13	USM VCRU_Perm_Sus_1, USM VCRU _Perm_Sus_3, USM VCRU _Perm_Sus_4, USM VCRU _Perm_Sus_5, SA_Delta_Sus_2, BP_Perm_Sus_2, BP_Perm_Sus_5, BP_Delta_Sus_1, BP_Delta_Sus_3, BP_Delta_Sus_4, KTT_Perm_Res_1, KTT_Perm_Res_4, KTT_Delta_Sus_3	V/GTA	A/GCC	E/GAA
H2	PQ660264	6	USM VCRU_Perm_Sus_2, KB_Perm_Sus_2, KB_Perm_Sus_4, KB_Delta_Sus_3, KB_Delta_Sus_5, KB_Delta_Res_5, KTT_Perm_Sus_4	V/GTG	A/GCC	E/GAA
H3*	PQ660265	15	USM VCRU_Delta_Sus_1, USM VCRU_Delta_Sus_2, USM VCRU_Delta_Sus_3, USM VCRU_Delta_Sus_4, USM VCRU_Delta_Sus_5, BP_Perm_Sus_1, BP_Perm_Res_3, KB_Perm_Res_5, KB_Delta_Res_1, KB_Delta_Res_3, KB_Delta_Res_4, KTT_Perm_Res_5, KTT_Delta_Res_3, KTT_Delta_Res_4	V/GTA	A/GCC	E/GAA

H4	PQ660266	4	SA_Perm_Sus_1, SA_Perm_Sus_5, BP_Perm_Sus_3, BP_Perm_Res_2	V/GTA	A/GCC	E/GAA
H5	PQ660267	2	SA_Perm_Sus_2, SA_Delta_Sus_3	V/GTA	A/GCC	E/GAA
**H6**	PQ660268	1	SA_Perm_Sus_3	V/GTA	S/TCC	E/GAA
H7	PQ660269	1	SA_Perm_Sus_4	V/GTA	A/GCC	E/GAA
**H8****	PQ660270	1	SA_Perm_Res_1	V/GTA	P/CCC	K/AAA
H9	PQ660271	8	SA_Perm_Res_2, BP_Delta_Sus_2, KB_Delta_Sus_2, KTT_Perm_Sus_2, KTT_Perm_Sus_5, KTT_Delta_Sus_1, KTT_Delta_Sus_4, KTT_Delta_Sus_5	V/GTA	A/GCC	E/GAA
H10	PQ660272	1	SA_Perm_Res_3	V/GTA	A/GCC	E/GAA
H11	PQ660273	1	SA_Perm_Res_4	V/GTA	S/TCC	E/GAA
H12	PQ660274	1	SA_Perm_Res_5	V/GTA	A/GCC	E/GAA
H13	PQ660275	1	SA_Delta_Sus_1	V/GTA	A/GCC	E/GAA
H14	PQ660276	1	SA_Delta_Sus_4	V/GTA	A/GCC	E/GAA
H15	PQ660277	1	SA_Delta_Sus_5	V/GTA	A/GCC	E/GAA
**H16****	PQ660278	1	SA_Delta_Res_1	V/GTA	S/TCC	K/AAA
**H17****	PQ660279	2	SA_Delta_Res_2, BP_Perm_Res_1	V/GTA	S/TCC	K/AAA
**H18****	PQ660280	1	SA_Delta_Res_3	V/GTA	S/TCC	K/AAA
H19	PQ660281	1	SA_Delta_Res_4	V/GTA	A/GCC	E/GAA
**H20**	PQ660282	1	SA_Delta_Res_5	V/GTA	S/TCC	E/GAA
H21	PQ660283	1	BP_Perm_Sus_4	V/GTA	A/GCC	E/GAA
H22	PQ660284	1	BP_Perm_Res_4	V/GTA	A/GCC	E/GAA
H23	PQ660285	1	BP_Perm_Res_5	V/GTA	A/GCC	E/GAA
H24	PQ660286	1	BP_Delta_Sus_5	V/GTA	A/GCC	E/GAA
H25	PQ660287	2	BP_Delta_Res_1, KB_Delta_Sus_1	V/GTA	A/GCC	E/GAA
H26	PQ660288	1	BP_Delta_Res_2	V/GTA	A/GCC	E/GAA

**H27**	PQ660289	1	BP_Delta_Res_3	V/GTA	P/CCC	E/GAA
H28	PQ660290	1	BP_Delta_Res_4	V/GTA	A/GCC	E/GAA
**H29**	PQ660291	1	BP_Delta_Res_5	V/GTA	P/CCC	E/GAA
H30	PQ660292	1	KB_Perm_Sus_1	V/GTG	A/GCC	E/GAA
**H31**	PQ660293	1	KB_Perm_Sus_3	V/GTA	P/CCC	E/GAA
**H32**	PQ660294		KB_Perm_Sus_5	V/GTG	S/TCC	E/GAA
H33	PQ660295	4	KB_Perm_Res_1, KB_Perm_Res_2, KB_Perm_Res_3, KB_Perm_Res_4	V/GTA	A/GCC	E/GAA
H34	PQ660296	3	KB_Delta_Sus_4, KTT_Perm_Sus_1, KTT_Delta_Sus_2	V/GTA	A/GCC	E/GAA
**H35****	PQ660297	1	KB_Delta_Res_2	V/GTA	S/TCC	K/AAA
H36	PQ660298	1	KTT_Perm_Sus_3	V/GTA	A/GCC	E/GAA
**H37**	PQ660299	1	KTT_Perm_Res_2	V/GTA	S/TCC	E/GAA
H38	PQ660300	1	KTT_Perm_Res_3	V/GTA	A/GCC	E/GAA
H39	PQ660301	1	KTT_Delta_Res_1	V/GTA	A/GCC	E/GAA
H40	PQ660302	1	KTT_Delta_Res_2	V/GTA	A/GCC	E/GAA
**H41****	PQ660303	1	KTT_Delta_Res_5	V/GTA	S/TCC	K/AAA

*Number of sequences per haplotype. *Largest haplotype group with 15 homologous sequences. **Bold**: Haplotype with mutant alleles conferring knockdown or resistance phenotype. **Bold****: Haplotype with dual mutations.

Additional file 8: Table 13(B): Twenty-five haplotypes were identified from 90 nucleotide sequences of DIIIS6 in *vgsc* gene fragment of local *Ae. albopictus* in Penang and Perlis, and deposited in the NCBI Genbank.

*Haplotype	Accession no.	**No of sequences	Individual/sequence identification	Alleles		
				Codon 1532	Codon 1534	Codon 1585
H1	PQ660304	7	USM VCRU_Perm_Sus_1, USM VCRU _Perm_Sus_2, USM VCRU _Perm_Sus_4, USM VCRU _Delta_Sus_4, SA_Perm_Sus_2, BP_Perm_Sus_1, KB_Perm_Sus_1	I/ATC	F/TTC	P/CCG
H2*	PQ660305	28	USM VCRU_Perm_Sus_3, USM VCRU _Delta_Sus_3, USM VCRU _Delta_Sus_5, SA_Perm_Sus_3, SA_Perm_Sus_4, SA_Perm_Sus_5, SA_Perm_Res_4, SA_Delta_Sus_2, SA_Delta_Sus_3, SA_Delta_Sus_4, SA_Delta_Sus_5, SA_Delta_Res_5, BP_Perm_Sus_2, BP_Perm_Sus_4, BP_Perm_Res_2, BP_Perm_Res_4, BP_Delta_Sus_1, BP_Delta_Sus_3, BP_Delta_Sus_4, BP_Delta_Res_5, KB_Delta_Sus_5, KB_Delta_Res_4, KTT_Perm_Sus_2, KTT_Perm_Sus_3, KTT_Perm_Res_3, KTT_Delta_Sus_5, KTT_Delta_Res_2, KTT_Delta_Res_4	I/ATC	F/TTC	P/CCG
H3	PQ660306	11	USM VCRU_Perm_Sus_5, USM VCRU_Delta_Sus_1, BP_Perm_Sus_5, BP_Perm_Res_1, KB_Perm_Sus_4,	I/ATC	F/TTC	P/CCG

			KB_Delta_Sus_4, KTT_Perm_Res_1, KTT_Delta_Sus_1, KTT_Delta_Sus_2, KTT_Delta_Sus_3, KTT_Delta_Sus_4			
H4	PQ660307	1	USM VCRU_Delta_Sus_2	I/ATC	F/TTC	P/CCG
H5	PQ660308	1	SA_Perm_Sus_1	I/ATC	F/TTC	P/CCG
**H6**	PQ660309	6	SA_Perm_Res_1, SA_Perm_Res_2, SA_Delta_Res_2, SA_Delta_Res_3, SA_Delta_Res_4, KTT_Perm_Sus_5	I/ATC	F/TTC	R/CGG
**H7**	PQ660310	7	SA_Perm_Res_3, SA_Perm_Res_5, KB_Perm_Res_1, KB_Perm_Res_2, KB_Perm_Res_5, KB_Delta_Sus_2, KB_Delta_Res_5	I/ATC	L/TTG	P/CCG
H8	PQ660311	5	SA_Delta_Sus_1, BP_Perm_Sus_3, BP_Perm_Res_5, BP_Delta_Res_1, KB_Delta_Sus_3	I/ATC	F/TTC	P/CCG
H9	PQ660312	1	SA_Delta_Res_1	I/ATC	F/TTC	P/CCG
**H10**	PQ660313	1	BP_Perm_Res_3	I/ATC	F/TTC	R/CGG
H11	PQ660314	1	BP_Delta_Sus_2	I/ATC	F/TTC	P/CCG
H12	PQ660315	1	BP_Delta_Sus_5	I/ATC	F/TTC	P/CCG
**H13**	PQ660316	2	BP_Delta_Res_2, KB_Perm_Sus_5	I/ATC	F/TTC	R/CGG
H14	PQ660317	2	BP_Delta_Res_3, KB_Perm_Res_4	I/ATC	F/TTC	P/CCG
H15	PQ660318	1	BP_Delta_Res_4	I/ATC	F/TTC	P/CCG
**H16**	PQ660319	5	KB_Perm_Sus_2, KB_Perm_Sus_3, KB_Delta_Res_1, KTT_Perm_Res_4, KTT_Delta_Res_1	I/ATC	L/TTG	P/CCG
**H17****	PQ660320	2	KB_Perm_Res_3, KTT_Delta_Res_5	I/ATC	L/TTG	R/CGG
H18	PQ660321	1	KB_Delta_Sus_1	I/ATC	F/TTC	P/CCG
**H19****	PQ660322	1	KB_Delta_Res_2	I/ATC	L/TTG	R/CGG

H20	PQ660323	1	KB_Delta_Res_3	I/ATC	F/TTC	P/CCG
H21	PQ660324	1	KTT_Perm_Sus_1	I/ATC	F/TTC	P/CCG
**H22**	PQ660325	1	KTT_Perm_Sus_4	I/ATC	F/TTC	R/CGG
**H23**	PQ660326	1	KTT_Perm_Res_2	I/ATC	F/TTC	R/CGG
**H24**	PQ660327	1	KTT_Perm_Res_5	I/ATC	F/TTC	R/CGC
**H25**	PQ660328	1	KTT_Delta_Res_3	I/ATC	F/TTC	R/CGC

*Number of sequences per haplotype. *Largest haplotype group with 28 homologous sequences. **Bold**: Haplotype with mutant alleles conferring knockdown or resistance phenotype. **Bold****: Haplotype with dual mutations.

Additional file 8: Table 13(C): Fifty haplotypes were identified from 90 nucleotide sequences of DIVS6 in *vgsc* gene fragment of local *Ae. albopictus* in Penang and Perlis, and deposited in the NCBI Genbank.

***Haplotype**	**Accession no.**	****No of sequences**	**Individual/sequence identification**	**Alleles**	
				**Codon 1695**	**Codon 1763**
H1	PQ660329	10	USM VCRU_Perm_Sus_1, USM VCRU _Perm_Sus_3, USM VCRU _Perm_Sus_4, USM VCRU _Delta_Sus_4, SA_Perm_Sus_2, KB_Perm_Sus_3, KB_Perm_Res_3, KB_Delta_Res_3, KTT_Perm_Sus_3, KTT_Perm_Sus_5	F/TTC	D/GAC
H2	PQ660330	1	USM VCRU_Perm_Sus_2	F/TTC	D/GAC
H3	PQ660331	2	USM VCRU_Perm_Sus_5, USM VCRU_Delta_Sus_1	F/TTC	D/GAC
H4	PQ660332	5	USM VCRU_Delta_Sus_2, SA_Delta_Res_2, BP_Perm_Sus_5, KB_Perm_Sus_2, KB_Delta_Sus_1	F/TTC	D/GAC
H5*	PQ660333	13	USM VCRU_Delta_Sus_3, USM VCRU_Delta_Sus_5, SA_Perm_Sus_3, SA_Perm_Sus_4, SA_Perm_Sus_5, SA_Perm_Res_2, SA_Delta_Res_3, BP_Perm_Sus_1, BP_Perm_Sus_2, BP_Perm_Res_2, BP_Delta_Sus_4, KB_Delta_Res_1, KTT_Delta_Res_5	F/TTC	D/GAC
H6	PQ660334	1	SA_Perm_Sus_1	F/TTC	D/GAC
**H7**	PQ660335	1	SA_Perm_Res_1	L/CTC	D/GAC
**H8**	PQ660336	1	SA_Perm_Res_3	L/CTC	D/GAC
**H9**	PQ660337	2	SA_Perm_Res_4, SA_Delta_Res_4	L/CTC	D/GAC
**H10**	PQ660338	1	SA_Perm_Res_5	L/CTC	D/GAC

H11	PQ660339	5	SA_Delta_Sus_1, BP_Perm_Sus_4, BP_Delta_Sus_1, KB_Perm_Res_4, KTT_Delta_Res_3	F/TTC	D/GAC
H12	PQ660340	2	SA_Delta_Sus_2, BP_Perm_Sus_3	F/TTC	D/GAC
H13	PQ660341	1	SA_Delta_Sus_3	F/TTC	D/GAC
H14	PQ660342	1	SA_Delta_Sus_4	F/TTC	D/GAC
H15	PQ660343	1	SA_Delta_Sus_5	F/TTC	D/GAC
H16	PQ660344	2	SA_Delta_Res_1, KB_Delta_Res_2	F/TTC	D/GAC
H17	PQ660345	3	SA_Delta_Res_5, KB_Perm_Sus_4, KB_Delta_Sus_4	F/TTC	D/GAC
H18	PQ660346	1	BP_Perm_Res_1	F/TTC	D/GAC
H19	PQ660347	1	BP_Perm_Res_3	F/TTC	D/GAC
**H20**	PQ660348	1	BP_Perm_Res_4	L/CTC	D/GAC
**H21**	PQ660349	1	BP_Perm_Res_5	L/CTC	D/GAC
H22	PQ660350	1	BP_Delta_Sus_2	F/TTC	D/GAC
H23	PQ660351	1	BP_Delta_Sus_3	F/TTC	D/GAC
H24	PQ660352	1	BP_Delta_Sus_5	F/TTC	D/GAC
H25	PQ660353	2	BP_Delta_Res_1, KTT_Perm_Sus_2	F/TTC	D/GAC
H26	PQ660354	1	BP_Delta_Res_2	F/TTC	D/GAC
H27	PQ660355	3	BP_Delta_Res_3, KTT_Delta_Sus_2, KTT_Delta_Res_1	F/TTC	D/GAC
H28	PQ660356	1	BP_Delta_Res_4	F/TTC	D/GAC
**H29**	PQ660357	1	BP_Delta_Res_5	L/CTC	D/GAC
H30	PQ660358	1	KB_Perm_Sus_1	F/TTC	D/GAC
H31	PQ660359	1	KB_Perm_Sus_5	F/TTC	D/GAC
**H32**	PQ660360	1	KB_Perm_Res_1	L/CTC	D/GAC
H33	PQ660361	1	KB_Perm_Res_2	F/TTC	D/GAC
H34	PQ660362	1	KB_Perm_Res_5	F/TTC	D/GAC
H35	PQ660363	1	KB_Delta_Sus_2	F/TTC	D/GAC

H36	PQ660364	1	KB_Delta_Sus_5	F/TTC	D/GAC
H37	PQ660365	1	KB_Delta_Res_3	F/TTC	D/GAC
**H38**	PQ660366	1	KB_Delta_Res_4	L/CTC	D/GAC
H39	PQ660367	1	KB_Delta_Res_5	F/TTC	D/GAC
H40	PQ660368	2	KTT_Perm_Sus_1, KTT_Delta_Sus_1	F/TTC	D/GAC
H41	PQ660369	1	KTT_Perm_Sus_4	F/TTC	D/GAC
H42	PQ660370	2	KTT_Perm_Res_1, KTT_Perm_Res_4	F/TTC	D/GAC
**H43**	PQ660371	1	KTT_Perm_Res_2	L/CTC	D/GAC
H44	PQ660372	1	KTT_Perm_Res_3	F/TTC	D/GAC
H45	PQ660373	1	KTT_Perm_Res_5	F/TTC	D/GAC
H46	PQ660374	1	KTT_Delta_Sus_3	F/TTC	D/GAC
H47	PQ660375	1	KTT_Delta_Sus_4	F/TTC	D/GAC
H48	PQ660376	1	KTT_Delta_Sus_5	F/TTC	D/GAC
H49	PQ660377	1	KTT_Delta_Res_2	F/TTC	D/GAC
H50	PQ660378	1	KTT_Delta_Res_4	F/TTC	D/GAC

*Number of sequences per haplotype. *Largest haplotype group with 13 homologous sequences. **Bold**: Haplotype with mutant alleles conferring knockdown or resistance phenotype.

Haplotype Network (NPM_DIIS6)

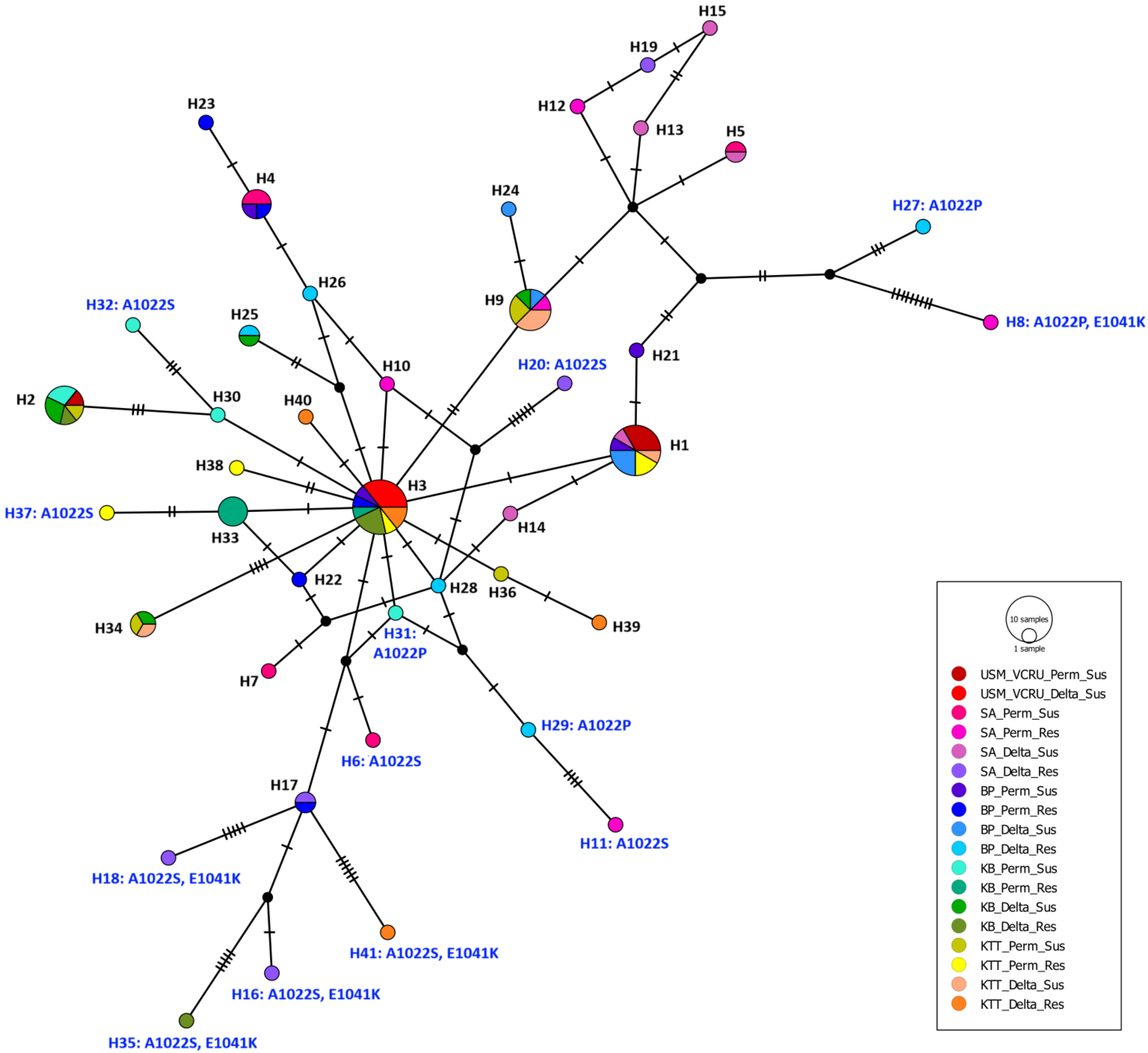

Haplotype Network (NPM_DIVS6)

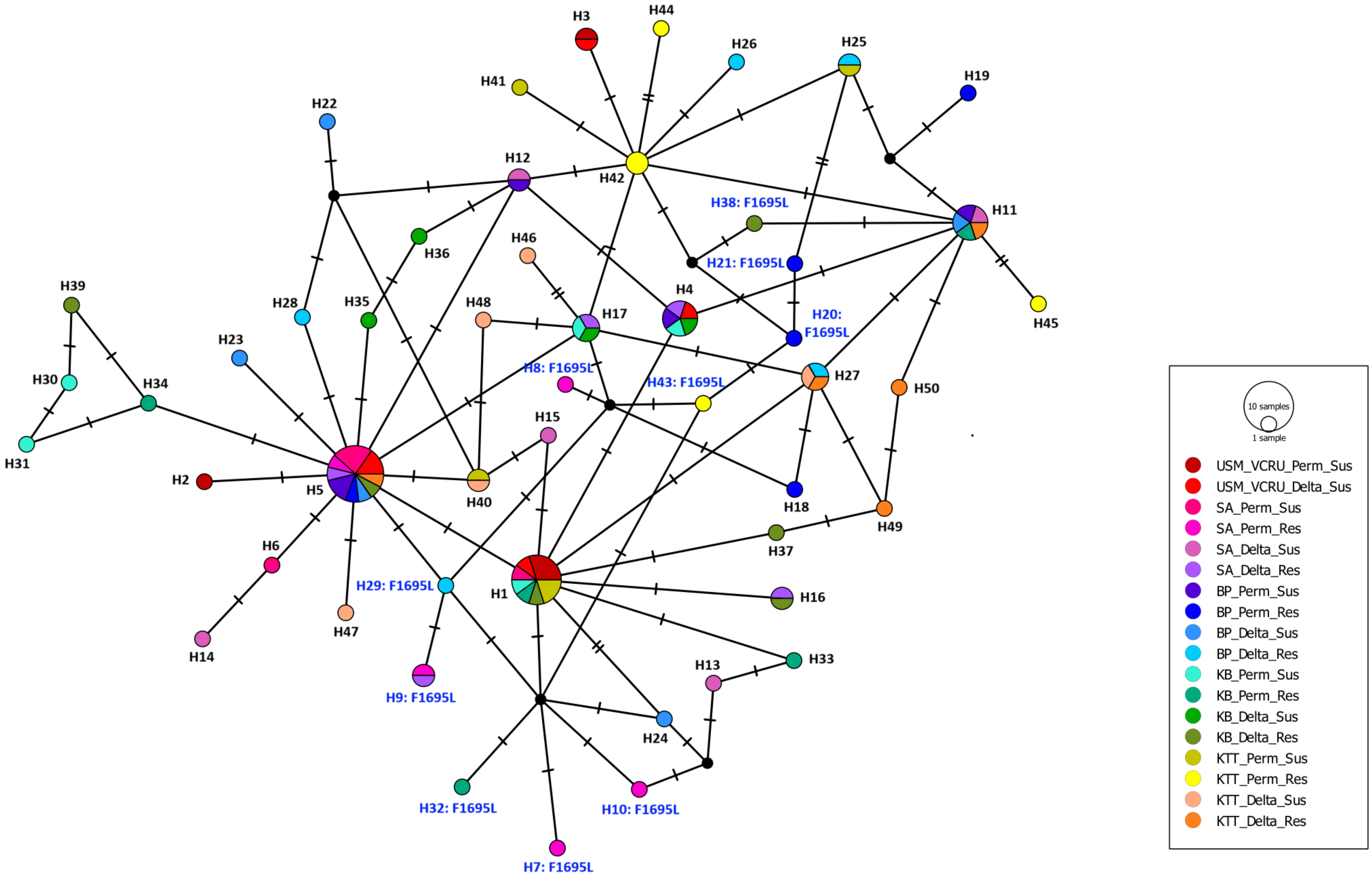

Supplement: Supplementary file 6 [file 13071_2026_7496_MOESM8_ESM.pdf]
