# Supplementary material for: Emerging and regionally novel voltage-gated sodium channel mutations associated with pyrethroid resistance and altered binding affinity in Aedes albopictus (Skuse) from Northern Peninsular Malaysia
Source: Parasit Vectors. 2026 Jun 21;19:321. doi: 10.1186/s13071-026-07496-w (PMC13449526; doi:10.1186/s13071-026-07496-w)

# S5(A). Structural alignment between wild-type and mutants (DIIS6)

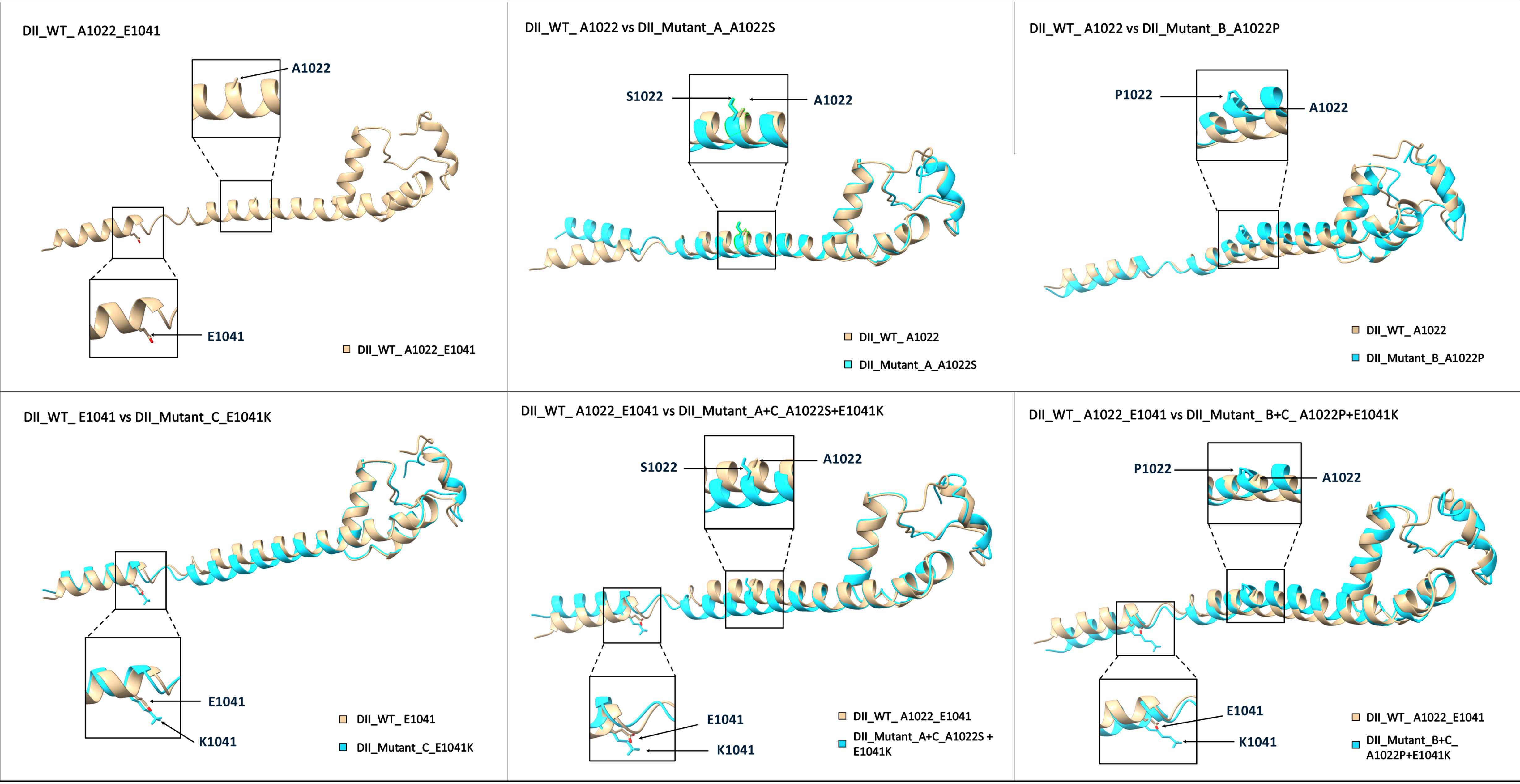

# S5(B) Fig. Structural alignment between wild-type and mutants (DIIIS6)

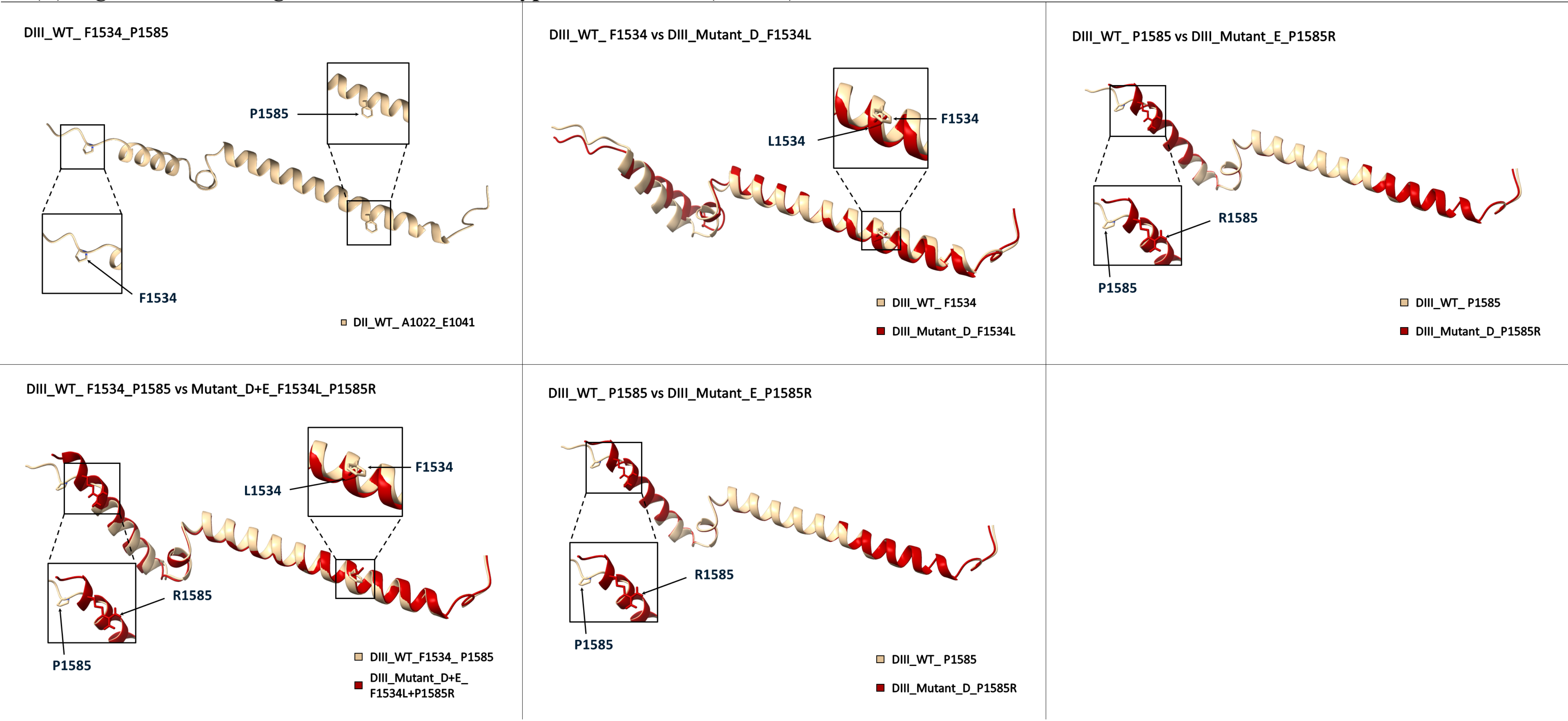

# S6(C) Fig. Structural alignment between wild-type and mutants (DIVS6)

DIV_WT_ F1695 vs DIII_Mutant_F_F1695L

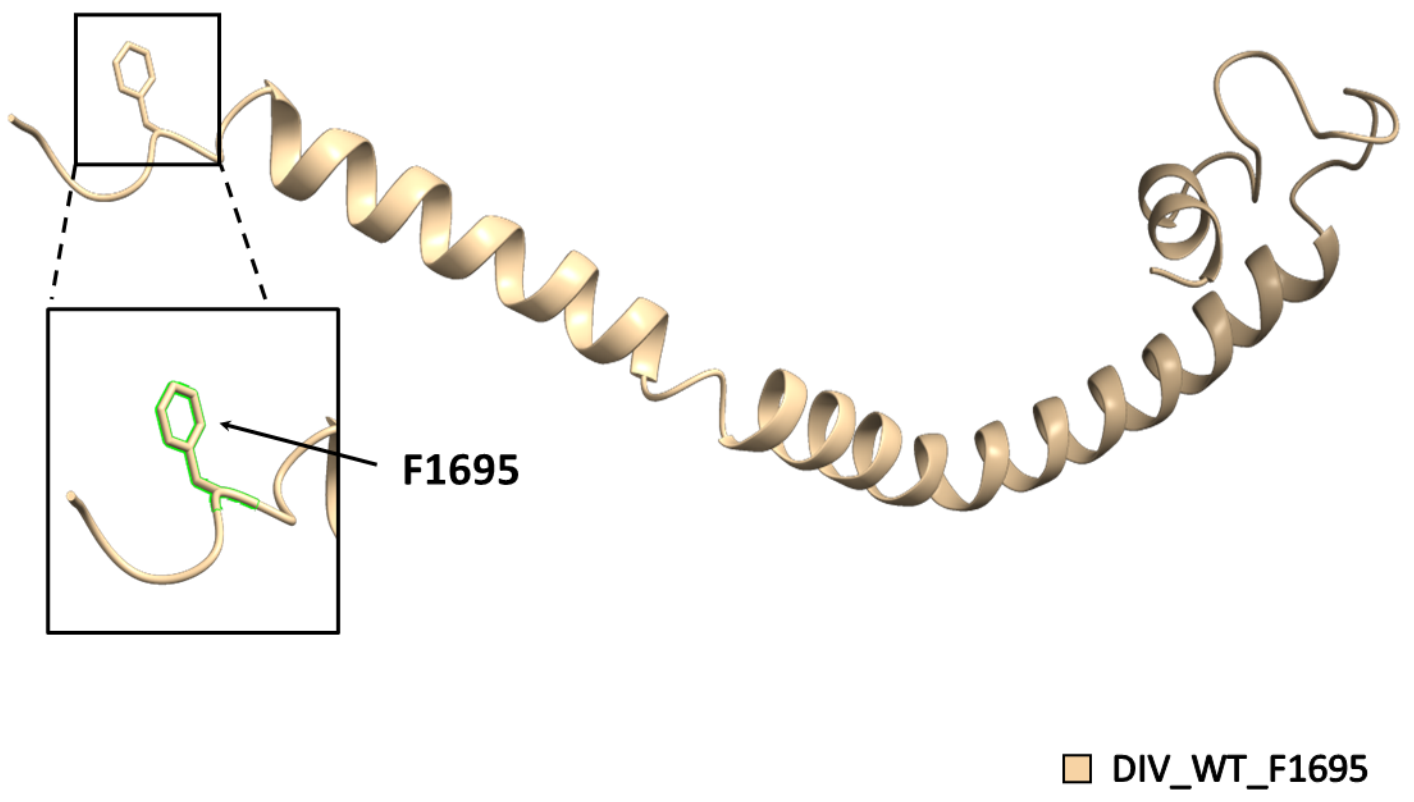


DIV_WT_ F1695 vs DIV_Mutant_F_F1695L

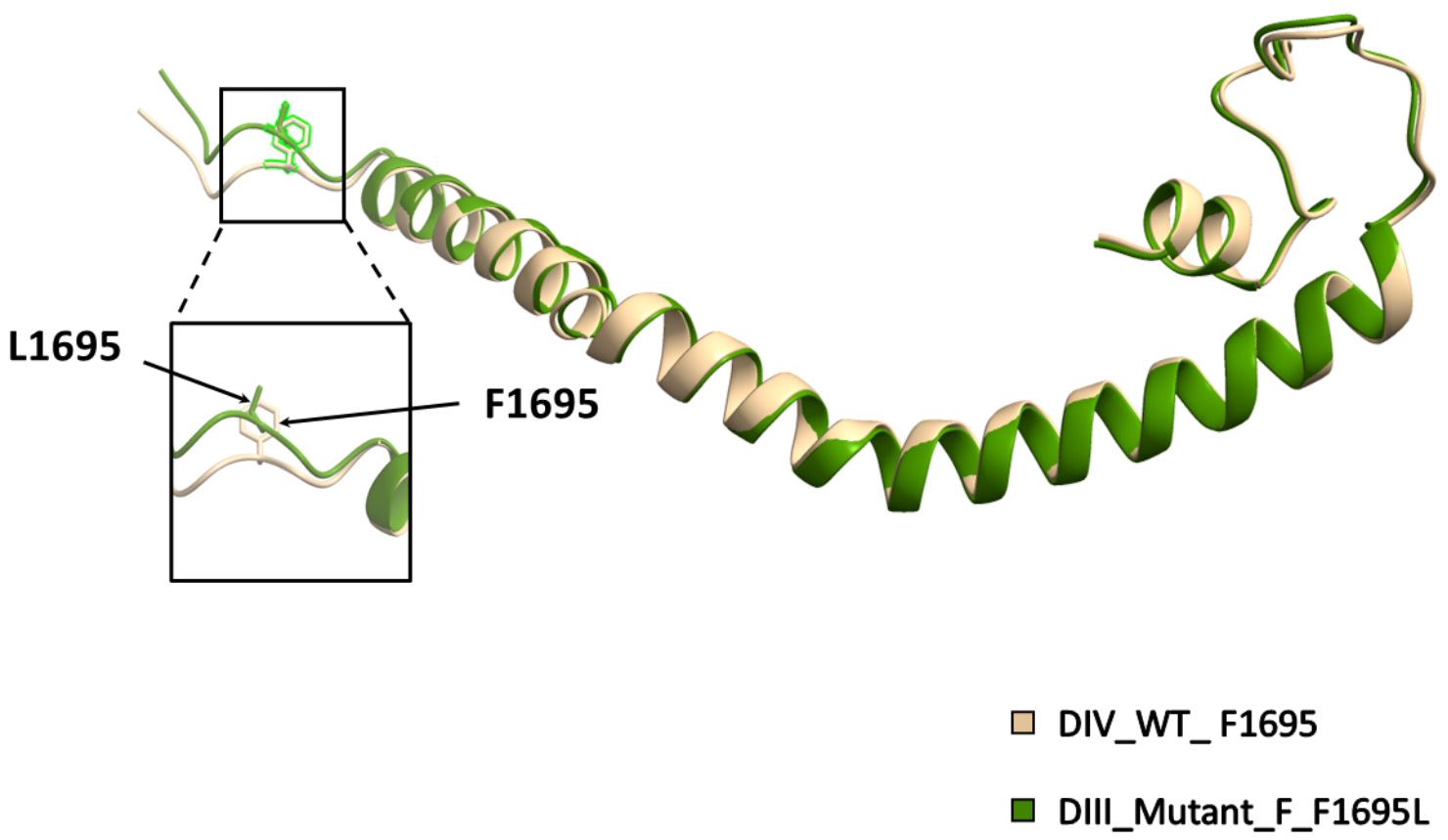

# S6(A) Fig. Positional difference binding of Permethrin againts the wild-type and mutants (DIIS6)

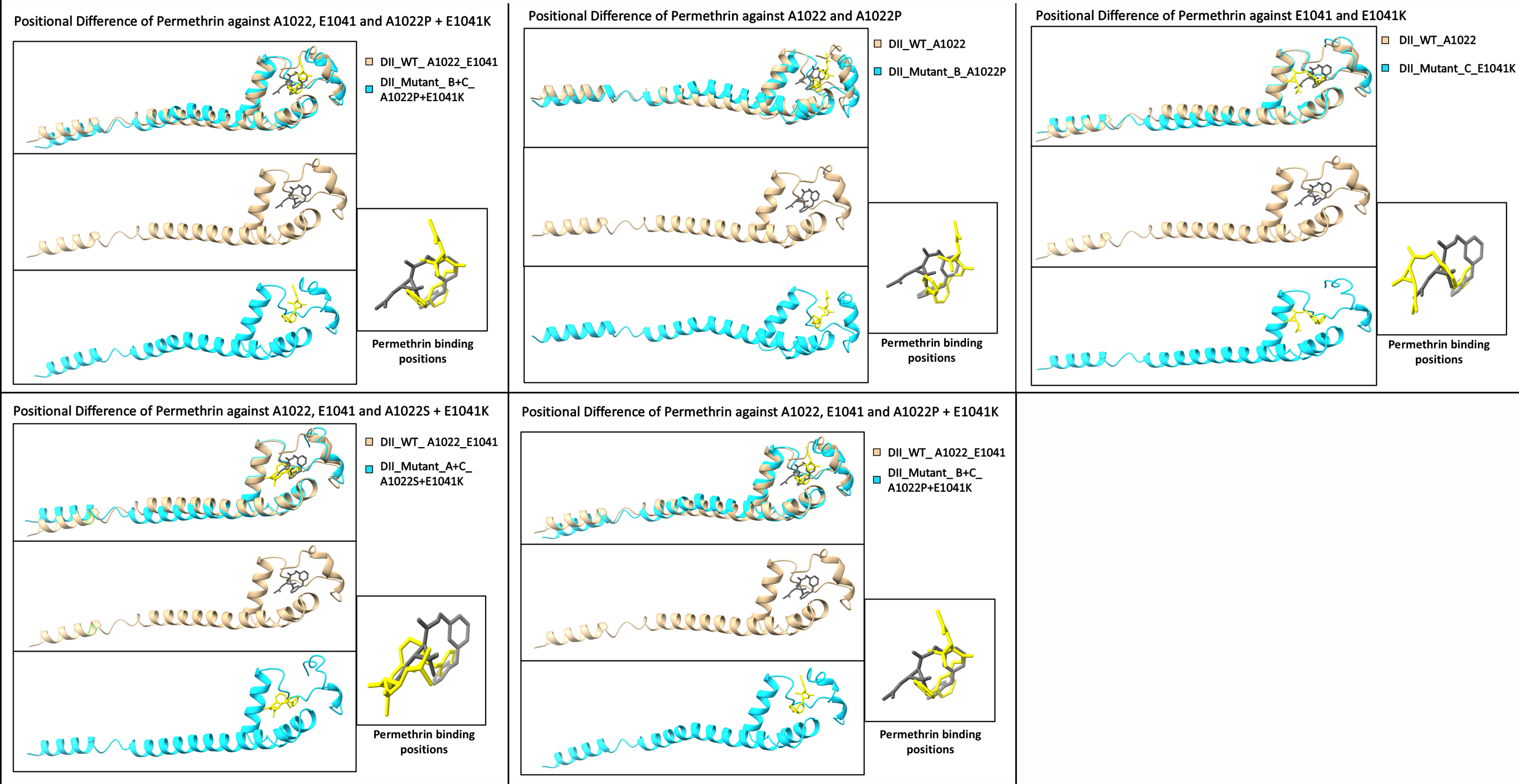

# S6(B) Fig. Positional difference bindings of Permethrin against the wild-type and mutants (DIIIS6)

Positional Difference of Permethrin against F1534 and F1534L

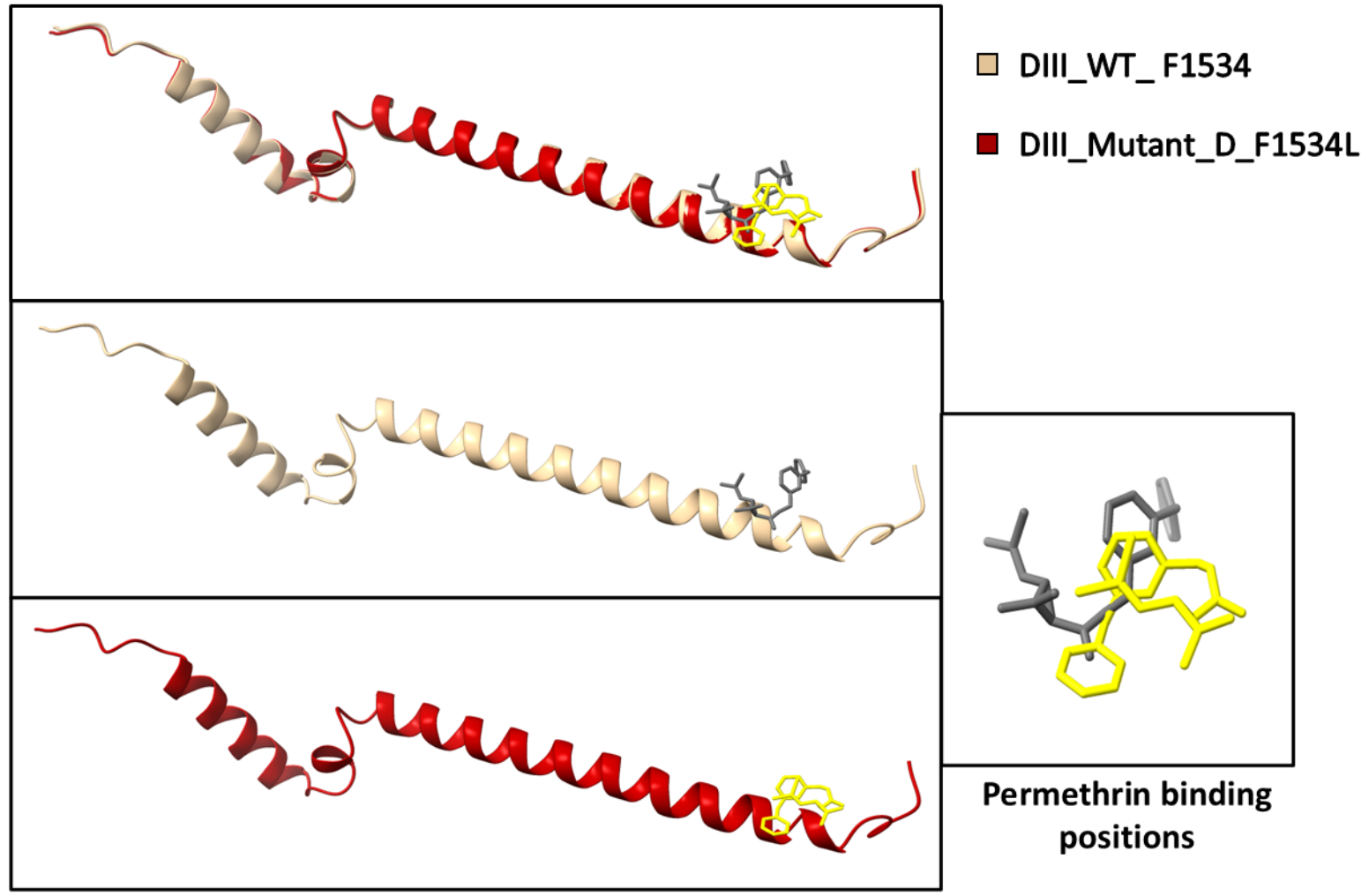


Positional Difference of Permethrin against P1585 and P1585R

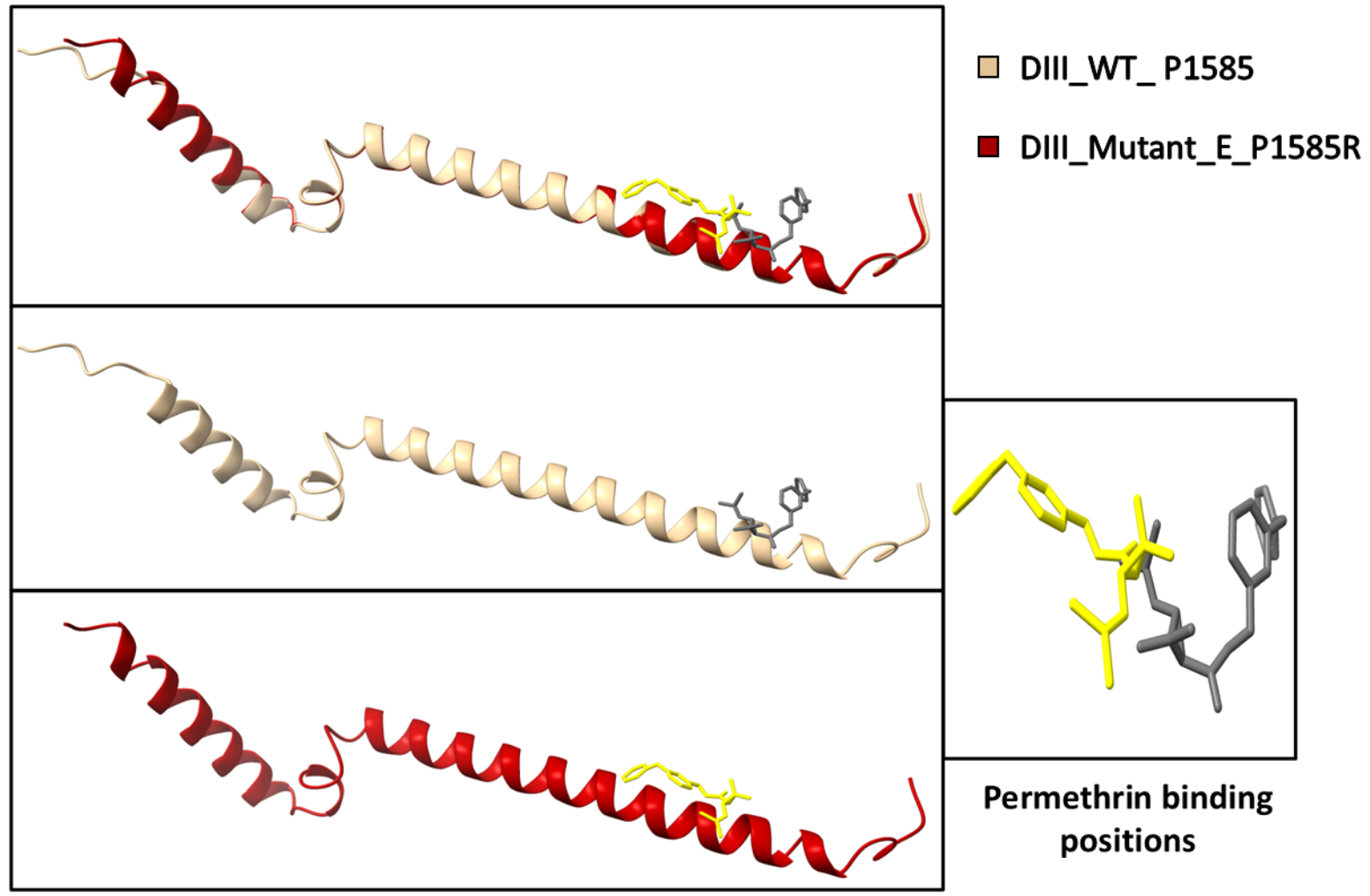


Positional Difference of Permethrin against P1585, F1534 and F1534L+P1585R

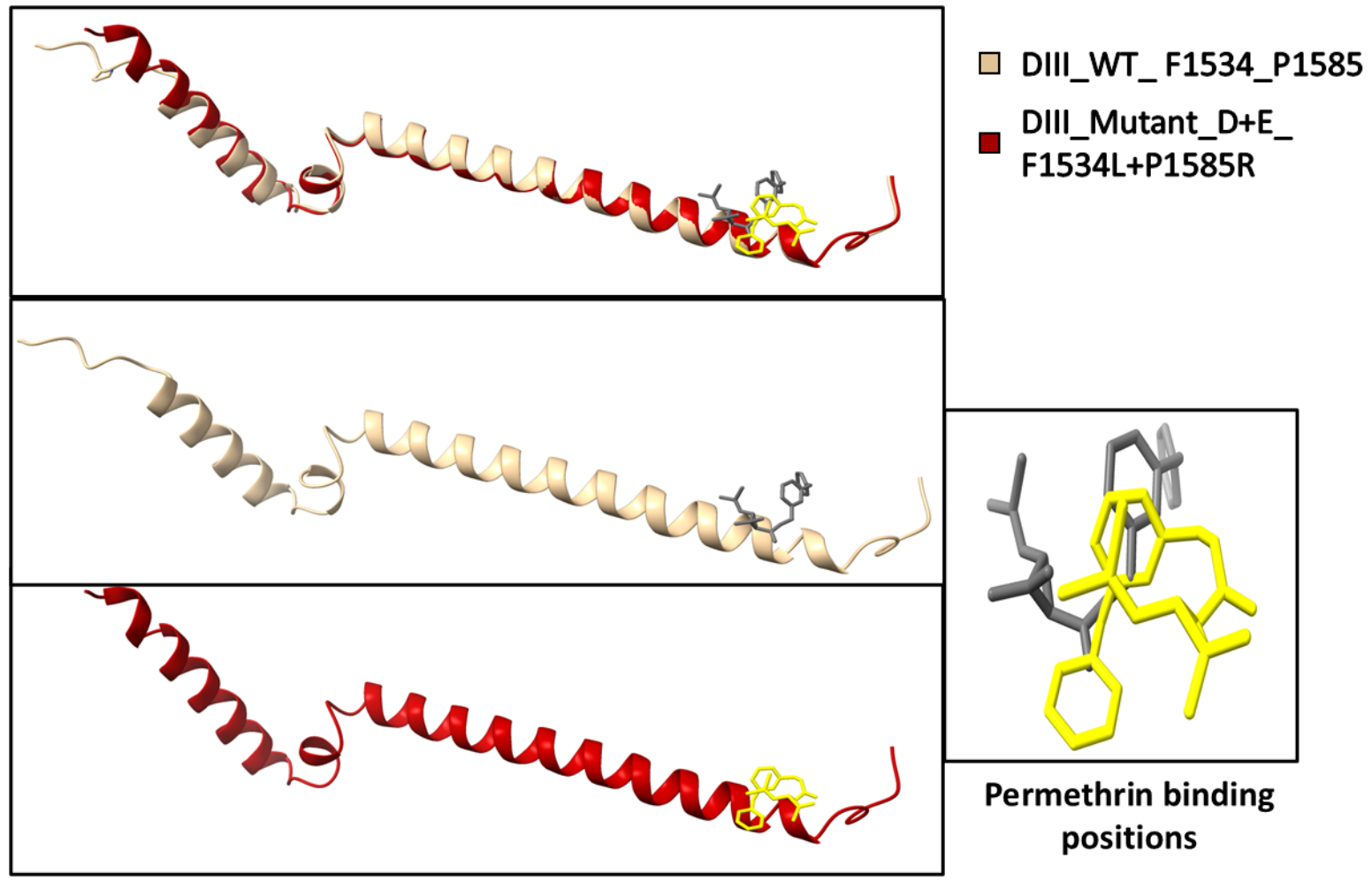

# S6(C) Fig. Positional difference bindings of Permethrin against the wild-type and mutants (DIVS6)

**Positional Difference of Permethrin against F1695 and F1695L**

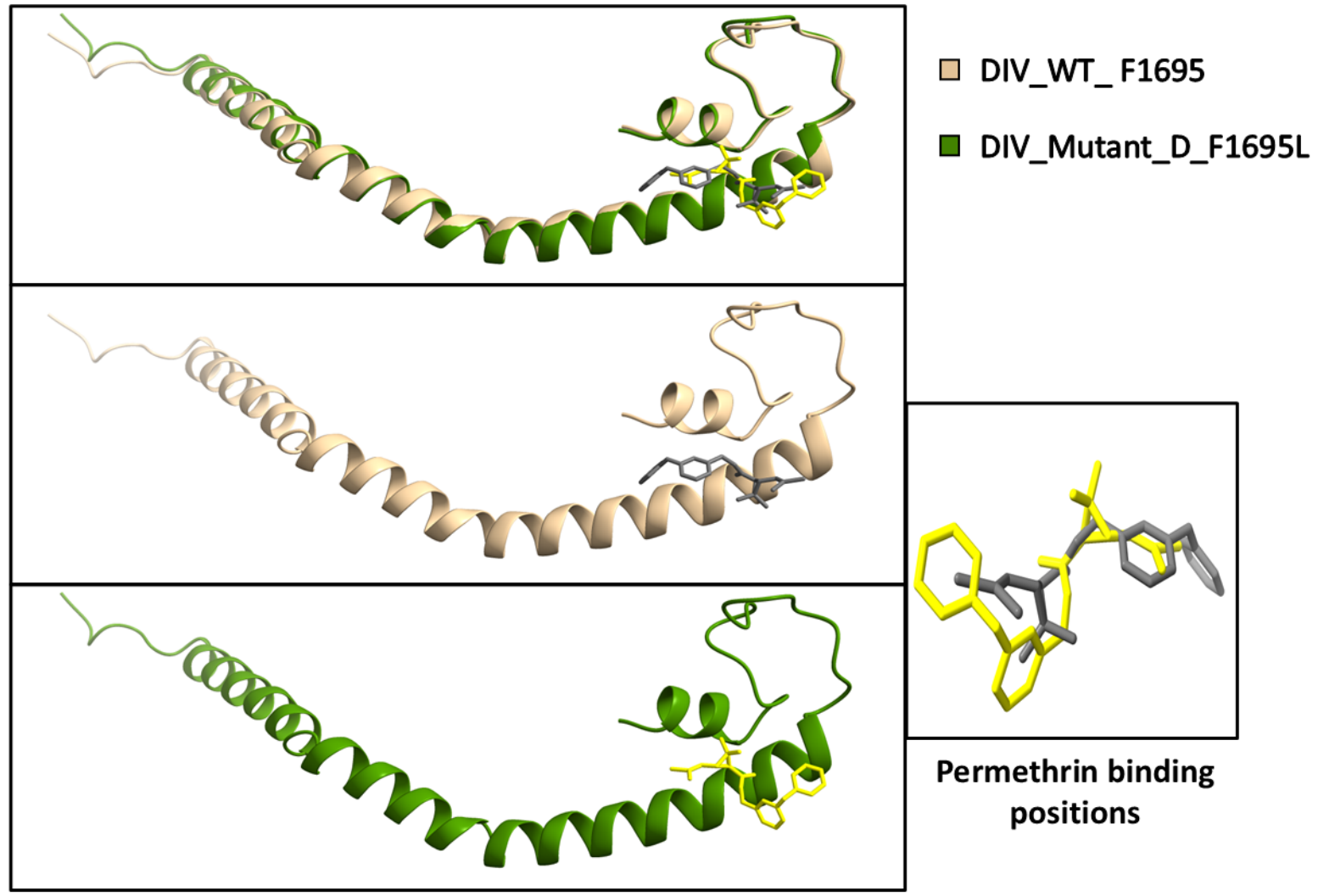

**S6(D) Fig. Positional difference bindings of Deltamethrin against the wild-type and mutants (DIIS6)**

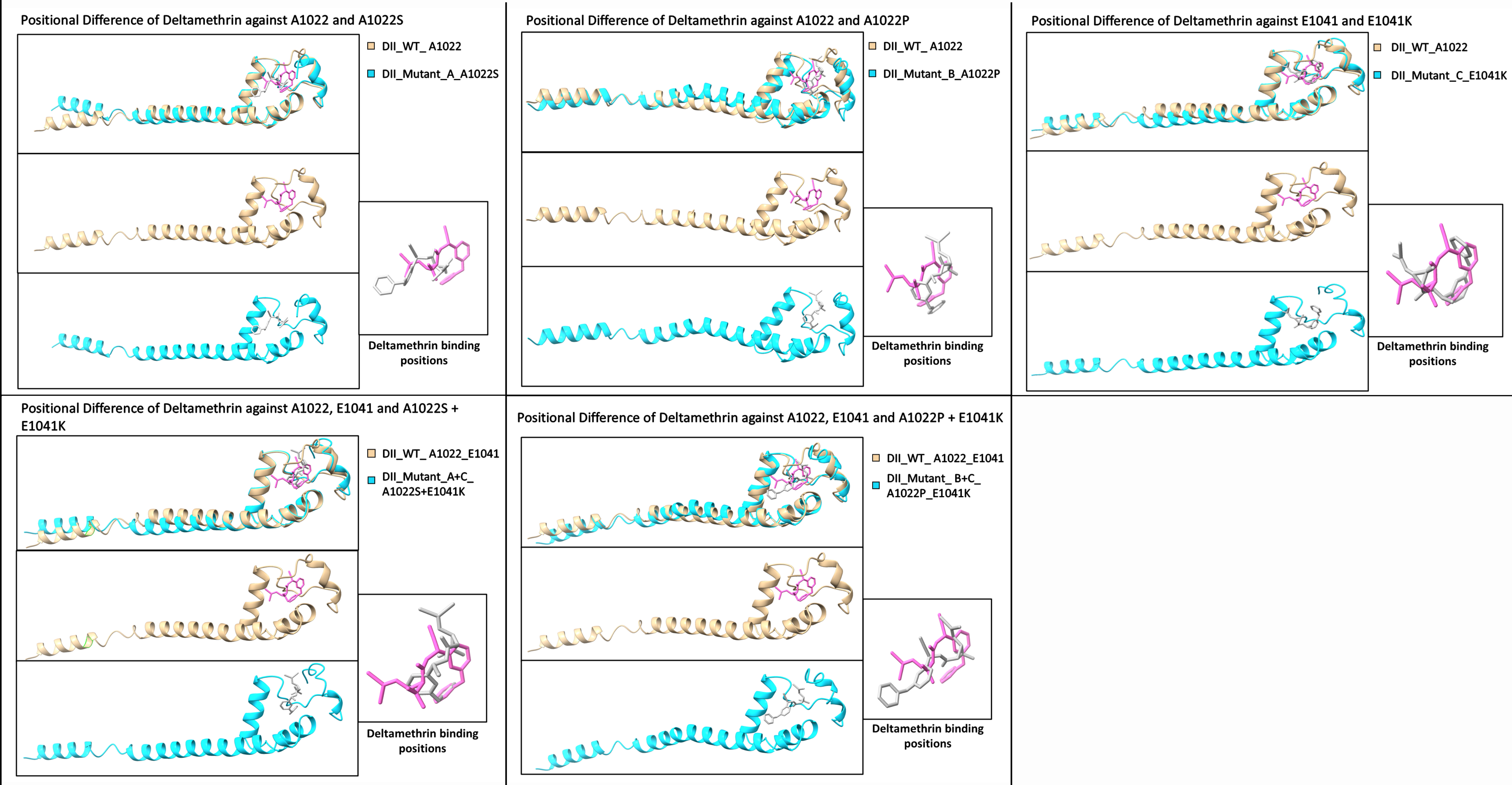

**S6(E) Fig. Positional difference bindings of Deltamethrin against the wild-type and mutants (DIIIS6)**

**Positional Difference of Deltamethrin against F1534 and F1534L**

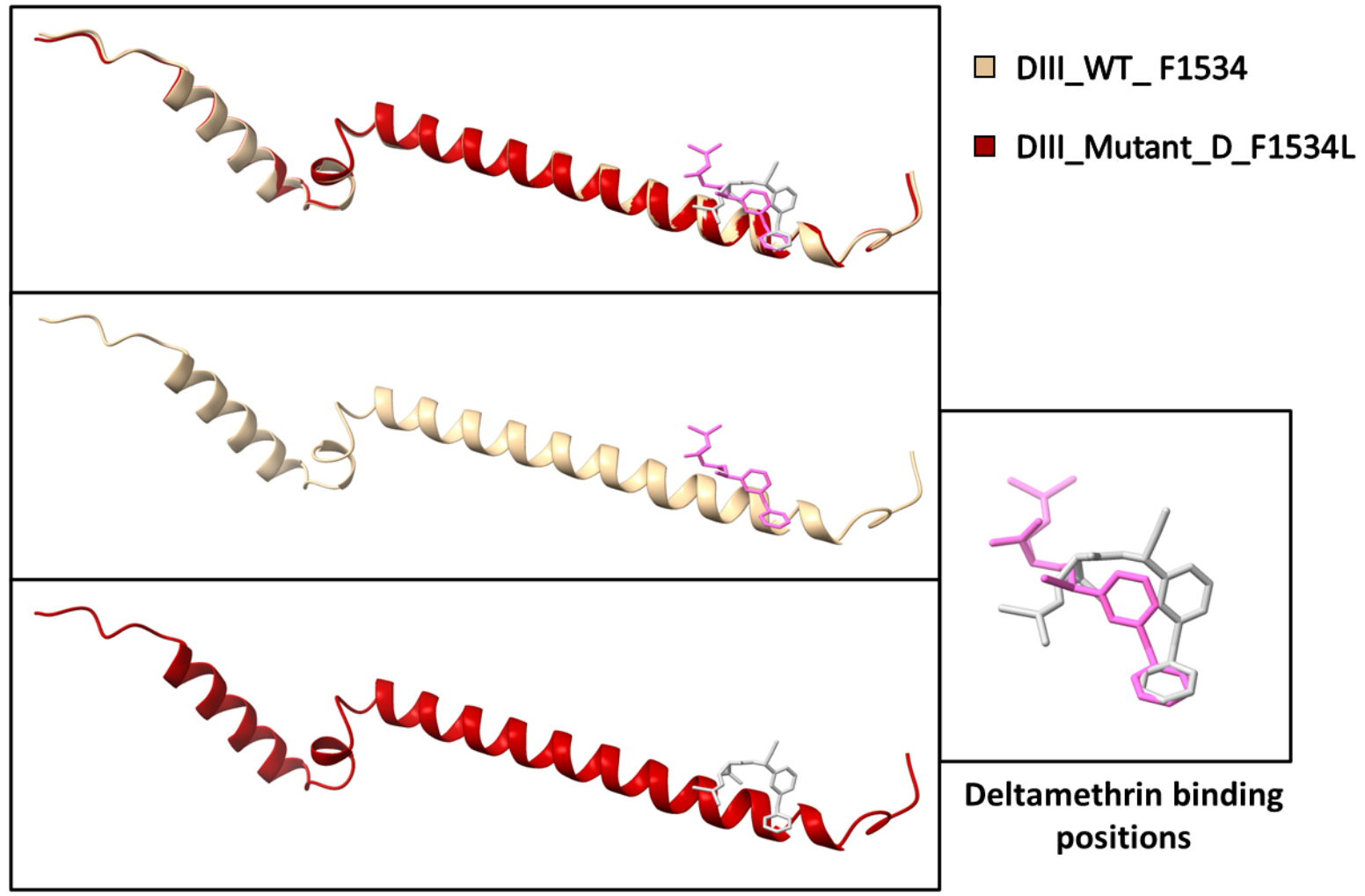


**Positional Difference of Deltamethrin against P1585 and P1585R**

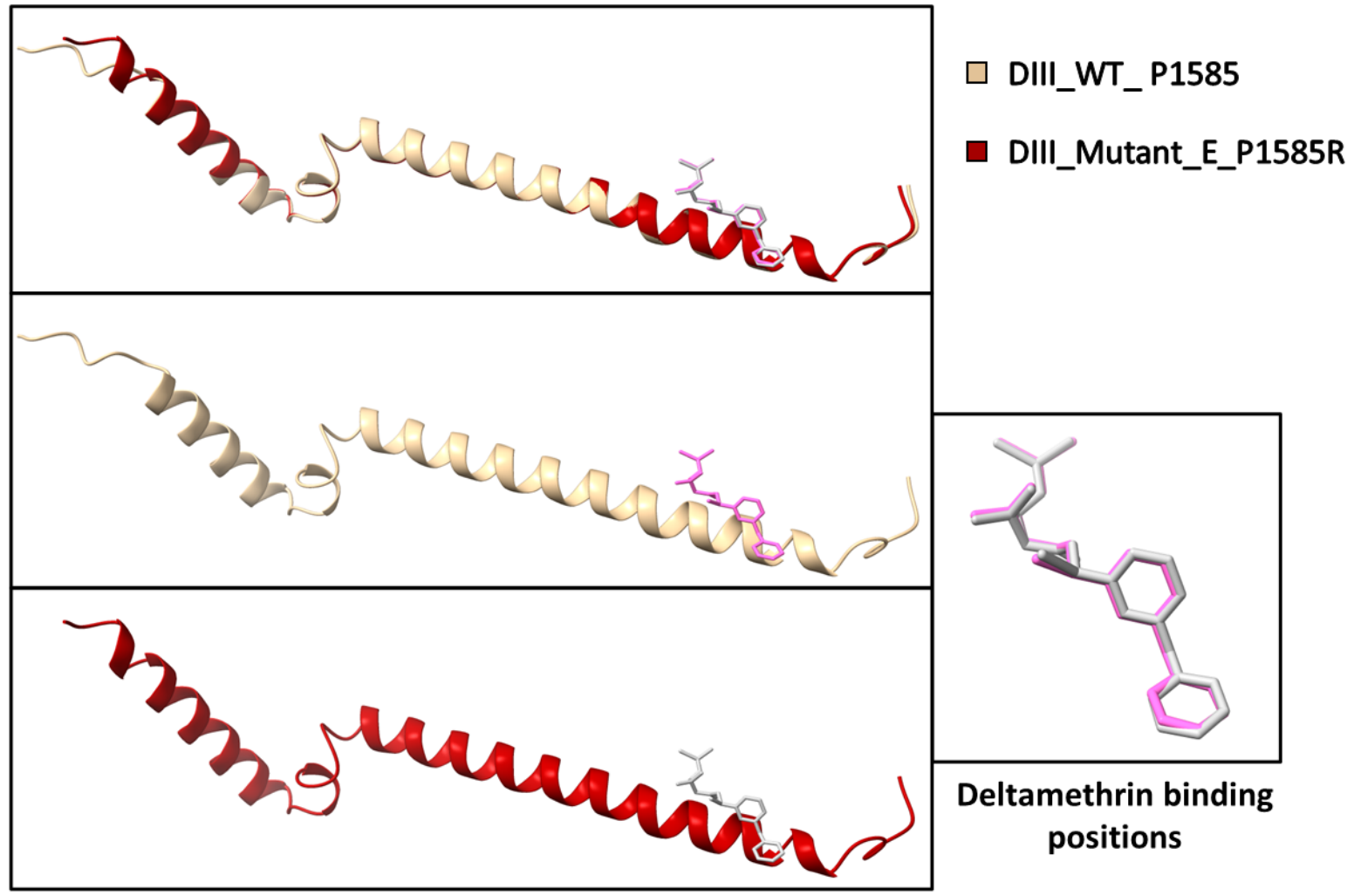


**Positional Difference of Deltamethrin against P1585, F1534 and F1534L+P1585R**

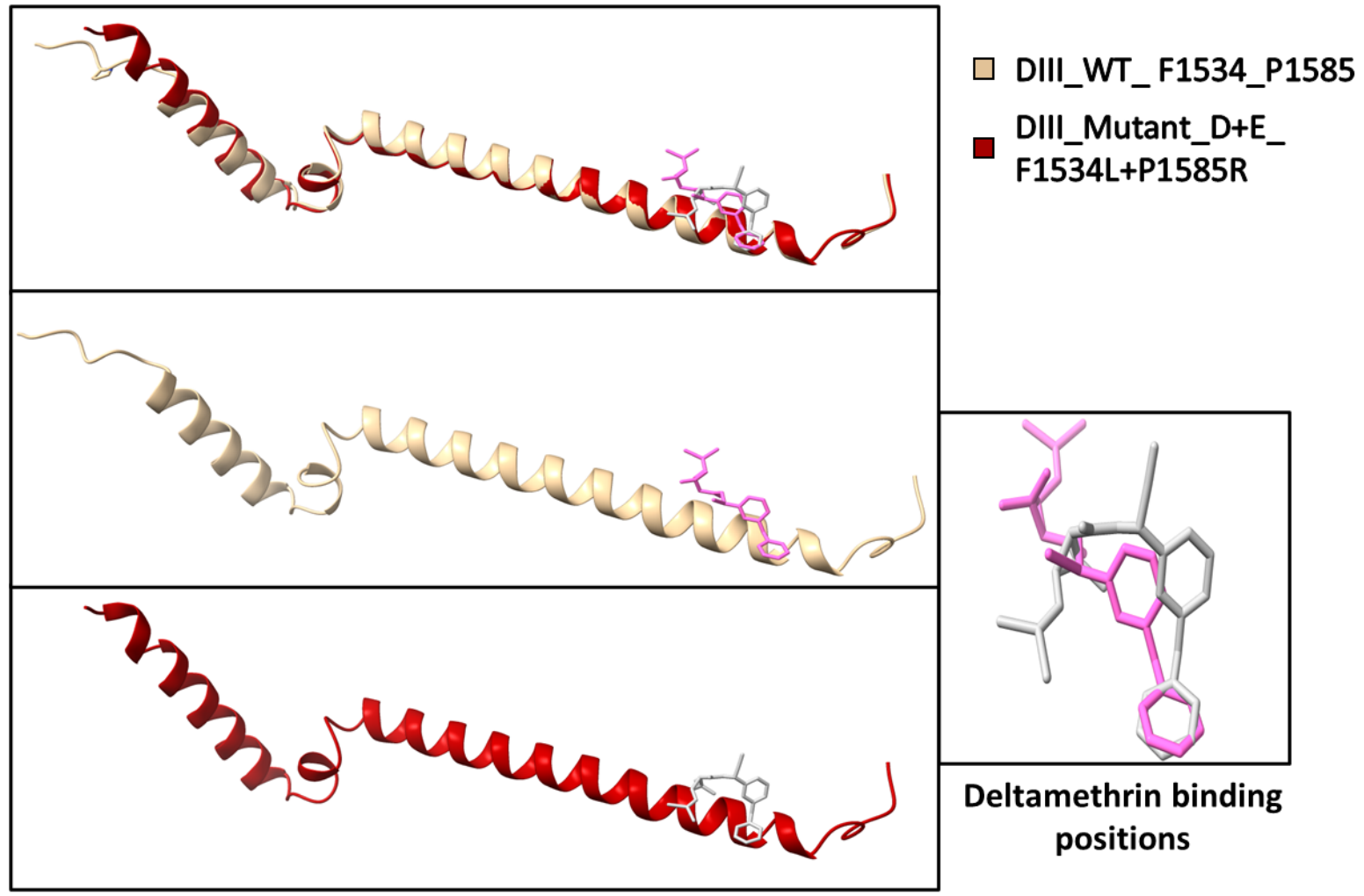

# S6(F) Fig: Positional difference bindings of Deltamethrin against the wild-type and mutant (DIVS6)

**Positional Difference of Deltamethrin against F1695 and F1695L**

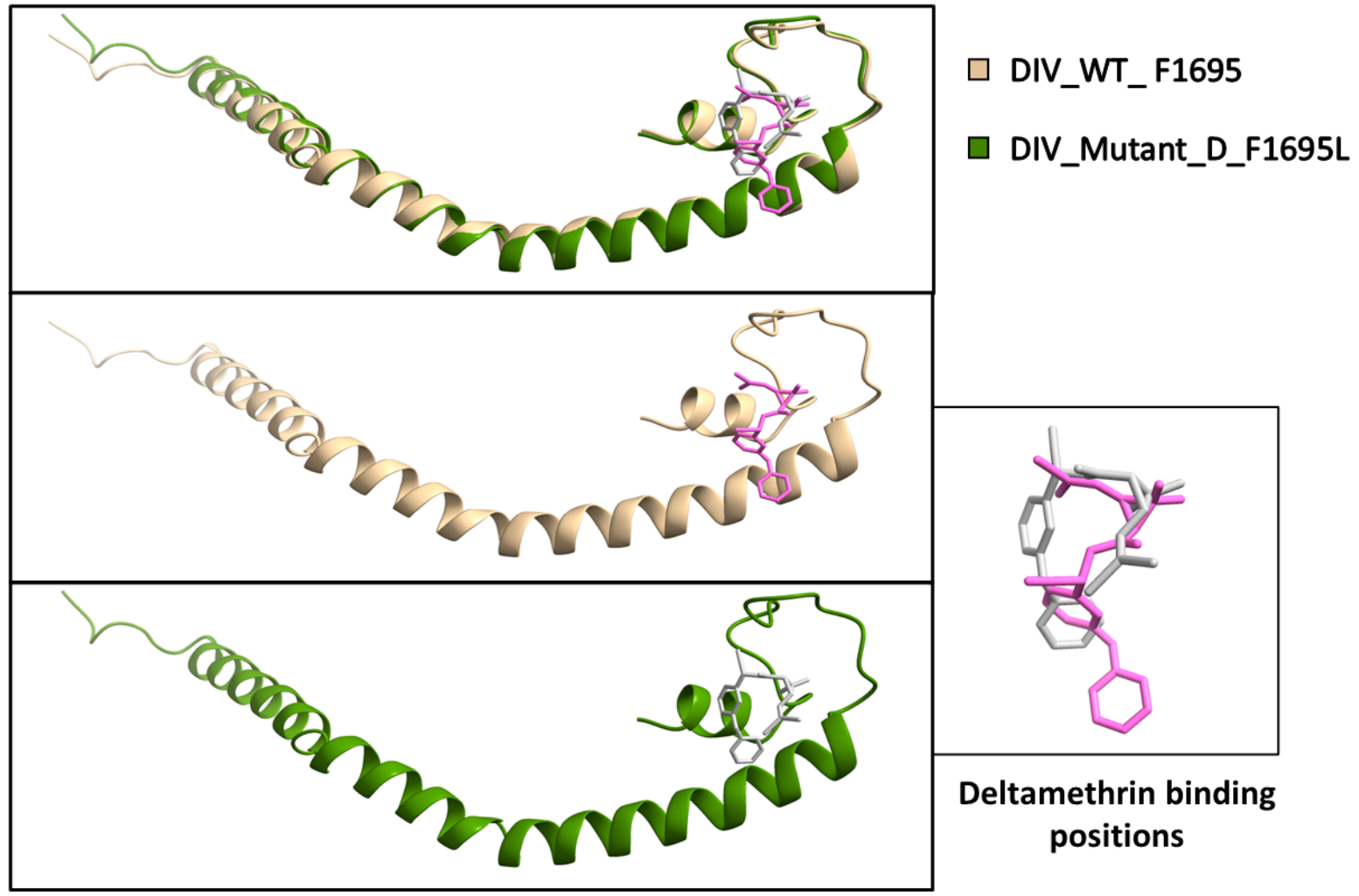

Supplement: Supplementary file 7 [file 13071_2026_7496_MOESM9_ESM.pdf]
